# Semaphorin 5A suppresses ferroptosis through activation of PI3K-AKT-mTOR signaling in rheumatoid arthritis

**DOI:** 10.1038/s41419-022-05065-4

**Published:** 2022-07-14

**Authors:** Qi Cheng, Mo Chen, Mengdan Liu, Xin Chen, Lingjiang Zhu, Jieying Xu, Jing Xue, Huaxiang Wu, Yan Du

**Affiliations:** 1 Department of Rheumatology, The Second Affiliated Hospital of Zhejiang University School of Medicine, 88 Jiefang Road, 310009 Hangzhou, China; 2 Department of Clinic Medicine, The Second Affiliated Hospital of Zhejiang University School of Medicine, 88 Jiefang Road, 310009 Hangzhou, China; 3 Department of Neurology, Linping District Hospital of Integrated Traditional Chinese and Western Medicine, 311199 Hangzhou, Zhejiang China

**Keywords:** Autoimmunity, Cell death and immune response, Signal transduction, Inflammation

## Abstract

Abnormal activation of synovial fibroblasts (SFs) plays an important role in rheumatoid arthritis (RA), the mechanism of which remains unknown. The purpose of our study is to comprehensively and systematically explore the mechanism for Semaphorin 5A-mediated abnormal SF activation in RA. Here, we found that Semaphorin 5A levels were significantly higher in synovial fluid and synovial tissue from RA patients compared with osteoarthritis patients. We further found that the mRNA level and protein abundance of Plexin-A1 was elevated in RA SFs compared with OA SFs, while Plexin-B3 expression showed no significant difference. The increased Semaphorin 5A in RA synovial fluid was mainly derived from CD68^+^ synovial macrophages, and the elevation led to increased binding between Semaphorin 5A and its receptors, thereby promoting cytokine secretion, proliferation, and migration, and decreasing apoptosis. Moreover, the effect of Semaphorin 5A on enhancing activation (cytokine secretion, cell proliferation and migration) and reducing apoptosis of SFs was significantly abolished after knockdown of Plexin-A1 and Plexin-B3 by small interfering RNA. Transcriptome sequencing and protein array detection revealed that Semaphorin 5A activated the PI3K/AKT/mTOR signaling pathway and inhibited ferroptosis. Morphologically, transmission electron microscopy results showed that Semaphorin 5A could significantly eliminate the mitochondrial diminution, membrane density increased and crest ruptured of SFs induced by ferroptosis inducer RSL3. Mechanistically, Semaphorin 5A enhanced GPX4 expression and SREBP1/SCD-1 signaling by activating the PI3K/AKT/mTOR signaling pathway, thus suppressing ferroptosis of RA SFs. In conclusion, our study provided the first evidence that elevated Semaphorin 5A in RA synovial fluid promotes SF activation by suppressing ferroptosis through the PI3K/AKT/mTOR signaling pathway.

## Introduction

Rheumatoid arthritis (RA) is a systemic autoimmune disease with synovial inflammation as the main pathological feature, and can eventually lead to irreversible joint or organ damage [1]. Synovial macrophages (SMs) and synovial fibroblasts (SFs) play important roles in the occurrence and development of synovitis by secreting large amounts of cytokines and conducting crosstalk with various cells [2–4]. However, understanding of the synovial inflammatory microenvironment and the mechanism for synovial cell activation remains limited, and there are no effective treatment methods that directly target synovial cells.

Semaphorin 5A is a member of the Semaphorin family that has close associations with various pathophysiological phenomena including cell migration, tumor growth, and immune responses [5]. In previous studies by ourselves and Gras et al., Semaphorin 5A was confirmed to be significantly elevated in serum from patients with systemic lupus erythematosus (SLE) and RA [6–8]. The pathogenesis of the Semaphorin 5A involvement in RA has been partially revealed. Specifically, Gras et al. [7] reported that soluble Semaphorin 5A strongly induces proliferation and activation of T cells and NK cells and promotes their secretion of a variety of pro-inflammatory factors. Xiao et al. [9] further reported that TSP1 is the key domain of Semaphorin 5A involved in fibroblast-like synovial cell proliferation and angiogenesis. SFs not only promote inflammation, angiogenesis, and bone destruction, but also exhibit tumor cell-like phenotypes, such as metabolic reprogramming, increased invasion, and migration, and decreased apoptosis [2, 10, 11]. Therefore, a comprehensive and systematic understanding of the effects of Semaphorin 5A on SFs is needed for better understanding of the disease pathophysiology in RA.

Recently, a newly discovered form of programmed cell death, ferroptosis, has attracted much attention because of its potential for application in cancer treatment strategies, and is characterized by lipid peroxidation and iron overload [12]. Li et al. [13] reported that neutrophil ferroptosis is an important driver of SLE neutropenia and contributes significantly to the disease presentation. Given the tumor cell-like phenotypes of RA synovial cells, we hypothesize that ferroptosis plays an important role in synovial cell activation. However, there are few studies on the role of ferroptosis in the pathogenesis of RA.

In the present study, we first identified SMs as the mainly origin of the increased Semaphorin 5A in RA synovial fluid and found a crosstalk in SMs and SFs which Semaphorin 5A secreted by macrophages may bind to Plexin receptors on SFs to exert its effect. Next, we specifically investigated the mechanism of Semaphorin 5A mediates the activation of SFs by high-throughput sequencing and in vitro validation and the role of Semaphorin 5A in ferroptosis of RA SFs.

## Results

### Semaphorin 5A is elevated in RA, especially in SMs

**Fig. 1:**
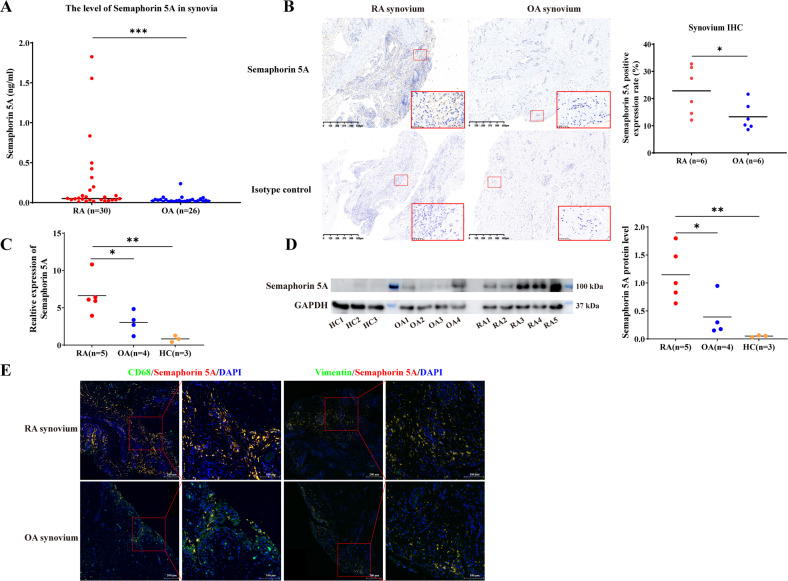
Semaphorin 5A is elevated in RA, especially in SMs. **A** Levels of Semaphorin 5A in synovial fluid from 30 RA patients and 26 OA patients. **B** Immunohistochemistry of synovial tissues from RA and OA patients. The Semaphorin 5A positive expression rate was significantly higher in RA synovial tissues (*n* = 6) compared with OA synovial tissues (*n* = 6). **C**, **D** Expression levels of Semaphorin 5A in RA (*n* = 5), OA (*n* = 4) and healthy control (*n* = 3) synovial tissues detected by PCR (**C**) and western blotting (**D**). **E** Representative image of double-staining immunofluorescence of synovial tissues. Semaphorin 5A expression was higher in RA SMs (CD68^+^) compared with OA SMs (*n* = 3), but there was no difference between RA SFs (Vimentin^+^) and OA SFs (*n* = 3). *P < 0.05; **P < 0.01. Values of *P* < 0.05 were considered significant. RA rheumatoid arthritis, OA osteoarthritis, SMs synovial macrophages, SFs synovial fibroblasts.

To evaluate Semaphorin 5A expression in RA and its clinical and functional significance, we collected synovial fluid samples from 30 RA patients and 26 OA patients (Clinical and laboratory features of patients were shown in Supplementary Table 1) and examined the levels of Semaphorin 5A by ELISA. Consistent with our previous results in serum [6], the level of Semaphorin 5A in synovial fluid was significantly increased in RA compared with OA (Fig. 1A). In addition, we found a significant positive correlation between Semaphorin 5A in synovial fluid and Erythrocyte Sedimentation Rate, C-reactive protein (CRP), Rheumatoid Factor and Disease activity score of 28 joints (CRP), which are indicators of patients’ disease activity (Supplementary Table 2). Because the most basic pathological manifestation of RA is synovial inflammation, SFs and SMs are the most important effector cells. Therefore, we obtained synovial tissue specimens from RA and OA patients (Clinical and laboratory features of patients were shown in Supplementary Table 3) and detected the expression of Semaphorin 5A by immunohistochemistry, qPCR, and western blotting. Interestingly, we found that Semaphorin 5A was more strongly expressed in RA synovial tissues compared with OA synovial tissues (Fig. 1B-D). Both SFs and SMs can secrete a variety of inflammatory or effector molecules into the synovial fluid to promote the inflammation process [2]. To explore the reasons for the elevated levels of Semaphorin 5A in synovial fluid, we focused on these two major effector cells for synovial inflammation. Tissue immunofluorescence staining revealed that Semaphorin 5A expression was higher in RA SMs (CD68^+^) than in OA SMs, but there was no difference between RA SFs (Vimentin^+^) and OA SFs (Fig. 1E). These findings suggest that the difference of Semaphorin 5A in RA synovial fluid is mostly derived from SMs rather than SFs. Semaphorin 5A tends to act through binding to receptors, and Plexin-A1 and Plexin-B3 were identified as its functional receptors [14, 15]. To understand which effector cells are the targets of the elevated Semaphorin 5A in synovial fluid, we investigated the expression of the Plexin receptors on SMs and SFs using the publicly available Single-cell RNA-seq dataset (ImmPort: SDY998) [16]. The expression of Plexin-A1 was significantly higher in SFs than in SMs, B cells, and T cells (Supplementary Fig. 1A–D). Unfortunately, Plexin-B3 was not found in this dataset. We further found that the mRNA level and protein abundance of Plexin-A1 was elevated in RA SFs compared with OA SFs, while Plexin-B3 expression showed no significant difference (Supplementary Fig. 1E–G). In addition, Semaphorin 5A and Plexin-A1 co-located on the membrane of SFs. These results suggest that Semaphorin 5A secreted by macrophages may bind to Plexin-A1 receptors on SFs to exert its effect.

### Semaphorin 5A promotes activation and decreases apoptosis of SFs

**Fig. 2:**
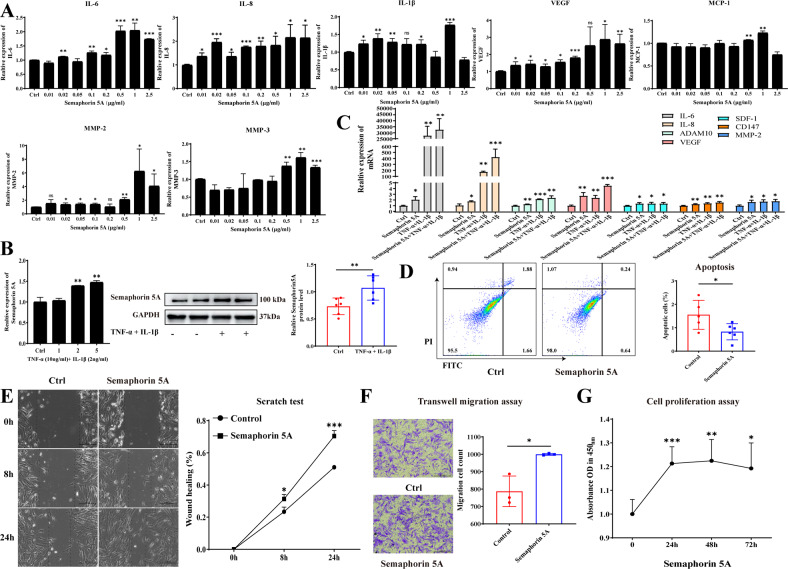
Semaphorin 5A promotes cytokine secretion, cell proliferation, and cell migration and decreases apoptosis of SFs. **A** mRNA levels of various cytokines including inflammatory cytokines (IL-6, IL-8, IL-1β), angiogenesis-related factors (VEGF, MCP-1), and matrix metalloproteinases (MMP-2, MMP-3) in SFs stimulated with different concentrations of Semaphorin 5A (*n* = 3). **B** mRNA levels and protein abundances of Semaphorin 5A in SFs activated by TNF-α (10 ng/mL) and IL-1β (2 ng/mL) were detected by qPCR (*n* = 3) and western blotting (*n* = 6). **C** mRNA levels of IL-6, IL-8, ADAM10, VEGF, SDF-1, CD147, and MMP-2 in SFs synergistically stimulated with Semaphorin 5A (1 μg/mL), TNF-α (10 ng/mL), and IL-1β (2 ng/mL) (*n* = 3). **D** Apoptosis of SFs detected by flow cytometry (*n* = 6) after treatment with Semaphorin 5A (1 μg/mL). **E** Migration of SFs detected by wound healing assays (*n* = 3) after treatment with Semaphorin 5A (1 μg/mL). **F** Migration of SFs detected by transwell migration assays (*n* = 3) after treatment with Semaphorin 5A (1 μg/mL). **G** Proliferation of SFs detected by a Cell Counting Kit-8 (*n* = 4–5) after treatment with Semaphorin 5A (1 μg/mL). *P < 0.05; **P < 0.01; ***P < 0.001, ns, not significant. Values of *P* < 0.05 were considered significant. SFs synovial fibroblasts.

Next, we examined the effects of exogenous Semaphorin 5A on MH7A. Interestingly, we found that exogenous Semaphorin 5A markedly stimulated SFs to secrete a variety of cytokines, including inflammatory cytokines (IL-6, IL-8, IL-1β), angiogenesis-related factors (VEGF, MCP-1), matrix metalloproteinases (MMP-2, MMP-3), and others (ADAM10, SDF-1, CD147) at the optimal concentration and timing (Fig. 2A, Supplementary Fig. 2A, B). More reliably, we obtained the same results in primary human SFs isolated from RA patients (Supplementary Fig. 2C). We further found a significant increase in the RNA level and protein abundance of Semaphorin 5A in SFs activated by TNF-α and IL-1β (Fig. 2B). Meanwhile, Semaphorin 5A acted synergistically with TNF-α and IL-1β to promote the secretion of cytokines from SFs (Fig. 2C). RA SFs exhibited tumor-like properties, such as increased proliferation and migration and decreased apoptosis, and Semaphorin 5A significantly promoted these characteristics (Fig. 2D–G). In addition, Semaphorin 5A was found to significantly inhibit Staurosporine-induced apoptosis (Supplementary Fig. 2D). These findings suggest that Semaphorin 5A can enhance activation and reduce apoptosis of SFs, promoting the production of an inflammatory synovial microenvironment.

### Semaphorin 5A promotes activation of SFs by binding to its receptors Plexin-A1 and Plexin-B3

**Fig. 3:**
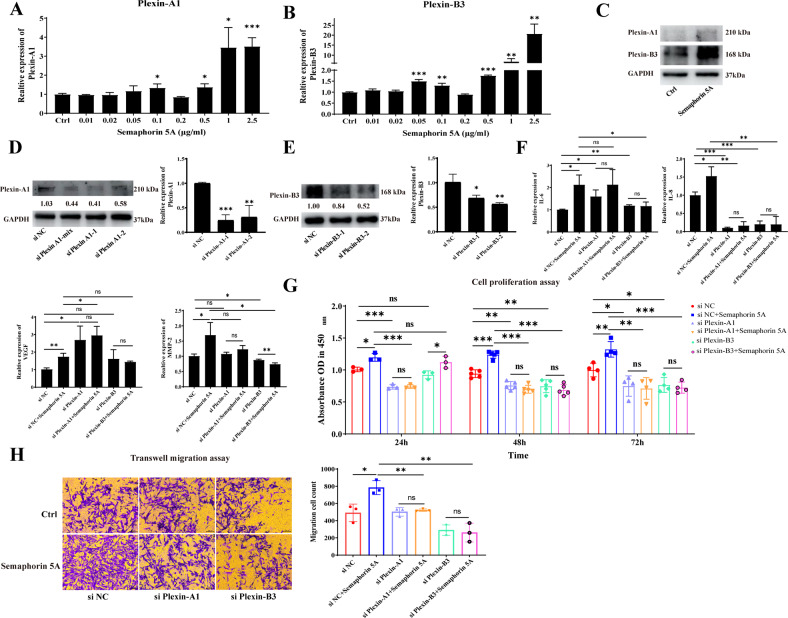
Semaphorin 5A promotes activation of SFs by binding to plexin-A1 and plexin-B3. **A**–**C** RNA levels and protein abundances of Plexin-A1 and Plexin-B3 in SFs detected by qPCR (*n* = 3) (**A**, **B**) and western blotting (*n* = 3) (**C**). **D**, **E** After transfection of siRNAs to knock down Plexin-A1 (**D**) and Plexin-B3 (**E**), the RNA levels and protein abundances of Plexin-A1 and Plexin-B3 in SFs were significantly decreased (*n* = 3). **F** mRNA levels of IL-6, IL-8, VEGF, and MMP-2 in SFs detected by qPCR (*n* = 3) after transfection of siRNAs to knock down Plexin-A1 and Plexin-B3. **G** Proliferation of SFs detected by a Cell Counting Kit-8 (*n* = 3–5). **H** Migration of SFs detected by transwell migration assays (*n* = 3). *P < 0.05; **P < 0.01; ***P < 0.001; ns, not significant. Values of *P* < 0.05 were considered significant. SFs synovial fibroblasts, siRNA small interfering RNA.

Like other cytokines, addition of exogenous Semaphorin 5A promoted the expression of its receptors Plexin-A1 and Plexin-B3 at both the RNA level and the protein level in SFs (Fig. 3A–C). To determine whether Semaphorin 5A really exerts its regulatory function through these receptors, we transfected siRNAs to knockdown Plexin-A1 and Plexin-B3 expression, respectively (Fig. 3D, E). As expected, the effect of Semaphorin 5A on enhancing activation (cytokine secretion, cell proliferation and migration) of SFs was significantly abolished after knockdown either of the two receptors (Fig. 3F–H and Supplementary Fig. 3A–F). Nevertheless, for cell apoptosis, only when Plexin-A1 and Plexin-B3 were simultaneously knockdown, the role of Semaphorin 5A could be abolished (Supplementary Fig. 3G). These suggested that Plexin-A1 and Plexin-B3 all serve as the functional receptors for Semaphorin 5A in RA SFs. Moreover, given that Plexin-B3 expression did not differ significantly between RA SFs and OA SFs, we consider that Plexin-A1 is a more specific receptor for Semaphorin 5A in RA SFs.

### Semaphorin 5A can promote activation of SFs by activating the PI3K/AKT/mTOR signaling pathway

**Fig. 4:**
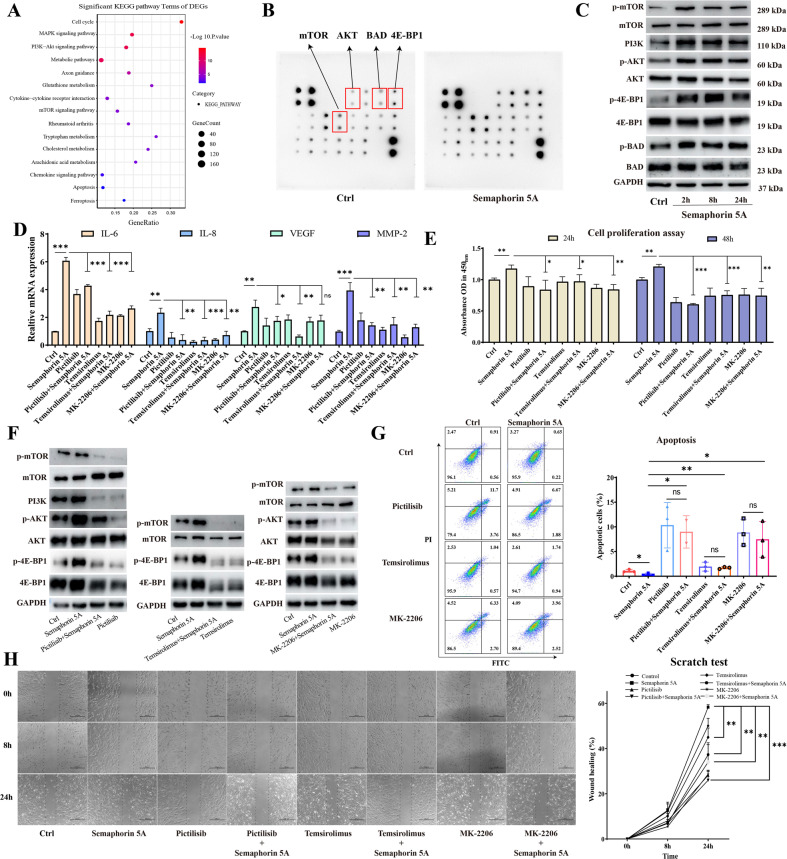
Semaphorin 5A promotes activation of SFs by activating the PI3K/AKT/mTOR signaling pathway. **A** Significant KEGG pathway terms for differentially expressed genes. **B** Original image of the protein array analysis. **C** Total protein and phosphorylated protein levels for the PI3K/AKT/mTOR signaling pathway and its downstream molecules 4E-BP1 and BAD in SFs detected by western blotting after treatment with Semaphorin 5A (1 μg/mL) at different times (*n* = 3). **D** Total protein and phosphorylated protein levels for the PI3K/AKT/mTOR signaling pathway and its downstream molecule 4E-BP1 in SFs detected by western blotting (*n* = 3). **E** mRNA levels of IL-6, IL-8, VEGF, and MMP-2 in SFs detected by qPCR after treatment with pictilisib (10 μM), MK-2206 (5 μM), temsirolimus (100 nM), and Semaphorin 5A (1 μg/mL) (*n* = 3). **F** Proliferation of SFs detected by a Cell Counting Kit-8 (*n* = 3–4). **G** Apoptosis of SFs detected by flow cytometry (*n* = 3). **H** Migration of SFs detected by wound healing assays (*n* = 3). *P < 0.05; **P < 0.01; ***P < 0.001; ns not significant. Values of *P* < 0.05 were considered significant. SFs synovial fibroblasts.

To understand how Semaphorin 5A activated SFs, transcriptome sequencing (Supplementary Fig. 4A–C) and protein array detection (Fig. 4B) were performed on SFs with and without stimulation by exogenous Semaphorin 5A. Some cytokines such as IL-1β, IL-6, IL-8 and MMP-2 and Plexin receptors (Plexin-A1/Plexin-B3) were all up-regulated after Semaphorin 5A stimulation (Supplementary Fig. 4B). The functional and pathway enrichment analyses of differentially expressed genes and the protein microarray results all showed that Semaphorin 5A activated the PI3K/AKT/mTOR signaling pathway (Fig. 4A, B). Subsequently, we demonstrated that Semaphorin 5A did indeed activate this pathway together with phosphorylation of the downstream molecules 4E-BP1 and BAD at different time points (Fig. 4C). These findings suggest that Semaphorin 5A has a persistent activation effect on this pathway. Moreover, we conducted rescue experiments using small-molecule inhibitors of the PI3K/AKT/mTOR signaling pathway. When we added pictilisib (PI3K inhibitor), MK-2206 (AKT inhibitor), and temsirolimus (mTOR inhibitor) to the medium, activation of the pathway and its downstream molecules by Semaphorin 5A was significantly inhibited (Fig. 4D). Meanwhile, the stimulatory effect of Semaphorin 5A on SF activation was significantly abolished, including decreased cytokine secretion (Fig. 4E), decreased cell proliferation (Fig. 4F), increased cell apoptosis (Fig. 4G), and decreased cell migration (Fig. 4H).

### Semaphorin 5A reduces ferroptosis of SFs by GPX4

**Fig. 5:**
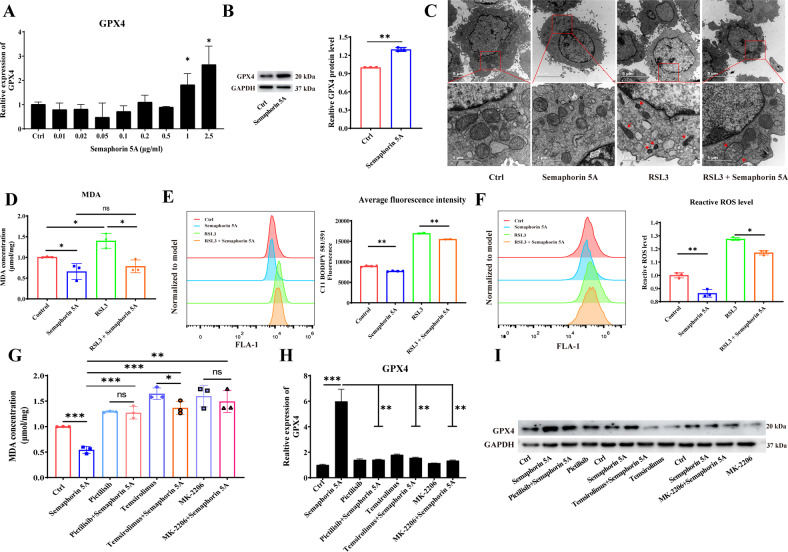
Semaphorin 5A reduces ferroptosis of SFs by GPX4. **A** mRNA levels of GPX4 in SFs detected by qPCR (*n* = 3) after stimulation with different concentrations of Semaphorin 5A. **B** Protein abundances of GPX4 in SFs detected by western blotting (*n* = 3) after stimulation with Semaphorin 5A (1 μg/mL). **C** SF mitochondrial morphology observed by transmission electron microscopy (*n* = 3). **D** Intracellular MDA levels in SFs detected by a Lipid Oxidation (MDA) Assay Kit (*n* = 3). **E** Fluorescence intensity of C11-BODIPY 581/591 in SFs detected by flow cytometry (*n* = 3). **F** ROS levels in SFs detected by flow cytometry (*n* = 3). **G** Intracellular MDA levels in SFs detected by a Lipid Oxidation (MDA) Assay Kit after treatment with pictilisib (10 μM), MK-2206 (5 μM), temsirolimus (100 nM), and Semaphorin 5A (1 μg/mL) (*n* = 3). **H**–**I** mRNA levels and protein abundances of GPX4 in SFs detected by qPCR (**H**, *n* = 3) and western blotting (**I**, *n* = 3) after treatment with pictilisib (10 μM), MK-2206 (5 μM), temsirolimus (100 nM), and Semaphorin 5A (1 μg/mL). *P < 0.05; **P < 0.01; ***P < 0.001; ns, not significant. Values of *P* < 0.05 were considered significant. SFs synovial fibroblasts.

Our gene expression profile analysis and pathway enrichment analysis results indicated that Semaphorin 5A may affect not only the PI3K/AKT/mTOR signaling pathway, but also glutathione metabolism, apoptosis, and ferroptosis (Fig. 4A and Supplementary Fig. 4B). Through a review of the literature, we found that the final outcome of PI3K/AKT/mTOR signaling pathway and glutathione metabolism is cell ferroptosis [17, 18]. Therefore, we hypothesized that Semaphorin 5A can reduce ferroptosis of SFs. Firstly, we investigated the expression of GPX4, a ferroptosis suppressor that prevents the production of lipid peroxidation reaction products [17], in synovial tissues and synovial fibroblasts of RA patients. Unfortunately, there were no significant differences in either protein abundance or mRNA levels compared to OA patients (Supplementary Fig. 5A, B). However, we found that Semaphorin 5A significantly enhanced the RNA level and protein abundance of GPX4 in RA SFs (Fig. 5A, B). When we specifically blocked GPX4 with RSL3, we found that the enhancement of GPX4 by Semaphorin 5A was attenuated (Supplementary Fig. 5C). In addition, RSL3 significantly reduced the migration and proliferation of SFs, and these effects were partially alleviated by the addition of exogenous Semaphorin 5A (Supplementary Fig. 5D, E). Next, we observed that SF mitochondria became smaller, the membrane density increased, and the cristae decreased after treatment with RSL3 alone by transmission electron microscopy (Fig. 5C). These are the typical electron microscopic characteristics of ferroptosis. Interestingly, when the cells were co-treated with Semaphorin 5A and RSL3, the changes in mitochondrial morphology were significantly improved and most of the mitochondria remained in their normal form (Fig. 5C). Changes in intracellular lipid peroxidation and ROS are other characteristics of ferroptosis. The levels of intracellular lipid peroxidation and ROS in SFs were significantly increased after treatment with RSL3 alone, but were significantly suppressed by Semaphorin 5A. Furthermore, Semaphorin 5A significantly reduced the levels of RSL3-induced lipid peroxidation and ROS (Fig. 5D–F). These findings indicate that Semaphorin 5A reduces ferroptosis of SFs by increasing the intracellular GPX4 level.

### Semaphorin 5A reduces ferroptosis of SFs by activating the PI3K/AKT/mTOR/SREBP1/SCD-1 axis

**Fig. 6:**
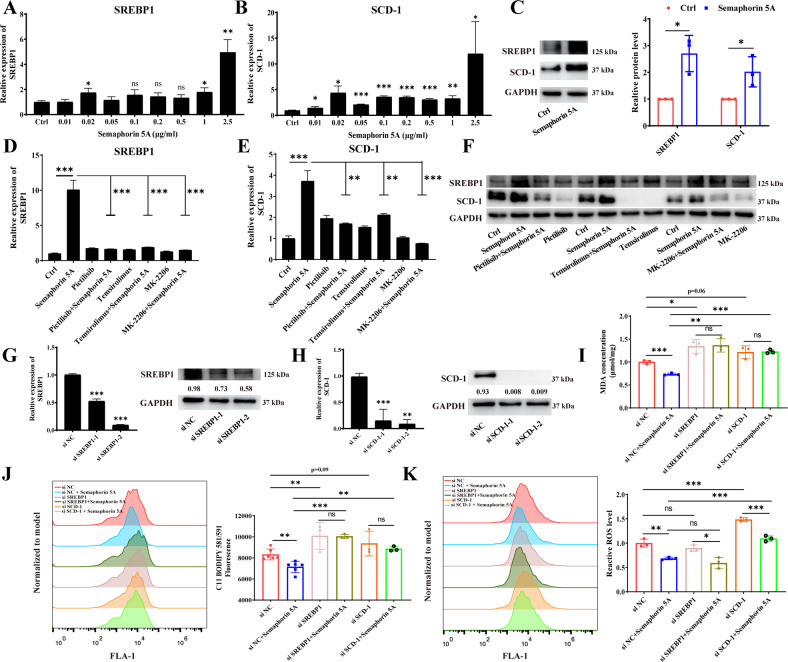
Inhibitory effect of Semaphorin 5A on ferroptosis is abolished by inhibition of SREBP1/SCD-1 signaling. **A**–**C** mRNA levels and protein abundances of SREBP1 and SCD-1 in SFs detected by qPCR (**A**, **B**, *n* = 3) and western blotting (**C**, *n* = 3) after treatment with different concentrations of Semaphorin 5A. **D**–**F** mRNA levels and protein abundances of SREBP1 and SCD-1 in SFs detected by qPCR (**D**, **E**, *n* = 3) and western blotting (**F**, *n* = 3) after treatment with pictilisib (10 μM), MK-2206 (5 μM), temsirolimus (100 nM), and Semaphorin 5A (1 μg/mL). **G**, **H** RNA levels and protein abundances of SREBP1 and SCD-1 in SFs were detected by qPCR (*n* = 3) and western blotting (*n* = 3) after transfection of siRNAs to knock down SREBP1 (**G**) and SCD-1 (**H**). **I** Intracellular MDA levels of SFs detected by a Lipid Oxidation (MDA) Assay Kit (*n* = 3). **J** Fluorescence intensity of C11-BODIPY 581/591 in SFs detected by flow cytometry (*n* = 3/6). **K** ROS levels in SFs detected by flow cytometry (*n* = 3). *P < 0.05; **P < 0.01; ***P < 0.001; ns, not significant. Values of *P* < 0.05 were considered significant. SFs synovial fibroblasts.

Recently, several studies have confirmed a close relationship between mTORC1 signaling and ferroptosis [18, 19]. Therefore, we hypothesized that Semaphorin 5A reduces ferroptosis of SFs by activating the PI3K/AKT/mTOR signaling pathway. First, MDA levels were measured to reflect intracellular lipid peroxidation. The intracellular MDA levels were significantly increased by the addition of pathway inhibitors alone, but were significantly reduced by Semaphorin 5A alone. We further found that the reduction in MDA levels by Semaphorin 5A was significantly abolished by the addition of the pathway inhibitors (Fig. 5G). Interestingly, the RNA level and protein abundance of GPX4 in SFs enhanced by exogenous Semaphorin 5A were significantly suppressed when PI3K/AKT/mTOR signaling was inhibited (Fig. 5H–I). These results suggest that Semaphorin 5A enhances GPX4 expression by activating the PI3K/AKT/mTOR signaling pathway, thereby suppressing ferroptosis of SFs. Yi et al. [18] reported that activation of PI3K/AKT/mTOR signaling protects cancer cells against ferroptosis through SREBP1/SCD-1-mediated lipogenesis. Therefore, we explored whether this signaling pathway was involved in the Semaphorin 5A-mediated reduction of SF ferroptosis. As expected, Semaphorin 5A significantly enhanced the RNA levels and protein abundances of SREBP1 and SCD-1 (Fig. 6A–C), and these phenomena were abolished by specific inhibitors of the PI3K/AKT/mTOR signaling pathway (Fig. 6D–F). Meanwhile, we also detected the expression of SREBP1 and SCD-1 in synovial fibroblasts to understand the basic situation of lipid synthesis pathway in RA patients. Compared with OA SFs, SCD-1 levels were significantly increased in RA SFs, while SREBP1 had no significant difference (Supplementary Fig. 6A–C). This is consistent with the conclusion that RA SFs underwent metabolic reprogramming, at least in the activation of lipid synthesis signals [10, 20]. Subsequently, we successfully knocked down SREBP1/SCD-1 by siRNA transfection and observed the effect on ferroptosis of SFs (Fig. 6G, H). After knockdown of SCD-1, the intracellular lipid peroxidation level and ROS level were significantly increased, while only the lipid peroxidation level was significantly enhanced after knockdown of SREBP1. We further found that the reduction in lipid peroxidation level by Semaphorin 5A was significantly abolished by knockdown of SREBP1 and SCD-1, while there was no effect on the ROS level (Fig. 6I–K). These observations further demonstrate the specificity of SREBP1/SCD-1 signaling for ferroptosis. In summary, our results confirmed that Semaphorin 5A enhances not only GPX4 expression but also SREBP1/SCD-1 signaling by activating the PI3K/AKT/mTOR signaling pathway, thereby suppressing ferroptosis of SFs.

## Discussion

The joint inflammation associated with RA results in an abundance of cytokines and immune cell infiltration in the interarticular space. Abnormal activation of SFs plays an important role in this process, the mechanism of which remains unknown [21]. It is of great clinical significance to identify factors that influence the abnormal activation of RA SFs and develop new therapeutic strategies.

Semaphorins play important roles in regulating various responses of the immune system. Our previous study showed that Semaphorin 5A, a member of the Semaphorin family, was elevated in the serum of RA patients [8]. Recently, Xiao et al. [9] reported that Semaphorin 5A was also elevated in the synovial membrane, synovial fluid, and SFs of RA patients. These findings are consistent with our results in synovial fluid and synovial tissue from RA patients. Meanwhile, we also found a significant positive correlation between the level of Semaphorin 5A in synovial fluid and the degree of disease activity in RA patients, suggesting that Semaphorin 5A may be involved in the disease progression and activity of RA patients. However, our results showed that compared with OA, the expression of Semaphorin 5A was significantly increased in SMs from synovial tissues of RA patients, but only slightly increased in SFs without significant difference. These findings suggest that the elevated Semaphorin 5A in synovial fluid may be mainly derived from SMs, with a small part from SFs. Although a pathophysiological significance of the increased Semaphorin 5A in SFs cannot be excluded, the source of the elevated Semaphorin 5A in synovial fluid is likely to be SMs.

SFs have tumor cell-like phenotypes and are functionally altered in many ways [2, 21]. Xiao et al. [9] found that Semaphorin 5A promotes pannus formation, which reflects only a small part of the functional changes in SFs. More comprehensive and systematic studies are needed. In our study, we found that stimulation with exogenous Semaphorin 5A promoted the expression of not only angiogenic factors, but also various inflammatory cytokines and osteoclast-related factors. Joint inflammation and cartilage destruction are important causes of pain and joint deformity in RA patients. Moreover, Semaphorin 5A significantly enhanced other functions of SFs, such as cell proliferation, migration, and invasion. Importantly, Semaphorin 5A significantly reduced SF apoptosis, which is the basis for abnormal activation of RA SFs [22]. The increased survival of SFs further enhances its inflammatory, invasive, and other aspects, thereby enhancing the destructive inflammatory and immune microenvironment. Previous studies identified Plexin-A1 and Plexin-B3 as the functional receptors for Semaphorin 5A [5, 14, 15]. However, the receptor bound by Semaphorin 5A in RA SFs has not been reported in detail. Our results also indicate that SM-derived Semaphorin 5A plays an important role in abnormal activation of SFs by binding to Plexin-A1 and Plexin-B3 on SFs. Considering that the Plexin-A1 level was much higher in RA SFs compared with OA SFs, Semaphorin 5A and Plexin-A1 co-located on the membrane of SFs, we predicted that Plexin-A1 may be a more specific receptor for Semaphorin 5A in RA SFs. This is further evidence of a mutual crosstalk between SMs and SFs in the synovial microenvironment.

**Fig. 7:**
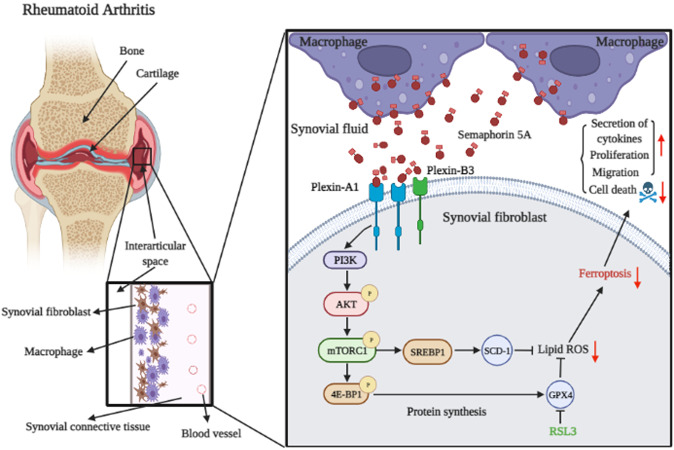
Mechanism for Semaphorin 5A action on abnormal activation of RA SFs. SMs secrete a large amount of Semaphorin 5A, leading to an increased level of Semaphorin 5A in the synovial fluid. Semaphorin 5A then binds to Plexin-A1 and Plexin-B3 on SFs and activates the PI3K/AKT/mTOR signaling pathway. Activated mTORC1 signaling inhibits lipid peroxidation, ROS production, and ferroptosis by increasing the phosphorylation of 4E-BP1, leading to increased protein synthesis of GPX4, and also by activating downstream SREBP1/SCD-1 signaling. Reduced ferroptosis leads to increased survival of SFs, further promoting inflammation and immunity. SFs synovial fibroblasts, SM synovial macrophages.

To determine the exact molecular mechanism, we performed transcriptome sequencing and protein array analysis. Consistent with previous studies, we found that Semaphorin 5A mainly affected the PI3K/AKT/mTOR signaling pathway and its downstream molecule activation in SFs [23, 24]. However, we found that besides apoptosis, Semaphorin 5A activates SFs through inhibition of ferroptosis. A previous study demonstrated that GSH-based GPX4 inactivation is the most important mechanism of ferroptosis, because GPX4 is the only glutathione peroxidase in the cells for liposome peroxidase reduction [17]. We found that Semaphorin 5A inhibited ferroptosis in SFs by significantly promoting GPX4 expression. Interestingly, Semaphorin 5A still significantly inhibited ferroptosis even after treatment with RSL3, a specific inhibitor of GPX4. We further explored the relationship between the PI3K/AKT/mTOR signaling pathway and ferroptosis. Zhang et al. [19] reported that this pathway promotes GPX4 protein translation by inhibiting the downstream molecule 4E-BP1, thereby inhibiting ferroptosis. Another study by Yi et al. [18] indicated that this pathway also inhibits ferroptosis through the SREBP1-SCD-1 axis. Although these findings were obtained in tumor cells, SFs have tumor cell-like properties and we consider that there may be a similar association. As expected, Semaphorin 5A enhanced not only GPX4 expression but also SREBP1/SCD-1 signaling by activating the PI3K/AKT/mTOR signaling pathway, thereby suppressing ferroptosis of SFs (Fig. 7). These in vitro findings are exciting and we will be conducting animal studies in the future to verify our findings in vivo.

Semaphorin 5A has been described to promote angiogenesis by increasing endothelial cell proliferation and decreasing apoptosis [23]. Our study demonstrated that Semaphorin 5A not only affect apoptosis but also ferroptosis in RA. Recently, during preparation of this manuscript, Wu et al. published new evidence supporting the role of ferroptosis in RA. They found that TNF-α antagonists combined with ferroptosis inducers enhanced ferroptosis in fibroblasts and improved arthritis progression in collagen-induced arthritis mice [25]. These suggest targeting ferroptosis may be a new direction in the development of RA therapy. In addition, ferroptosis induction attenuates the interaction between fibroblasts and immune cells, which helps reduce inflammation and restore synovial homeostasis [25]. Although with the advent of biologic agents targeting cytokines such as TNF-α and IL-6, disease progression in most RA patients is controlled, some patients do not respond well and develop resistance [1]. This is most likely due to ferroptosis resistance in RA patients. Thus, besides cell apoptosis, targeting ferroptosis may be a potential therapeutic approach and direction for the future treatment of these patients.

In the interarticular space, there are a variety of macrophages, including synovial lining layer macrophages, interstitial macrophages, and host tissue macrophages [26]. Semaphorin 5A was found to be mainly derived from CD68^+^ macrophages for the first time in our study, but the specific subgroup was not explored. A variety of emerging experimental devices and techniques, such as three-dimensional light-sheet fluorescence microscopy and single-cell sequencing technology, have provided new possibilities for dynamic origin tracing and more accurate classification of cell subsets [27]. More in-depth exploration and research are needed in the future. Meanwhile, Gras et al. [7] reported that Semaphorin 5A was cleaved by ADAM17 on the cell membrane, resulting in an elevated level of soluble Semaphorin 5A. Whether Semaphorin 5A on the surface of the SM membrane is cleaved by ADAM17 or other proteolytic enzymes also warrants further exploration. Finally, due to the apparent crosstalk between different programmed cell deaths, their effects may be different in different stages of RA [28, 29]. Therefore, although we found that Semaphorin 5A inhibited both ferroptosis and apoptosis, more studies are needed to understand the mechanisms associated with crosstalk of apoptosis and ferroptosis in RA and the role of Semaphorin 5A in the different stages of RA.

In the present study, we found that elevated Semaphorin 5A in synovial fluid of RA patients is mainly derived from CD68^+^ SMs rather than Vimentin^+^ SFs. Furthermore, Semaphorin 5A can bind to Plexin-A1 and Plexin-B3 on RA SFs to induce their activation. More importantly, we found that Semaphorin 5A promotes SF activation by inhibiting ferroptosis of SFs. Mechanistically, Semaphorin 5A enhances GPX4 expression and SREBP1/SCD-1 signaling via the PI3K/AKT/mTOR signaling pathway, thereby suppressing ferroptosis of SFs. Targeting Semaphorin 5A and ferroptosis could be an effective and valuable therapeutic strategy for patients with RA.

## Materials and methods

Details of the cell isolation and culture, immunohistochemistry and immunofluorescence, ELISA, real-time quantitative PCR (qPCR), small interfering RNA (siRNA) transfection, cell apoptosis, cell proliferation assay, wound healing assay, transwell migration assay, RNA sequencing, protein array analysis and Western blot are provided in the Supplementary Materials.

### Patient samples and informed consent

Synovial fluid and synovial tissue samples were collected from patients with clinically diagnosed RA, osteoarthritis (OA), healthy control (HC) subjects at the Second Affiliated Hospital of Zhejiang University School of Medicine from June 2020 to October 2021. Ethical approval was obtained from the Ethics Committee of the Second Affiliated Hospital of Zhejiang University School of Medicine, Hangzhou, China (approval number: 2020-306). All patients signed an informed consent form. The RA patients met the American College of Rheumatology 1987 diagnostic criteria and the American College of Rheumatology/European League Against Rheumatism 2010 diagnostic criteria for RA [30, 31]. Clinical and laboratory features in patients with RA and OA and HC subjects were shown in Supplementary Table 1 (synovia) and Table 3 (synovial tissue samples), respectively.

### Transmission electron microscopy

After treatment with 1 μg/mL Semaphorin 5A (Q13591, novoprotein) and/or 150 nM RSL3 (HY-100218A; MCE), approximately (2–6) × 10^6^ cells were collected in a 1.5-mL eppendorf tube and fixed with 1 mL of 2.5% Glutaraldehyde Fixed Solution (PH9003; PHYGENE). After rinsing with PBS, the samples were fixed with 50 μL of 1% osmium for 1 h and then fixed/stained with 100 μL of 2% uranium acetate aqueous solution for 30 min. Next, the samples were successively dehydrated through a series of different ethanol concentrations and 100% acetone. After embedding, ultrathin sections were cut, stained, and observed under a transmission electron microscope (Talos F200C 200kv, Thermo Scientific).

### Detection of lipid peroxidation level

Malondialdehyde (MDA), a product of membrane lipid peroxidation, shows a positive correlation with ferroptosis. A Lipid Oxidation (MDA) Assay Kit (S0131S; Beyotime) was used to detect MDA levels in SFs. Briefly, the cells were lysed using Lysis Solution (P0013; Beyotime) and centrifuged. The supernatant was collected and the protein concentration was detected using a BCA Protein Assay Kit (P0010; Beyotime). After preparing the MDA working solution in accordance with the kit instructions, a sample aliquot or blank control was added for determination. The test solution was boiled for 15 min, cooled to room temperature, and centrifuged to obtain the supernatant. Finally, the absorbance was measured at 532 nm using a multimode reader (Spark Cyto, TECAN).

C11-BODIPY 581/591 is a lipid-soluble fluorescent probe used to indicate lipid peroxidation in living cells. According to the manufacturer’s instructions, an appropriate volume of C11-BODIPY 581/591 (RM02821, ABclonal) was added to the cells and incubated for 30 min. After removal of the excess dye by washing with PBS, a cell pellet was obtained by 0.25% trypsin digestion and centrifugation. The cells were then resuspended in PBS containing 5% fetal bovine serum (FBS) and detected by flow cytometry (CytoFLEX LX, Beckman Coulter).

### Treatment with small-molecule inhibitor

Cells were treated with small-molecule inhibitors of PI3K-AKT-mTOR signaling to detect the function of Semaphorin 5A after inhibition of this pathway. They are PI3K inhibitor, pictilisib (GDC-0941, HY-50094; MCE) 10 μM, AKT inhibitor, MK-2206 (HY-108232; MCE) 5 μM, and mTOR inhibitor, temsirolimus (CCI-779, HY-50910; MCE) 100 nM.

### Detection of reactive oxygen species (ROS) level

Intracellular ROS levels were measured with a Reactive Oxygen Species Assay Kit (S0033S; Beyotime). Briefly, cells were stimulated with a ROS positive control (Rosup) or Semaphorin 5A for 24 h and then loaded with DCFH-DA as a probe. Finally, the cells were detected by flow cytometry (CytoFLEX LX, Beckman Coulter).

### Statistical analysis

IBM SPSS Statistics 25 (IBM Corp.) and GraphPad Prism 8.0 (GraphPad Software Inc.) were used to analyze the data and draw scatter diagrams. For normally distributed data expressed as mean values ± SD (standard deviation), the differences between groups were analyzed by Student’s t test. For nonparametric data, results were expressed as median (range) values, and the differences between groups were analyzed by the Mann-Whitney U test. Spearman’s correlation coefficient was applied to detect the correlation between two groups. *P* values less than 0.05 were considered significant. The number of independent technical repeats (n) are indicated in figure legends.

## Supplementary information

Supplementary Information

Supplementary table-RNA-seq

Original Data File

aj-checklis

## Data Availability

The data used to support the findings of this study are available from the corresponding author upon request.

## Materials availability

The materials used to support the findings of this study are available from the corresponding author upon request.
