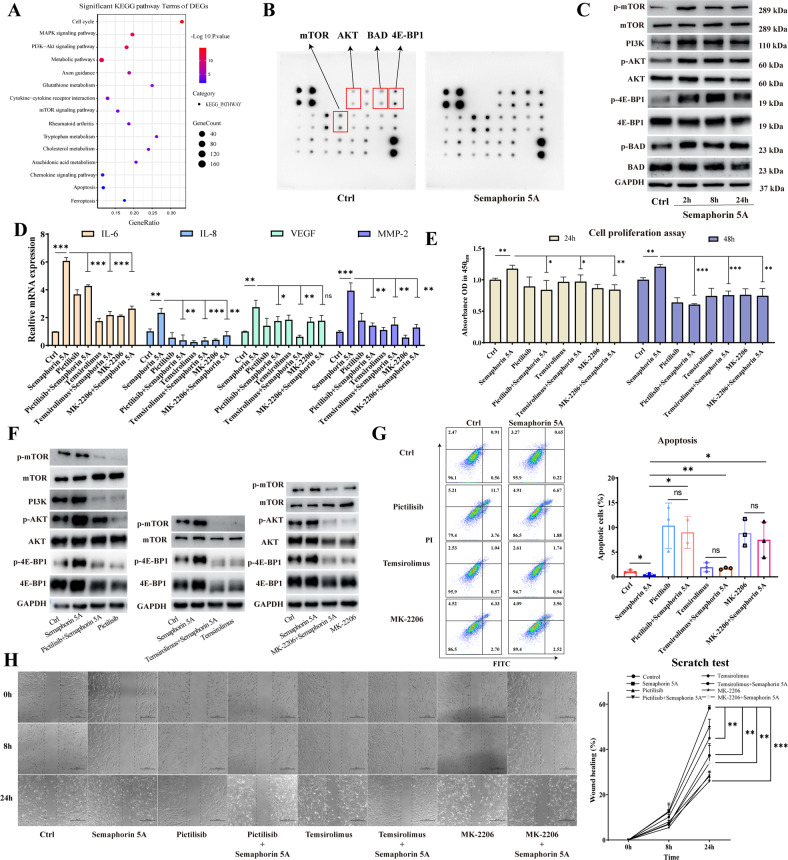# Correction: Semaphorin 5A suppresses ferroptosis through activation of PI3K-AKT-mTOR signaling in rheumatoid arthritis

**DOI:** 10.1038/s41419-023-05697-0

**Published:** 2023-03-14

**Authors:** Qi Cheng, Mo Chen, Mengdan Liu, Xin Chen, Lingjiang Zhu, Jieying Xu, Jing Xue, Huaxiang Wu, Yan Du

**Affiliations:** 1 Department of Rheumatology, The Second Affiliated Hospital of Zhejiang University School of Medicine, 88 Jiefang Road, 310009 Hangzhou, China; 2 Department of Clinic Medicine, The Second Affiliated Hospital of Zhejiang University School of Medicine, 88 Jiefang Road, 310009 Hangzhou, China; 3 Department of Neurology, Linping District Hospital of Integrated Traditional Chinese and Western Medicine, 311199 Hangzhou, Zhejiang China

**Keywords:** Autoimmunity, Cell death and immune response, Signal transduction, Inflammation

Correction to: *Cell Death and Disease* 10.1038/s41419-022-05065-4, published online 14 July 2022

The original version of this article contained a mistake in figure 4. There was an error in figure 4H. In these wound healing assays, the figure in group “Pictilisib + Semaphorin 5A” was mistakenly repeated with that in group “MK-2206+Semaphorin 5A” at 24h! The authors apologize for this mistake. There is no impact on the final conclusions. The original article has been corrected.