# Supplementary material for: Semaphorin 5A suppresses ferroptosis through activation of PI3K-AKT-mTOR signaling in rheumatoid arthritis
Source: Cell Death Dis. 2022 Jul 14;13(7):608. doi: 10.1038/s41419-022-05065-4 (PMC9283415; doi:10.1038/s41419-022-05065-4)
Supplement: Supplementary file 1 — Supplementary Information [file 41419_2022_5065_MOESM1_ESM.docx]

**Supplementary Information**

**Semaphorin 5A suppresses ferroptosis through activation of PI3K-AKT-mTOR signaling in rheumatoid arthritis**

Qi Cheng^1,2^, Mo Chen^1^, Mengdan Liu^1^, Xin Chen^1,2^, Lingjiang Zhu^1^, Jieying Xu^1,3^, Jing Xue^1^, Huaxiang Wu^1*†^, Yan Du^1*†^

^1^Department of Rheumatology, The Second Affiliated Hospital of Zhejiang University School of Medicine, 88 Jiefang Road, Hangzhou, 310009, China

^2^Department of Clinic Medicine, The Second Affiliated Hospital of Zhejiang University School of Medicine, 88 Jiefang Road, Hangzhou, 310009, China

^3^Department of Neurology, Linping District Hospital of Integrated Traditional Chinese and Western Medicine, Hangzhou, 311199, Zhejiang, China

^*^Correspondence

Corresponding Author

Yan Du (duyan2014@zju.edu.cn) or Huaxiang Wu ([wuhx8855@zju.edu.cn](mailto:wuhx8855@zju.edu.cn)).

^†^These authors contributed equally to this work.

**Materials and Methods**

**Cell isolation and culture**

Surgically resected synovial tissue specimens were transported to a laboratory for cell separation. First, each tissue specimen was washed as follows: 1) one rinse with 75% alcohol; 2) three rinses with sterile phosphate-buffered saline (PBS); 3) one wash with serum-free Dulbecco’s modified Eagle’s medium (DMEM). Next, the tissue was cut into small pieces and digested with 2 mg/mL type IV collagenase (17104019; Thermo Scientific) in DMEM. The obtained cell suspension was filtered through a 100-μm cell filter and centrifuged at 1500 rpm for 5 min. The cell pellet was suspended and washed twice with serum-free DMEM. The cells were then cultured in DMEM which containing 10% fetal calf serum, 100 IU/mL penicillin, and 100 μg/mL streptomycin in an incubator at 37°C under 5% CO_2_. The medium was changed every 2 days, and the third-to-tenth generations of cells were used for experiments. A human RA fibroblast cell line, MH7A, was purchased from Wuhan Fine Biotech Co. Ltd. with a cell-line STR Authentication report and the cells were cultured under the same conditions as the primary cells.

**Immunohistochemistry and immunofluorescence**

After sample collection, each tissue specimen was quickly and thoroughly fixed in 4% paraformaldehyde (P1110; Solarbio) or frozen at −80°C. After the tissue was dehydrated and made transparent, paraffin (preheated to 60°C) was added and left overnight. Subsequently, the fixed or frozen tissues were sectioned using a microtome and the paraffin-fixed tissues were subjected to antigen retrieval. The tissue sections were treated with 5% bovine serum albumin (BSA) and hydrogen peroxide to block endogenous enzyme activity, washed, and incubated with a primary antibody working solution overnight at 4℃. For immunohistochemistry, the sections were incubated with an appropriate amount of biotin-labeled secondary antibody for 30 min at room temperature. After adding the DBA chromogenic agent for 5–10 min, the sections were rinsed, redyeing, dehydrated, made transparent, sealed, and observed under a forward microscope (Leica DM3000LED). For tissue immunofluorescence, the sections were incubated with an appropriate amount of fluorescein-conjugated secondary antibody for 1 h at room temperature. After rinsing with PBS, the sections were redyeing, sealed, and observed with a forward fluorescence microscope (Leica DM6B). For cell immunofluorescence, the cell slides were fixed with 4% paraformaldehyde (P1110; Solarbio) and sealed with 5% BSA. After incubation with corresponding primary and secondary antibodies, the cells were observed under a forward fluorescence microscope (Leica DM6B). The antibodies used for immunostaining were anti-Semaphorin 5A (PA5-47791; Thermo Scientific), anti-Plexin-A1 (MAB6536; R&D Systems), Alexa Fluor® 488 anti-Vimentin Antibody (677809, Biolegend), Alexa Fluor® 488 anti-human CD68 Antibody (333811, Biolegend) and Donkey Anti-Sheep IgG NorthernLights™ NL557-conjugated Antibody (NL010; R&D Systems).

**Enzyme-linked immunosorbent assay**

The levels of Semaphorin 5A in peripheral blood and synovial fluid samples were measured using a Semaphorin 5A BioAssay™ ELISA Kit (153105-Z; United States Biological) in accordance with the manufacturer’s instructions.

**RNA extraction and** **real-time qPCR**

Total RNA was extracted from synovial tissues or cells using Trizol reagent (Ambion, Thermo Scientific). PrimeScript™ RT reagent Kit (RR047A, TaKaRa) and qPCR regent (RR820A, TaKaRa) were used for genomic removal of total DNA and reverse transcription. The qPCR analysis was performed using a 7500 Fast Real-Time PCR system (Applied Biosystems, Thermo Scientific) and a TB Green PCR protocol. The sequences of the primers for the target genes are shown in Supplementary Table 4. Gene expression was calculated by the ΔΔCt method. GAPDH were used as an internal control.

**Small interfering RNA transfection**

For experiments involving siRNAs (GenePharma), appropriate cells were seeded in 6-well plates and transfected using the GP-Transfect-Mate reagent (GenePharma). Levels of mRNA expression were detected at 24 h and levels of protein expression were detected at 48 h. The sequences of the siRNAs were shown in Supplementary Table 5.

**Cell apoptosis**

Cells in 6-well plates were digested with 0.25% trypsin (BC-CE-005; Bio-Channel) and centrifuged to collect the cell pellets. Staurosporine (HY-15141; MCE) 100 nM was used to induce apoptosis. Cell apoptosis was detected by Annexin V-FITC Apoptosis Detection Kit (C1062S; Beyotime). After cell suspension, 195 μL of annexin V-FITC binding solution was added. Next, 5 μL of annexin V-FITC and 10 μL of propyl iodide were added successively and incubated at room temperature (20°C–25℃) for 10–20 min in the dark. Finally, apoptotic cells were detected using a flow cytometer (CytoFLEX LX, Beckman Coulter). Data analysis was performed using FlowJo V10 (BD biosciences).

**Cell proliferation**

Cell proliferation was detected by a Cell Counting Kit-8 (Dojindo Molecular Technologies) in accordance with the manufacturer’s instructions. Cells (5 × 10^3^) were seeded into the wells of a 96-well plate and evaluated at 24 h after cell adherence. Following addition of 10 μL of CCK-8 solution to each well, the absorbance was measured at 450 nm with a microplate reader (ELX-808, BioTek).

**Wound healing assay**

Appropriate cells were seeded into 12-well plates and cultured for 24 h. When they grow to 100%, a 200-µL pipette tip was used to create a scratch that was perpendicular to the cell plane. After three rinses with sterile PBS and addition of fresh DMEM containing 1% FBS, the cells were cultured in an incubator at 37℃ under 5% CO_2_. The cells were taken out of the incubator at 0, 8, and 24 h and observed under an inverted microscope (Leica DMIL) to measure the width of the scratch.

**Transwell migration assay**

For the transwell migration assay, 200 μL of serum-free DMEM containing 3 × 10^4^ cells was added into the upper chamber, while 500 μL of DMEM containing 10% FBS and 1 μg/mL Semaphorin 5A was added into the lower chamber of the transwell plate (#3378; Corning). After 24 h of culture, the chamber was removed and a cotton swab was used to gently wipe the cells in the upper chamber. Next, 0.1% crystal violet solution (G1063; Solarbio) was added to stain the cells for 10 min. After air drying, the cells were observed and counted in five fields under a forward fluorescence microscope (Leica DM2500).

**RNA sequencing**

Total RNA was extracted from three control samples and three treated samples using Trizol reagent. After quality assessment, 1 μg of total RNA was used for library preparation. Reverse transcription and PCR were performed on the RNA. Subsequently, libraries with different indices were multiplexed and loaded onto an Illumina HiSeq/Novaseq instrument (Illumina) or MGI2000 instrument (MGI) in accordance with each manufacturer’s instructions. After quality control and mapping, differentially expressed genes were identified using the DESeq2 Bioconductor package. Two online tools, DAVID v 6.8 (<https://david.ncifcrf.gov/home.jsp>) and KOBAS3.0 (<http://kobas.cbi.pku.edu.cn/kobas3>), were used for GO and KEGG enrichment analyses, respectively. Values of *P* < 0.05 were considered significant. Bubble diagrams were created using the R language ggplot2 package.

**Protein array analysis**

Protein sample from RA SF cell lysates were assayed using a RayBio® Human Phosphorylation Pathway Profiling Array C55 (AAH-PPP-1-8; RayBiotech Inc.) and processed in accordance with the manufacturer’s instructions. The steps were as follows: (1) blocking, 2 mL of Blocking Buffer was pipetted into each well and incubated for 30 min at room temperature; (2) sample incubation, 1 mL of diluted or undiluted sample was pipetted into each well and incubated for 2.5–5 h at room temperature; (3) first wash, 2 mL of 1× Wash Buffer I was pipetted into each well and incubated for 5 minutes at room temperature, repeated twice; (4) primary antibody incubation, 1 mL of prepared Detection Antibody Cocktail was pipetted into each well and incubated for 1.5–2 h at room temperature; (5) second wash, as described for first wash; (6) secondary antibody incubation, 1 mL of 1× HRP-Anti-Rabbit IgG was pipetted into each well and incubated for 2 h at room temperature or overnight at 4°C; (7) third wash, as described for first wash; (8) chemiluminescence detection.

**Western blot analysis**

After tissue grinding or cell lysis and centrifugation, the protein concentrations in the supernatants were measured with the BCA Protein Assay Kit. After preparation of the required protein concentration, the samples were electrophoresed in an appropriate separation gel under 200-V constant voltage and transferred onto a polyvinylidene fluoride membrane (Immobilon-P; Millipore). The membrane was blocked with 5% skim milk in a shaker for 1 h at room temperature, and incubated with a primary antibody overnight at 4°C. The membrane was then washed with Tris-buffered saline containing 1% Tween-20 and incubated with an appropriate secondary antibody for 1 h at room temperature. The blots were established by an increased chemiluminescence detection kit (Pierce Biotechnology, Inc., Rockford, IL, USA). GAPDH was used as the internal control. The following primary antibodies were used: anti-GAPDH (60004-1-Ig; Proteintech), anti-Semaphorin 5A (AP2712b; Abcepta), anti-Plexin-A1 (MAB6536; R&D Systems), anti-Plexin-B3 (AF4958; R&D Systems), anti-PI3K (67071-1-Ig; Proteintech), anti-AKT (#4691; CST), anti-p-AKT (#4060; CST), anti-mTOR (#2972; CST), anti-p-mTOR (#2971; CST), anti-BAD (#9292; CST), anti-p-BAD (#9291; CST), anti-4E-BP1 (ab32024; Abcam), anti-p-4E-BP1 (abs113154; Absin), anti-GPX4 (67763-1-Ig; Proteintech), anti-SREBP1 (66875-1-Ig; Proteintech), and anti-SCD-1 (ab236868; Abcam).

**Supplementary Figures**

**
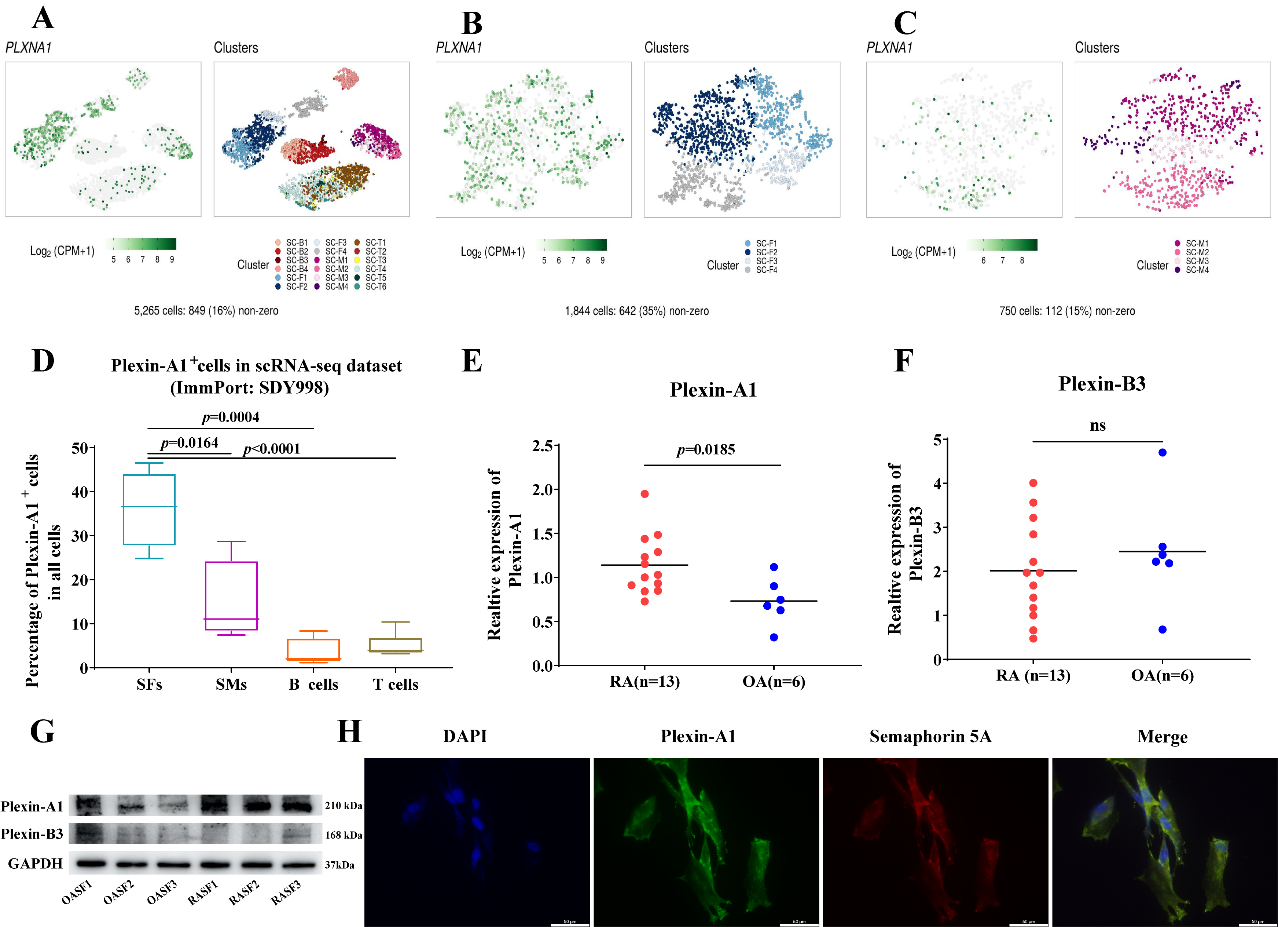
**

**Supplementary Figure 1.** Expression of Plexin-A1 and Plexin-B3 in SMs and SFs. **A**, Plexin-A1-positive cells (*n* = 849) among all cells (*n* = 5,265). **B**, Plexin-A1-positive cells (*n* = 642) in four fibroblast subsets (*n* = 1 844). **C**, Plexin-A1-positive cells (*n* = 112) in four monocyte subsets (*n* = 750). **D**, Plexin-A1-positive cells in SFs, SMs, B cells, and T cells (Single-cell RNA-seq dataset, ImmPort: SDY998). **E–F**, mRNA levels of Plexin-A1 (E) and Plexin-B3 (F) were detected by PCR in SFs from 13 RA patients and 6 OA patients. G, protein abundance of Plexin-A1and Plexin-B3 detected by Western blot in 3 RASFs and 3 OASFs. H, Representative fluorescence image of colocalization of Semaphorin 5A and Plexin-A1 on the membrane of SFs was observed by a forward fluorescence microscope (*n* = 5). ns, not significant. Values of *P* < 0.05 were considered significant. RA, rheumatoid arthritis; OA, osteoarthritis; SFs, synovial fibroblasts; SMs, synovial macrophages.


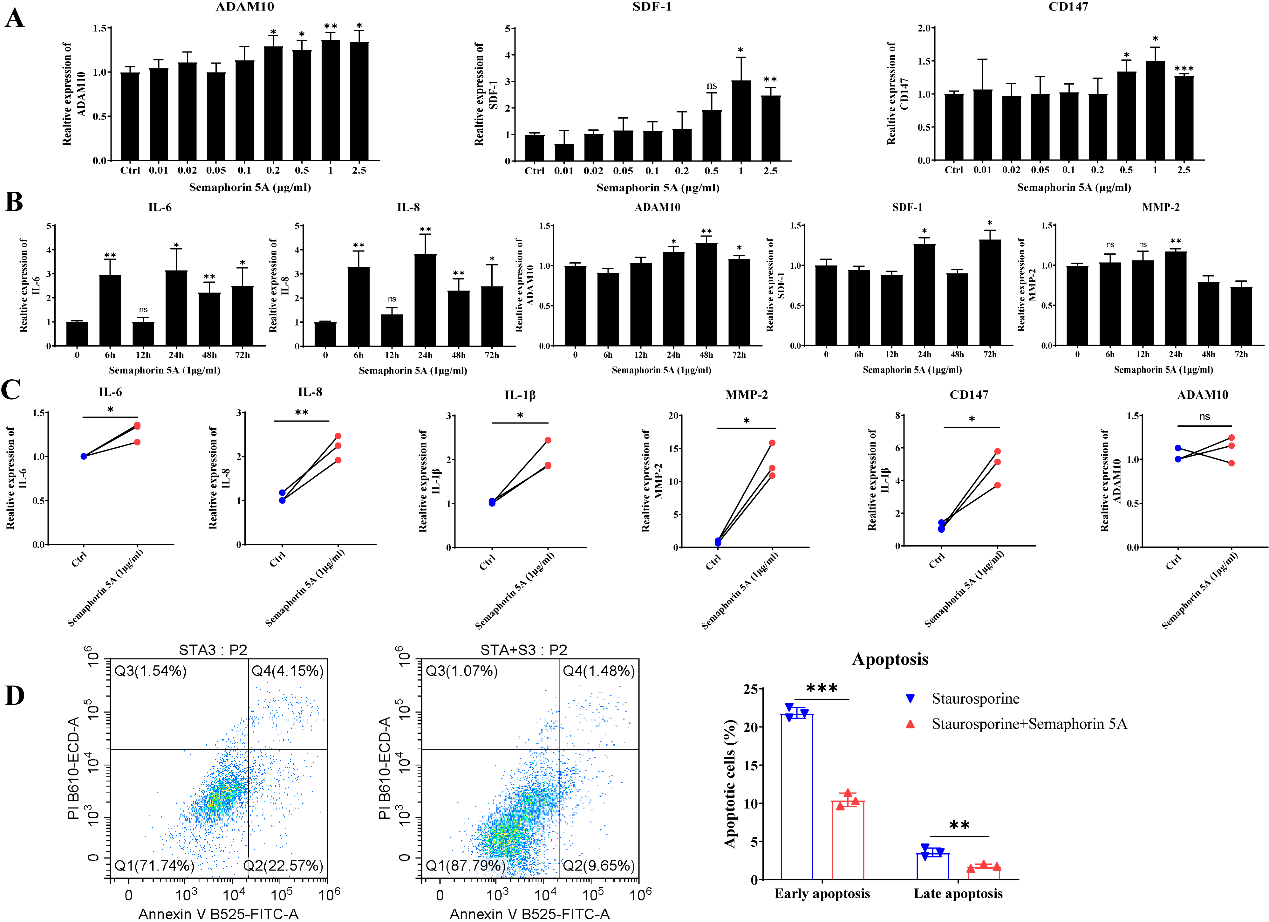


**Supplementary Figure 2.** Semaphorin 5A promotes secretion of cytokines in not only MH7A cells but also primary SFs. **A**, mRNA levels of ADAM10, SDF-1, and CD147 in SFs detected by qPCR (*n* = 3) after treatment with different concentrations of Semaphorin 5A. **B**, mRNA levels of IL-6, IL-8, ADAM10, SDF-1, and MMP-2 in SFs detected by qPCR (*n* = 3) after treatment with Semaphorin 5A (1 μg/mL) at different times. **C**, mRNA levels of IL-6, IL-8, IL-1β, MMP-2, CD147, and ADAM10 in primary SFs detected by qPCR (*n* = 3) after treatment with Semaphorin 5A (1 μg/mL). D, Apoptosis of SFs detected by flow cytometry (*n* = 3) after treatment with Staurosporine (100 nM) alone or co-treatment with Semaphorin 5A (1 μg/mL). **P* < 0.05; ***P* < 0.01; ****P* < 0.001; ns, not significant. Values of *P* < 0.05 were considered significant. SFs, synovial fibroblasts.


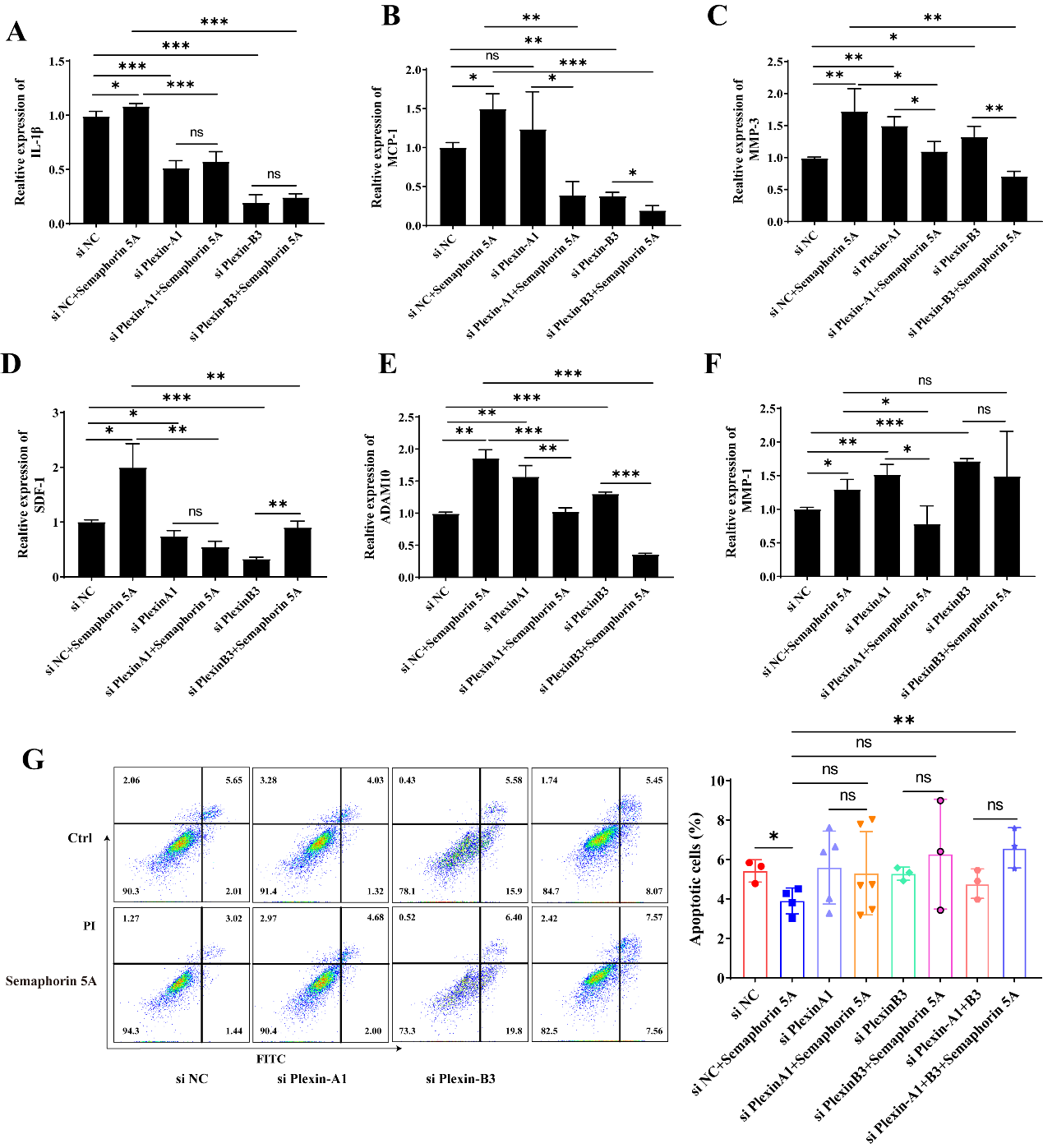


**Supplementary Figure 3.** Semaphorin 5A promotes cytokine secretion and inhibits apoptosis by SFs through binding to Plexin-A1 and Plexin-B3. **A**, mRNA levels of IL-1β in SFs detected by qPCR (*n* = 3) after transfection of siRNAs to knock down Plexin-A1 and Plexin-B3. **B**, mRNA levels of MCP-1 in SFs detected by qPCR (*n* = 3) after transfection of siRNAs to knock down Plexin-A1 and Plexin-B3. **C**, mRNA levels of MMP-3 in SFs detected by qPCR (*n* = 3) after transfection of siRNAs to knock down Plexin-A1 and Plexin-B3. **D**, mRNA levels of SDF-1 in SFs detected by qPCR (*n* = 3) after transfection of siRNAs to knock down Plexin-A1 and Plexin-B3. **E**, mRNA levels of ADAM10 in SFs detected by qPCR (*n* = 3) after transfection of siRNAs to knock down Plexin-A1 and Plexin-B3. **F**, mRNA levels of MMP-1 in SFs detected by qPCR (*n* = 3) after transfection of siRNAs to knock down Plexin-A1 and Plexin-B3. **G**, Apoptosis of SFs detected by flow cytometry (*n* = 3-6) after transfection of siRNAs to knock down Plexin-A1 and Plexin-B3. **P* < 0.05; ***P* < 0.01; ****P* < 0.001; ns, not significant. Values of *P* < 0.05 were considered significant. SFs, synovial fibroblasts.

**
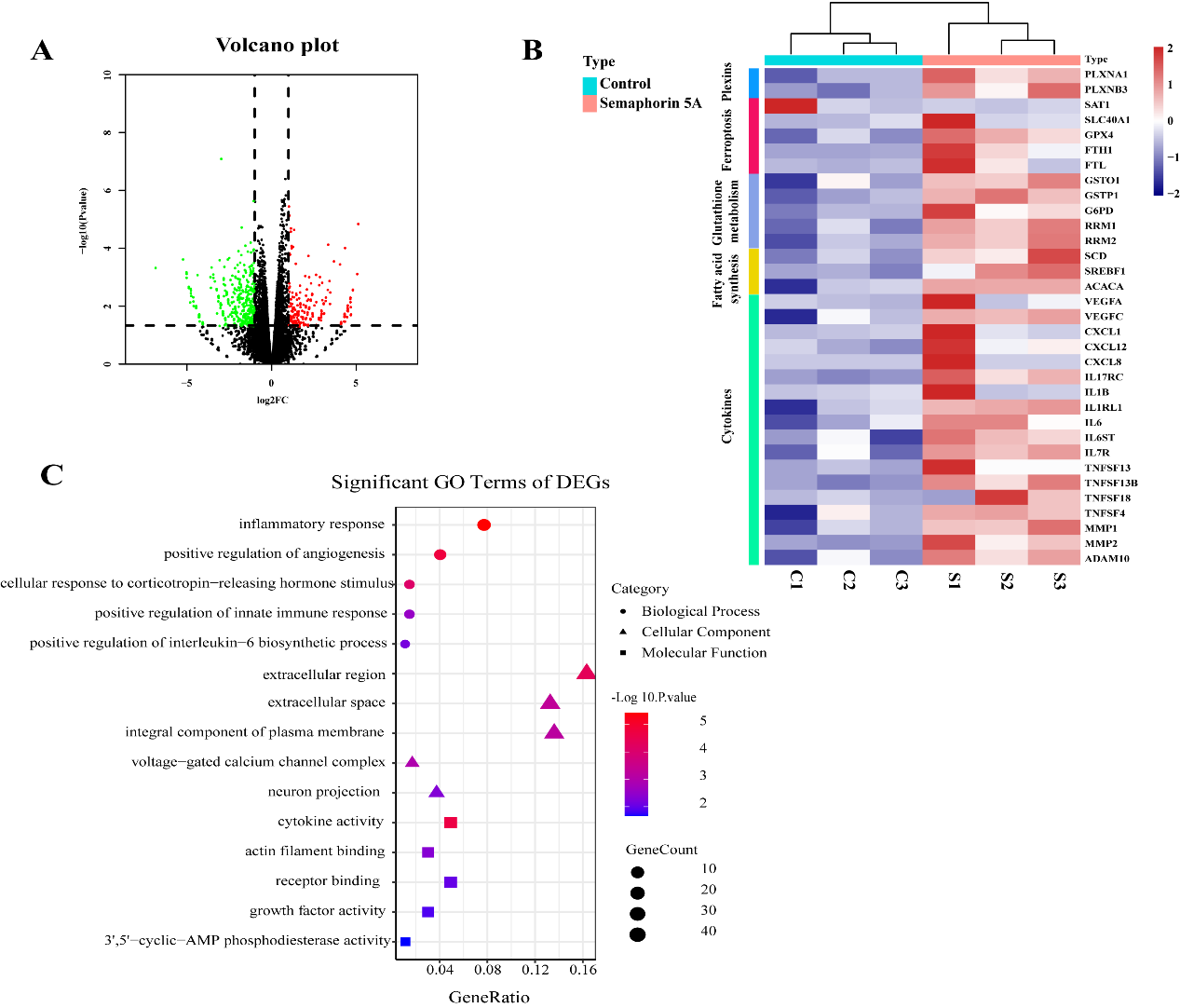
**

**Supplementary Figure 4.** Transcriptome sequencing results. **A**, Volcano plot of differentially expressed genes (DEGs) between the control group and the Semaphorin 5A treatment group. The red plots represent up-regulated genes, the black plots represent non-significant genes, and the green plots represent down-regulated genes. **B**, Heatmap of Representative genes between the control group and the Semaphorin 5A treatment group. The red rectangles represent high expression and the blue rectangles represent low expression. **C**, Significant GO terms of the DEGs. **P* < 0.05; ***P* < 0.01; ****P* < 0.001; ns, not significant. Values of *P* < 0.05 were considered significant. SFs, synovial fibroblasts.


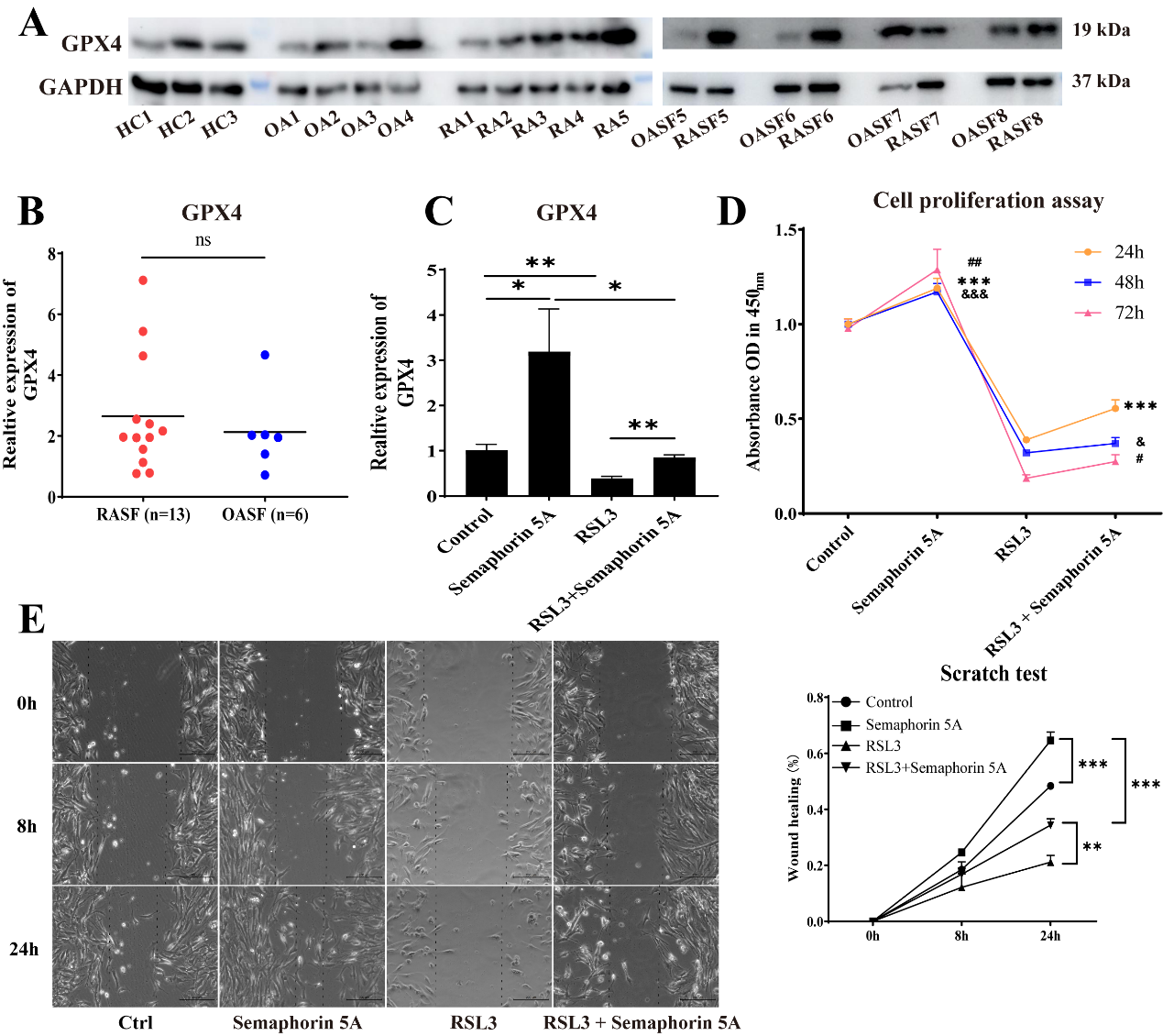
**Supplementary Figure 5.** The expression of GPX4 and inhibitory effect of RSL3 on activation of SFs. **A**, Protein levels of GPX4 in RA (*n* = 5), OA (*n* = 4) and healthy control (*n* = 3) synovial tissues (Left) and RA (*n* = 4), OA (*n* = 4) SFs (Right) detected by western blotting. **B**, mRNA levels of *GPX4* in RA (*n* = 13) and OA (*n* = 6) SFs detected by qPCR. **C**, mRNA levels of GPX4 in SFs detected by qPCR (*n* = 3) after co-treatment with RSL3 (150 nM) and Semaphorin 5A (1 μg/mL). **D**, Proliferation of SFs detected by a Cell Counting Kit-8 (*n* = 4) after co-treatment with RSL3 (150 nM) and Semaphorin 5A (1 μg/mL) at 24, 48, and 72 h. **E**, Migration of SFs detected by wound healing assays (*n* = 4) after co-treatment with RSL3 (150 nM) and Semaphorin 5A (1 μg/mL). **P* < 0.05; ***P* < 0.01; ****P* < 0.001; ns, not significant; ^&^*P* < 0.05; ^&&&^*P* < 0.01; ^#^*P* < 0.05; ^##^*P* < 0.01. Values of *P* < 0.05 were considered significant. SFs, synovial fibroblasts.

**
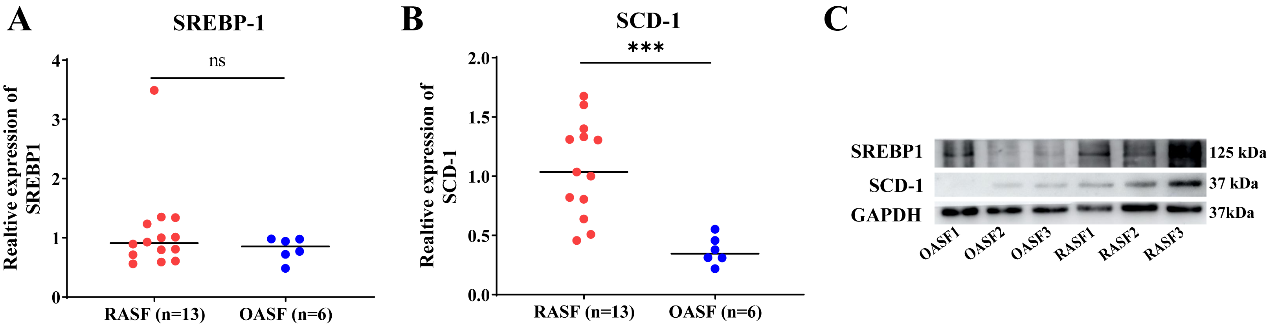
**

**Supplementary Figure 6.** The expression of SREBP1 and SCD-1 in RA and OA SFs. A-B, mRNA levels of *SREBP1* and *SCD-1* in RA (*n* = 13) and OA (*n* = 6) SFs detected by qPCR. C. Protein levels of SREBP1 and SCD-1 in RA (*n* = 3) and OA (*n* = 3) SFs detected by western blotting.

**Supplementary Tables**

**Table 1 Clinical and laboratory features in patients with rheumatoid arthritis (RA) and osteoarthritis (OA) (synovia)**

| Characteristic | RA (n=30) | OA (n=26) |
| --- | --- | --- |
| Age, years | 58 (29-80) | 61 (48-86) |
| Gender, female/male | 24/6 | 17/9 |
| Duration, years | 6 (0.25-30) | 4.75 (0.25-10) |
| Smoking, n (%) | 3 (10) | 1 (3.84) |
| Number of tender joints | 2 (0-16) | / |
| Number of swollen joints | 3 (0-16) | / |
| **Laboratory features** |  |  |
| RF, positive, n (%) | 22 (73.3) | 0 (0) |
| Anti-CCP, positive, n (%) ^a^ | 20 (83.3) | 0 (0) |
| CRP (mg/l) | 38.24 (0.26-306) | 6.3 (0.21-28.7) |
| ESR (mm/h) | 51 (7-105) | 15.5 (2-36) |
| **Disease activity score** | | |
| DAS28-CRP | 4.06±1.13 | / |
| DAS28-ESR | 3.56±1.2 | / |
| **Treatment** | | |
| NSAIDs, n (%) | 21 (70) | 22 (84.6) |
| Methotrexate, n (%) | 14 (46.7) | / |
| Leflunomide, n (%) | 20 (66.7) | / |
| Tripterygium glycosides | 10 (33.3) | / |
| Glucocorticoid | 19 (63.3) | / |
| Hydroxychloroquine, n (%) | 3 (10) | / |
| Iguratimod, n (%) | 5 (16.7) | / |
| Salazosulfapyridine, n (%) | 2 (6.67) |  |
| Biologics, n (%) | 5 (16.7) |  |

The values shown represent the number (%) or median (range). RF, rheumatoid factor; Anti-CCP, Anti-cyclic citrullinated peptide antibody; CRP, C-reactive protein; ESR, erythrocyte sedimentation rate; DAS28, Disease activity score of 28 joints; NSAIDs, Non-Steroidal Anti-inflammatory Drugs. ^a^ 6 RA and 13 OA patients did not detect anti-CCP antibody.

**Table 2 Correlations between the level of Semaphorin 5A and clinical features in RA patients**

| Characteristic | Spearman r | *P* value |
| --- | --- | --- |
| Age | -0.07 | 0.78 |
| Duration | -0.31 | 0.18 |
| RF | 0.68 | 0.02 |
| Anti-CCP | -0.06 | 0.84 |
| CRP | 0.68 | 0.001 |
| ESR | 0.50 | 0.03 |
| TJC | 0.07 | 0.79 |
| SJC | 0.05 | 0.84 |
| DAS28 (CRP) | 0.31 | 0.21 |
| DAS28 (ESR) | 0.51 | 0.03 |

RF, rheumatoid factor; Anti-CCP, Anti-cyclic citrullinated peptide antibody; CRP, C-reactive protein; ESR, erythrocyte sedimentation rate; DAS28, Disease activity score of 28 joints.

**Table 3 Clinical and laboratory features in patients with rheumatoid arthritis (RA) and osteoarthritis (OA) and healthy control (HC) subjects (synovial tissue samples)**

| Characteristic | RA (n=13) | OA(n=6) | HC (n=3) |
| --- | --- | --- | --- |
| Age, years | 54 (32-71) | 59 (47-84) | 51 (47-89) |
| Gender, Female/male | 11/2 | 5/1 | 2/1 |
| Duration, years | 9 (0.5-20) | 5 (0.5-10) | / |
| Smoking | 1 (7.69) | 0 (0) |  |
| **Laboratory features** | | | |
| RF, positive, n (%) | 10 (76.9) | 0 (0) | / |
| Anti-CCP, positive, n (%) ^a^ | 6 (75) | 0 (0) | / |
| CRP (mg/l) | 53.75 (5.6-180.2) | 24.45 (6.3-54.4) | / |
| ESR (mm/h) | 42 (14-87) | 17 (2-36) | / |
| **Treatment** | | | |
| NSAIDs | 10 (76.9) | 6 (100) | / |
| Methotrexate | 6 (46.2) | 0 (0) | / |
| Leflunomide | 4 (30.8) | 0 (0) | / |
| Tripterygium glycosides | 1 (7.69) | 0 (0) | / |
| Glucocorticoid | 3 (23.1) | 0 (0) | / |
| Hydroxychloroquine | 1 (7.69) | 0 (0) | / |
| Iguratimod | 1 (7.69) | 0 (0) | / |

Data was presented as number (%) or Median (range). RA, rheumatoid arthritis; OA, osteoarthritis; RF, rheumatoid factor; Anti-CCP, Anti-cyclic citrullinated peptide antibody; CRP, C-reactive protein; ESR, erythrocyte sedimentation rate; NSAIDs, Non-Steroidal Anti-inflammatory Drugs. ^a^ All synovial tissue was derived from arthroscopic surgery and 5 RA and 2 OA patients did not test anti-CCP.

**Table 4 Primer sequence of genes**

| \| Transcripts \|  \|  \| Sequence \| \| --- \| --- \| --- \| --- \| |
| --- | --- | --- | --- | --- |
| \| GAPDH \| Forward \| AAGGTGAAGGTCGGAGTCAA \| \| --- \| --- \| --- \| \|  \| Reverse \| AATGAAGGGGTCATTGATGG \| \| Semaphorin5A \| Forward \| GGTACTGTTCTAGCGACGGC \| \|  \| Reverse \| ATACTTGGGTTCGGGGTTGT \| \| Plexin-A1 \| Forward \| ACCCACCTAGTGGTGCATGA \| \|  \| Reverse \| CGGTTAGCGGCATAGTCCA \| \| Plexin-B3 \| Forward \| CGCTTCTCCGCACCTAATACC \| \|  \| Reverse \| CAGGGCTGTCGATTACAGGG \| \| IL-1β \| Forward \| GGACAAGCTGAGGAAGATGC \| \|  \| Reverse \| TCGTTATCCCATGTGTCGAA \| \| IL-6 \| Forward \| GCCCAGCTATGAACTCCTTCT \| \|  \| Reverse \| GAAGGCAGCAG GCAACAC \| \| IL-8 \| Forward \| TTGGCAGCCTTCCTGATTTC \| \|  \| Reverse \| AACTTCTCCACAACCCTCTG \| \| ADAM10 \| Forward \| CGGAACACGAGAAGCTGTGATT \| \|  \| Reverse \| TTACGGATTCCGGAGAAGTCTGT \| \| VEGF \| Forward \| CTACCTCCATGCCAAG \| \|  \| Reverse \| GCAGTAGCTGCGCTGATAGA \| \| MCP-1 \| Forward \| GATCTCAGTGCAGAGGCTCG \| \|  \| Reverse \| TGCTTGTCCAGGTGGTCCAT \| \| CD147 \| Forward \| TCGCGCTGCTGGGCACC \| \|  \| Reverse \| TGCCGCTGTCATTCAAGGA \| \| SDF-1 \| Forward \| TTTCCTCAATTTCTCCTCGG \| \|  \| Reverse \| ATGCGTCACGCTATGTGCT \| \| MMP-2 \| Forward \| ATGACAGCTGCACCACTGAG \| \|  \| Reverse \| ATTTGTTGCCCAGGAAAGTG \| \| MMP-3 \| Forward \| CACAGACCTGACTCGGTTCC \| \|  \| Reverse \| GATTTGCGCCAAAAGTGCCT \| \| SLC40A1 \| Forward \| ACATCCGATCTCCCCAAGTAG \| \|  \| Reverse \| ACATCCGATCTCCCCAAGTAG \| \| GPX4 \| Forward \| GAGGCAAGACCGAAGTAAACTAC \| \|  \| Reverse \| CCGAACTGGTTACACGGGAA \| \| SREBF1 \| Forward \| GCTGCTGACCGACATCGAA \| \|  \| Reverse \| GGGTGGGTCAAATAGGCCAG \| \| SCD-1 \| Forward \| CCGGACACGGTCACCCGTTG \| \|  \| Reverse \| CGCCTTGCACGCTAGCTGGT \| |

**Table 5 Sequence of siRNA**

| Transcripts |  | Sequence |
| --- | --- | --- |
| Plexin-A1 | siRNA-1 | GCAGUACUGACAACGUCAATT |
|  | siRNA-2 | GGAACGGCAACUUUGUCAUTT |
| Plexin-B3 | siRNA-1 | ACAUGAGCCAAGCUGUCAUAG |
|  | siRNA-2 | UAAAGAGCAUGGGUGUUGUCC |
| SREBP1 | siRNA-1 | CCUAUUUGACCCACCCUAUTT |
|  | siRNA-2 | GGAGGCUUCUCUACAGGAATT |
| SCD-1 | siRNA-1 | GACGAUAUCUCUAGCUCCUTT |
|  | siRNA-2 | GGUUGAAUAUGUCUGGAGATT |
