# Supplementary material for: Semaphorin 5A suppresses ferroptosis through activation of PI3K-AKT-mTOR signaling in rheumatoid arthritis
Source: Cell Death Dis. 2022 Jul 14;13(7):608. doi: 10.1038/s41419-022-05065-4 (PMC9283415; doi:10.1038/s41419-022-05065-4)

GAPDH


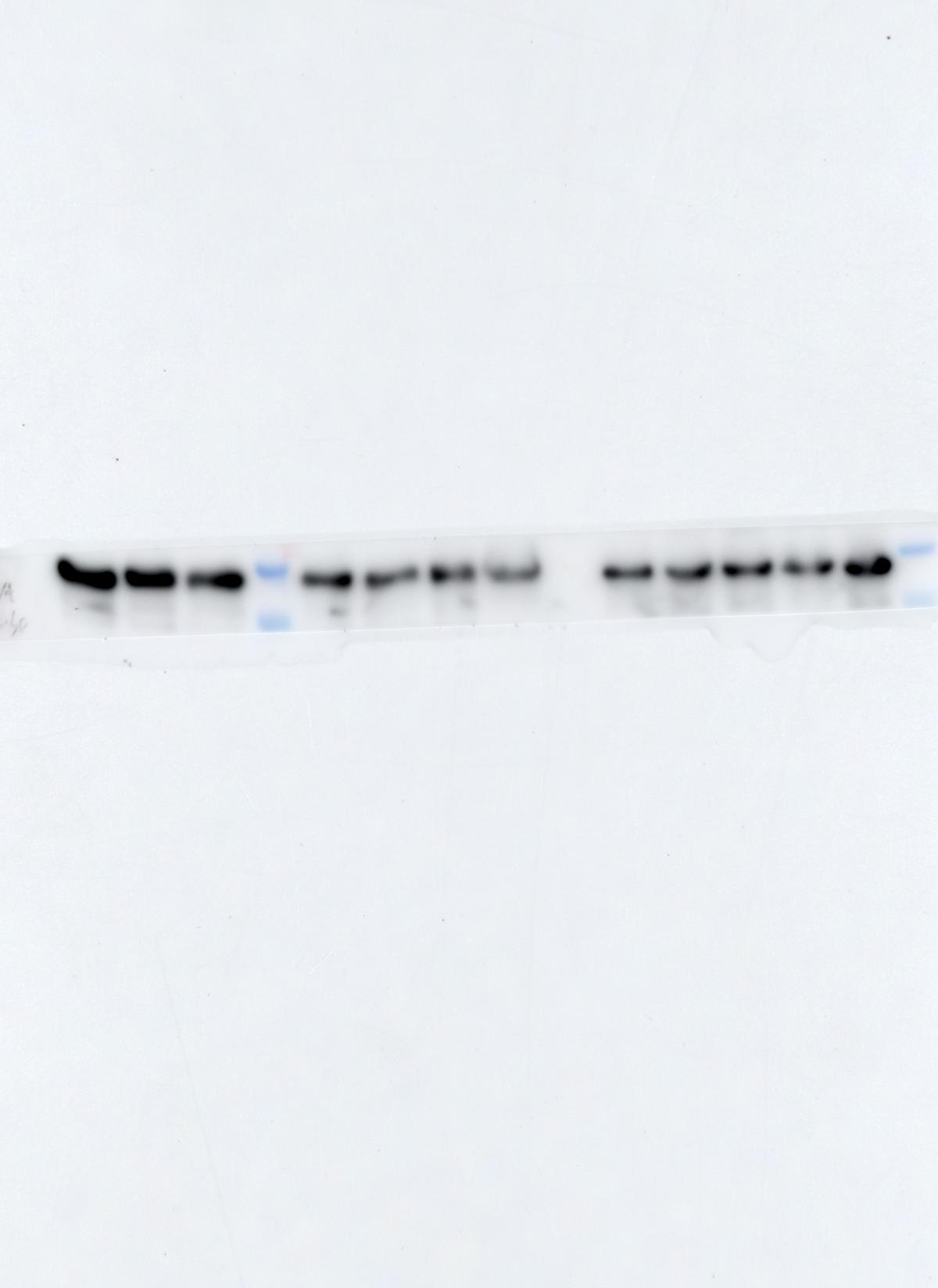


**Figure 1D**

Semaphorin 5A


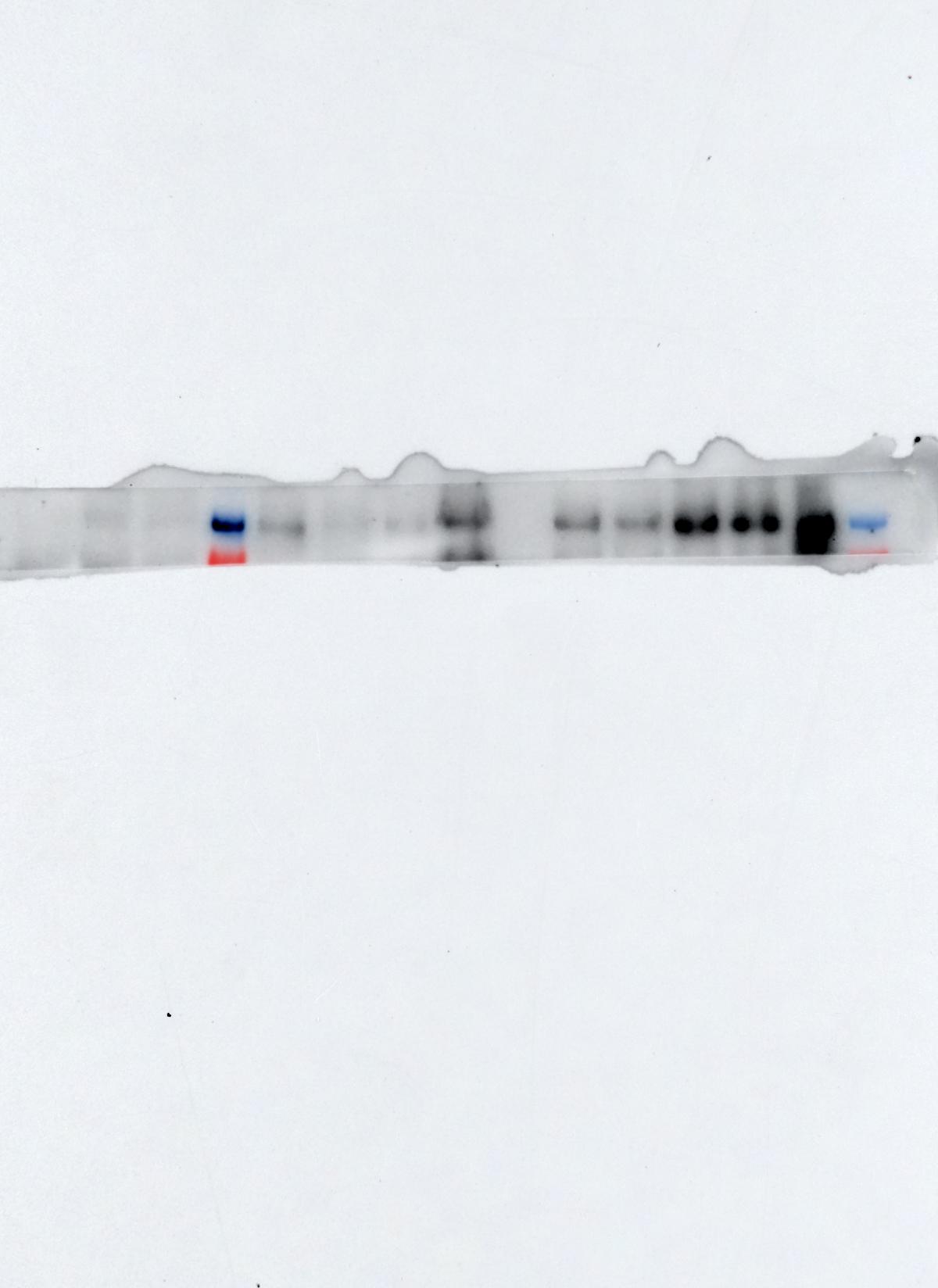


GAPDH


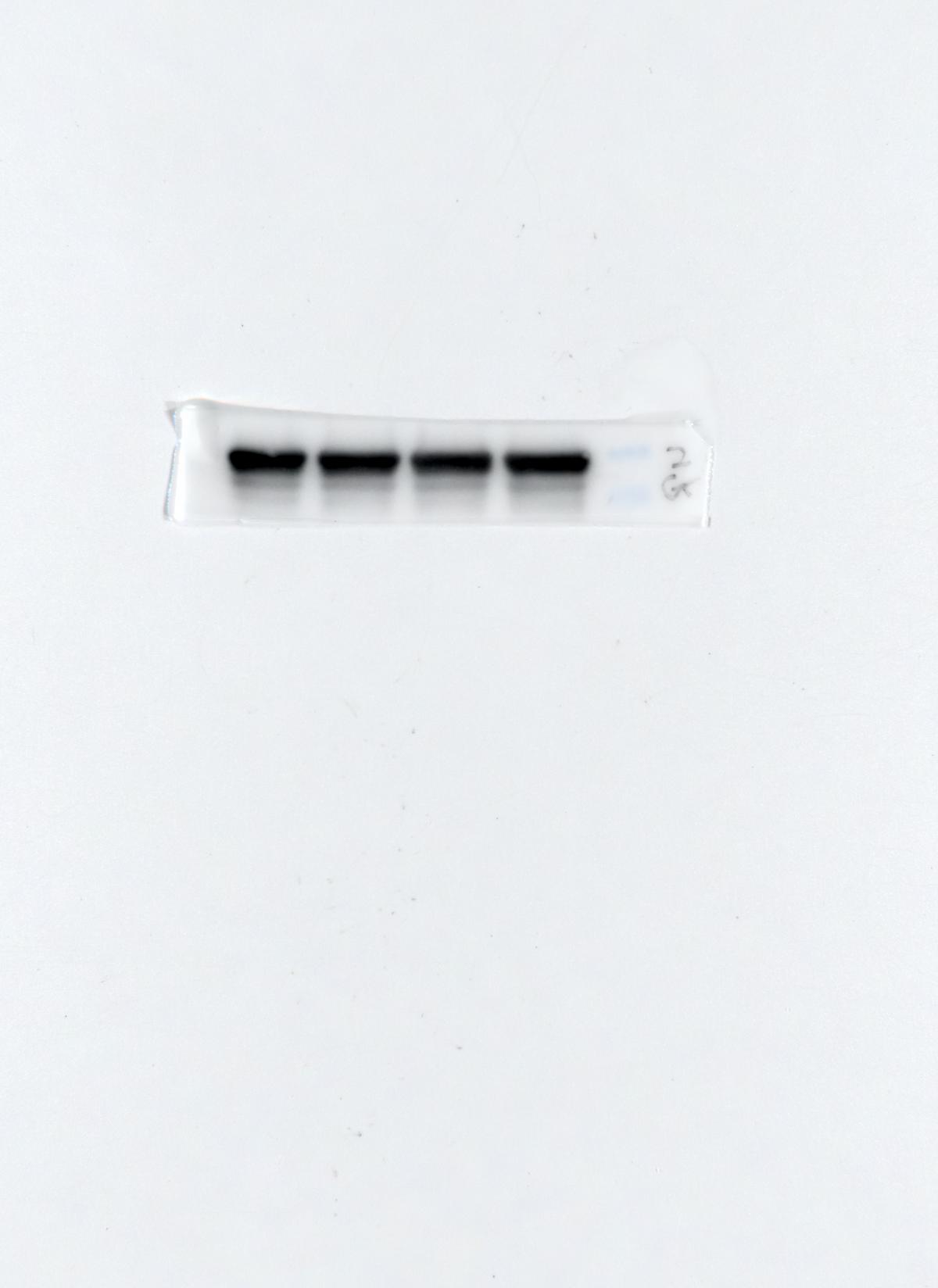


**Figure 2B**

Semaphorin 5A


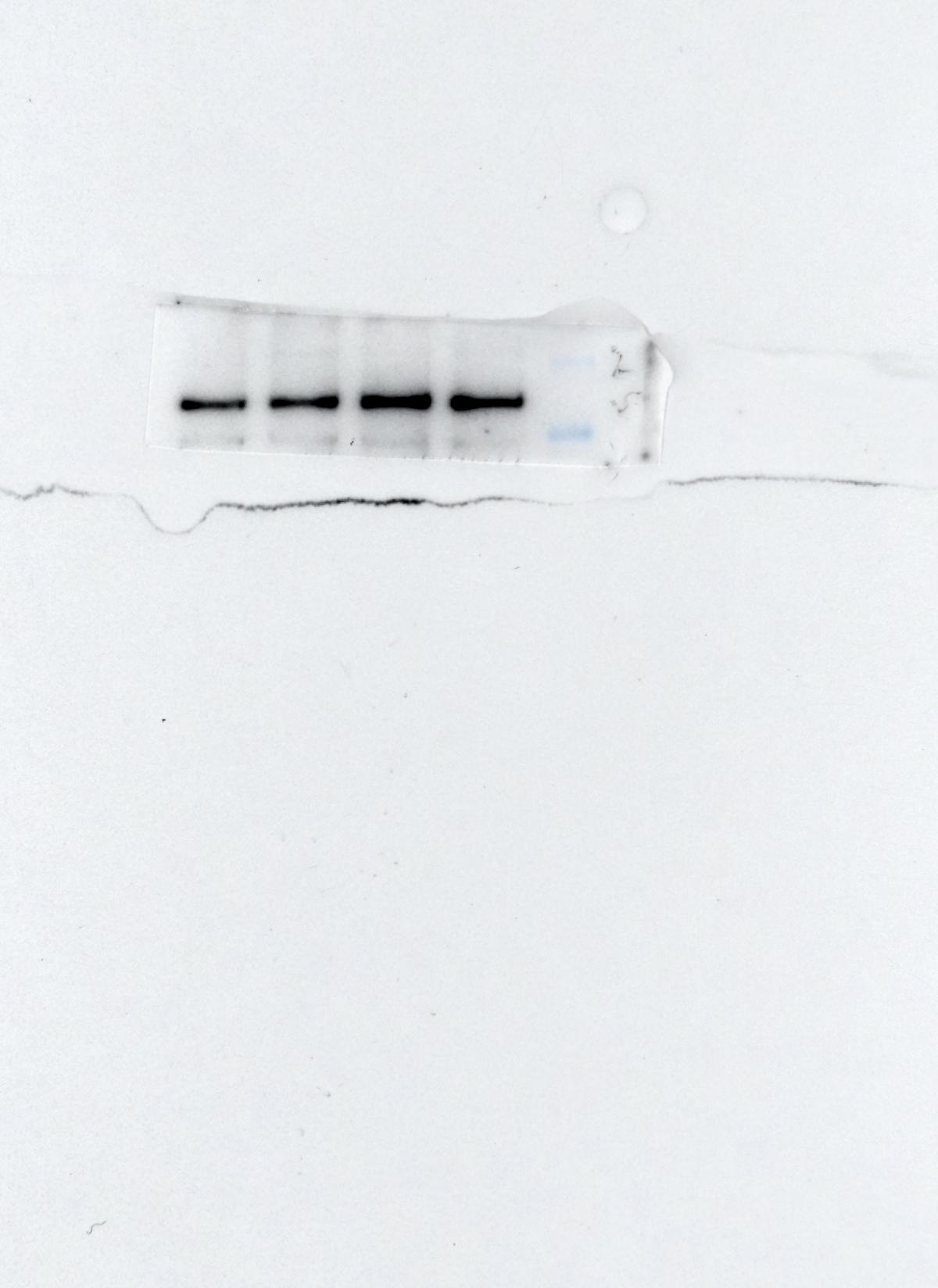


GAPDH

**Figure 3C**

**1: Control-1**

**2: Semaphorin 5A-1**

**3: Control-2**

**4: Semaphorin 5A-2**

**5: Control-3**

**6: Semaphorin 5A-3**


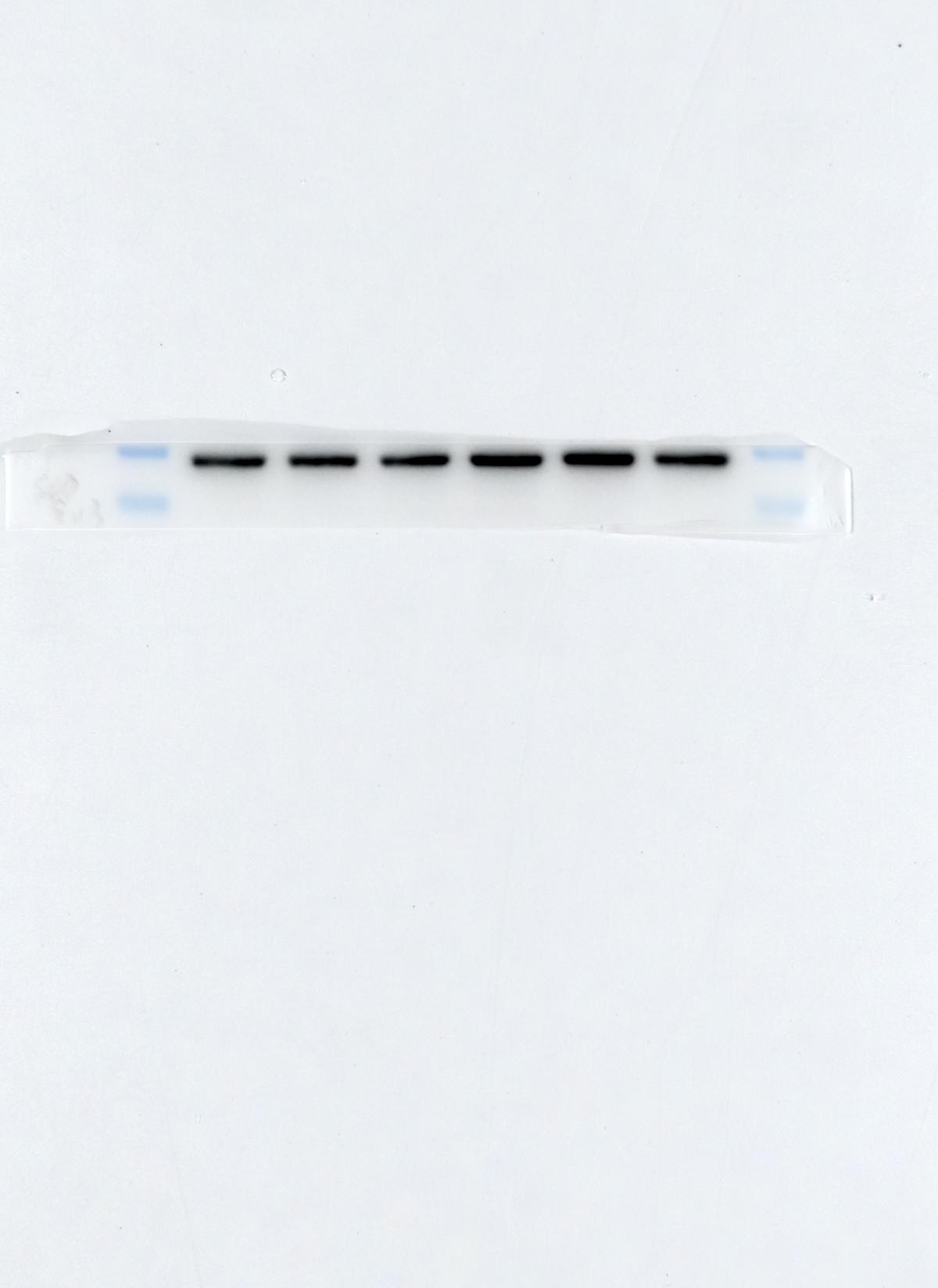


1 2 3 4 5 6

Plexin-A1


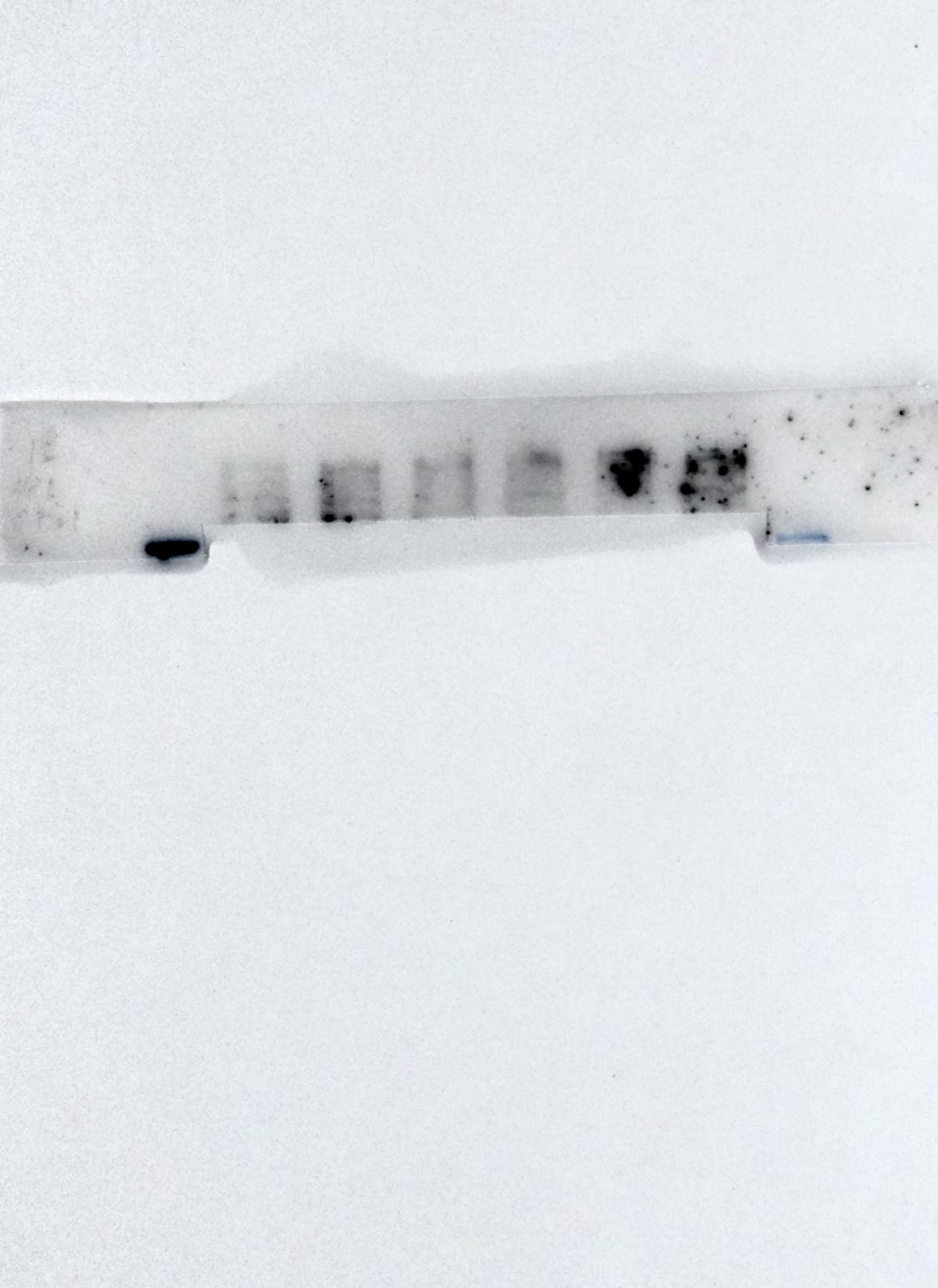


Plexin-B3


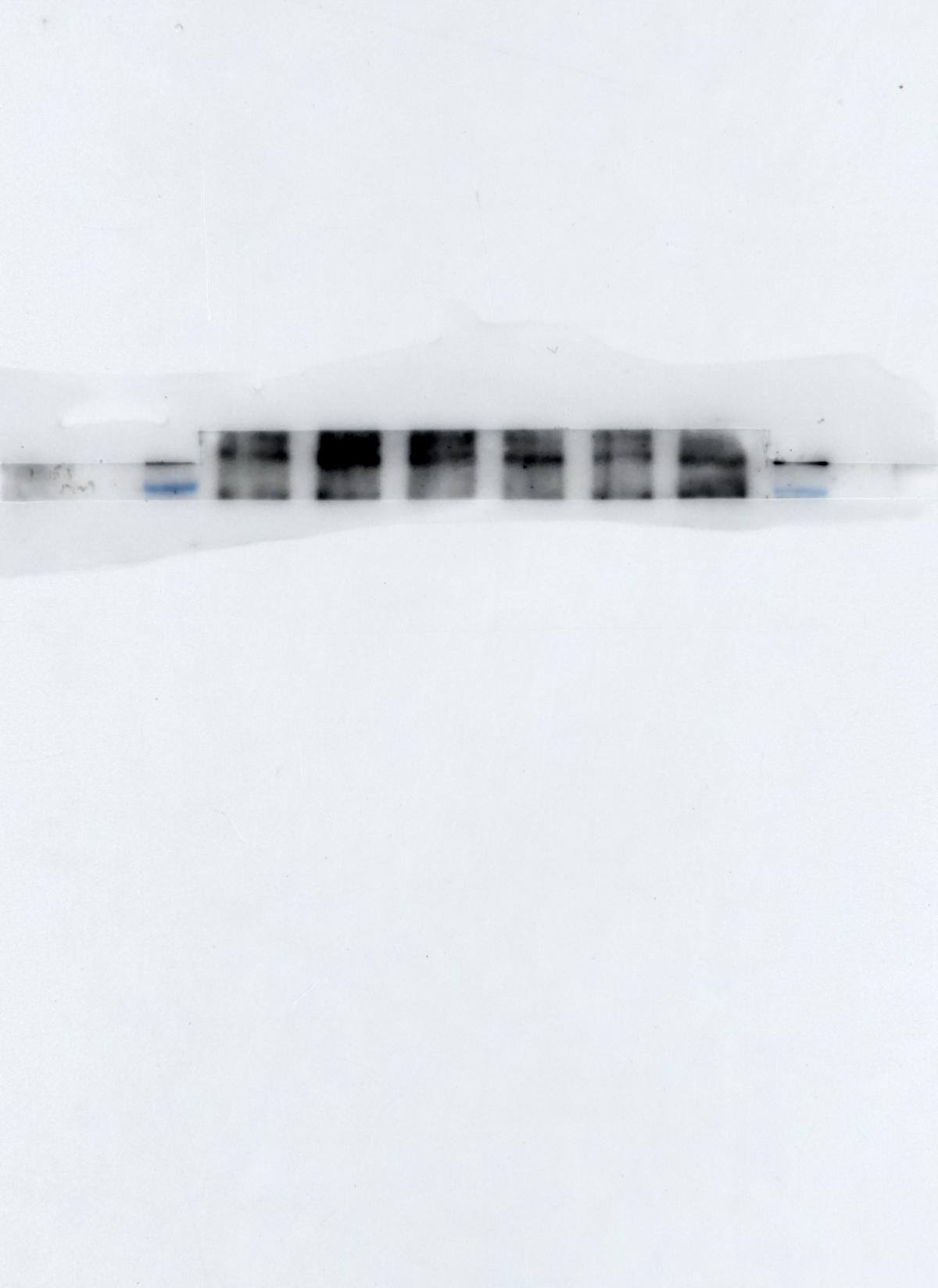


GAPDH


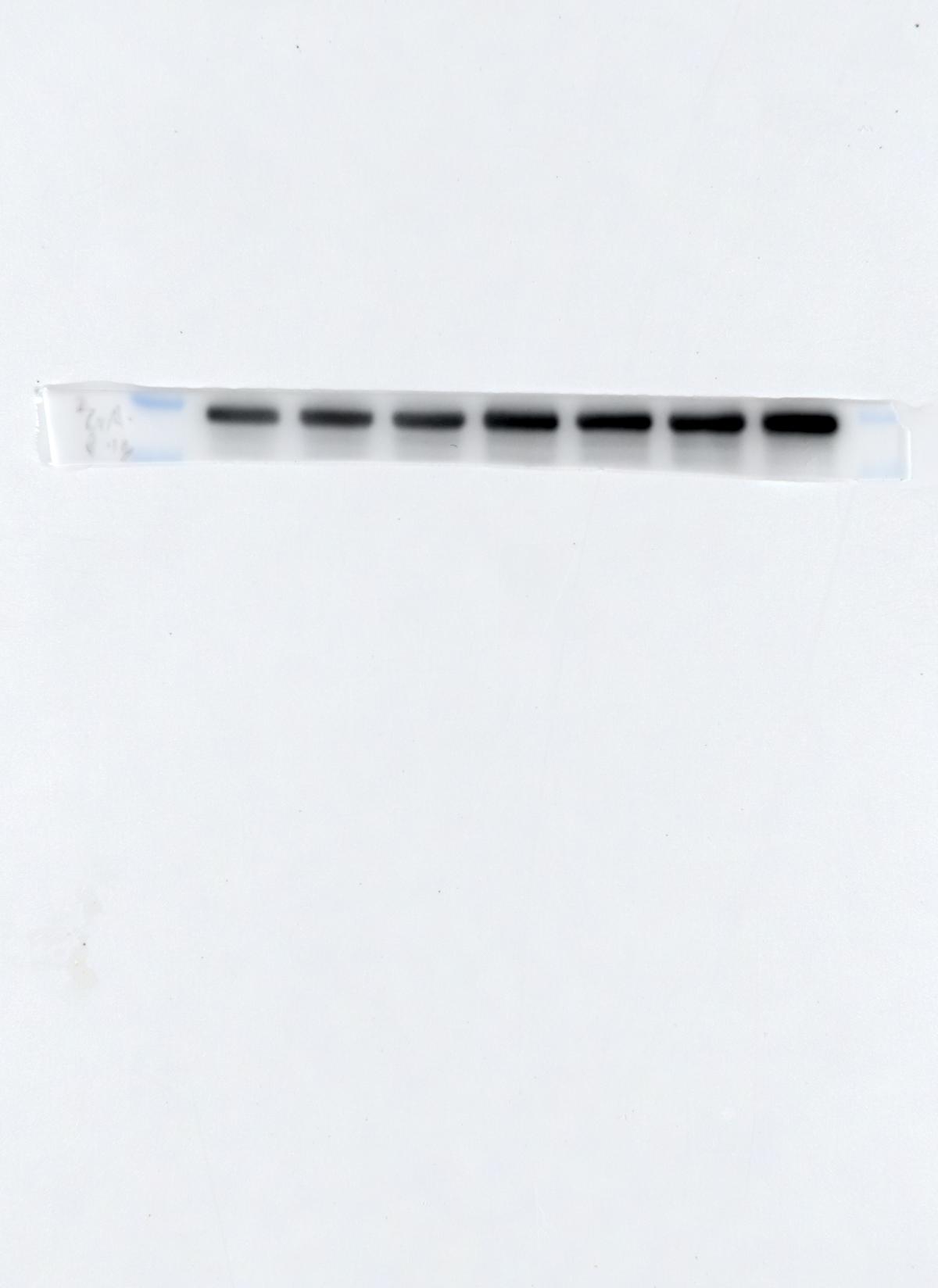


1 2 3 4 5 6 7

**Figure 3D**

**1: si NC-1**

**2: si Plexin-B3-1**

**3: si Plexin-B3-2**

**4: si NC-2**

**5: si Plexin-A1-mix**

**6: si Plexin-A1-1**

**7: si Plexin-A1-2**

Plexin-A1


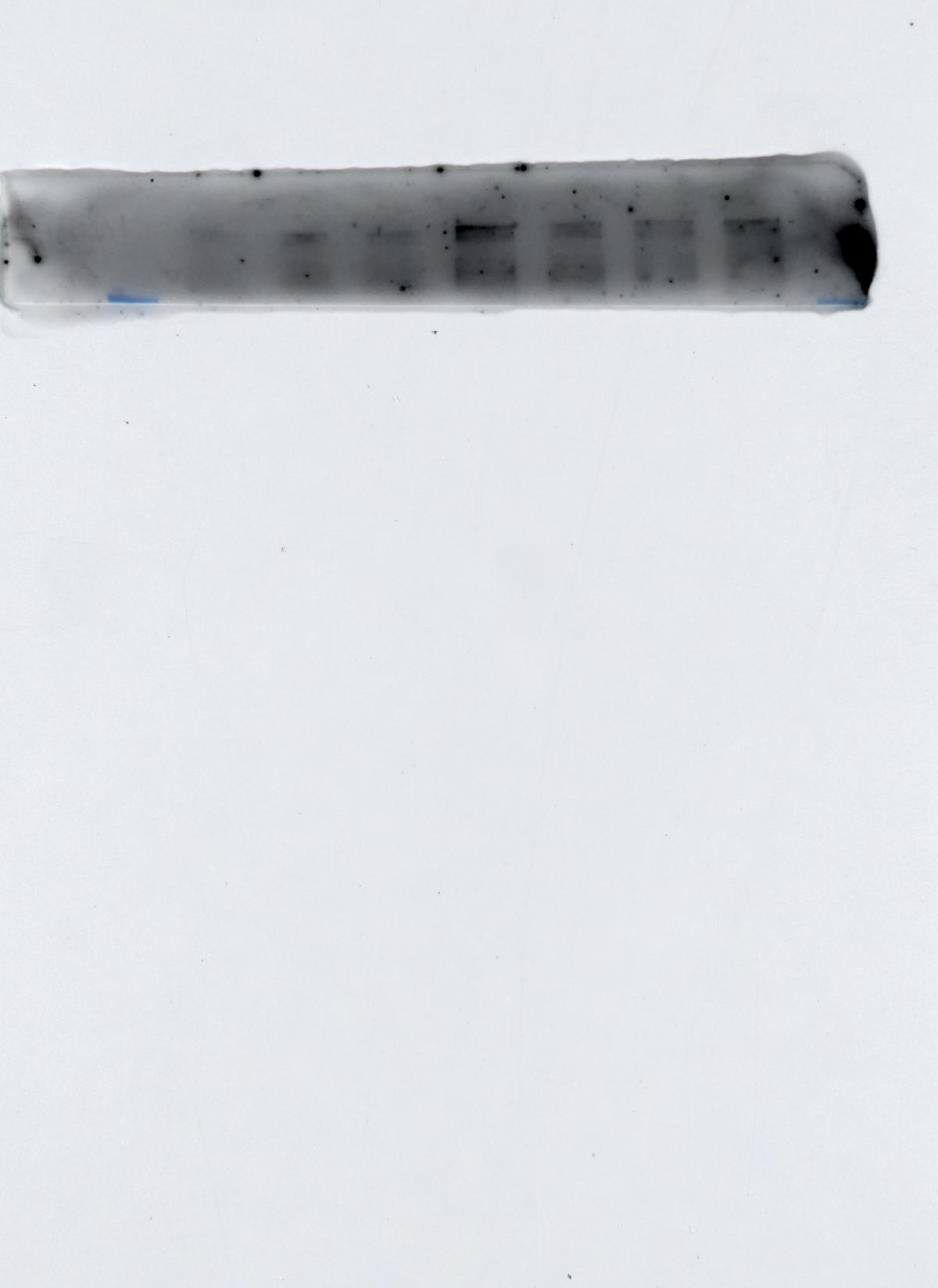


180kDa

GAPDH


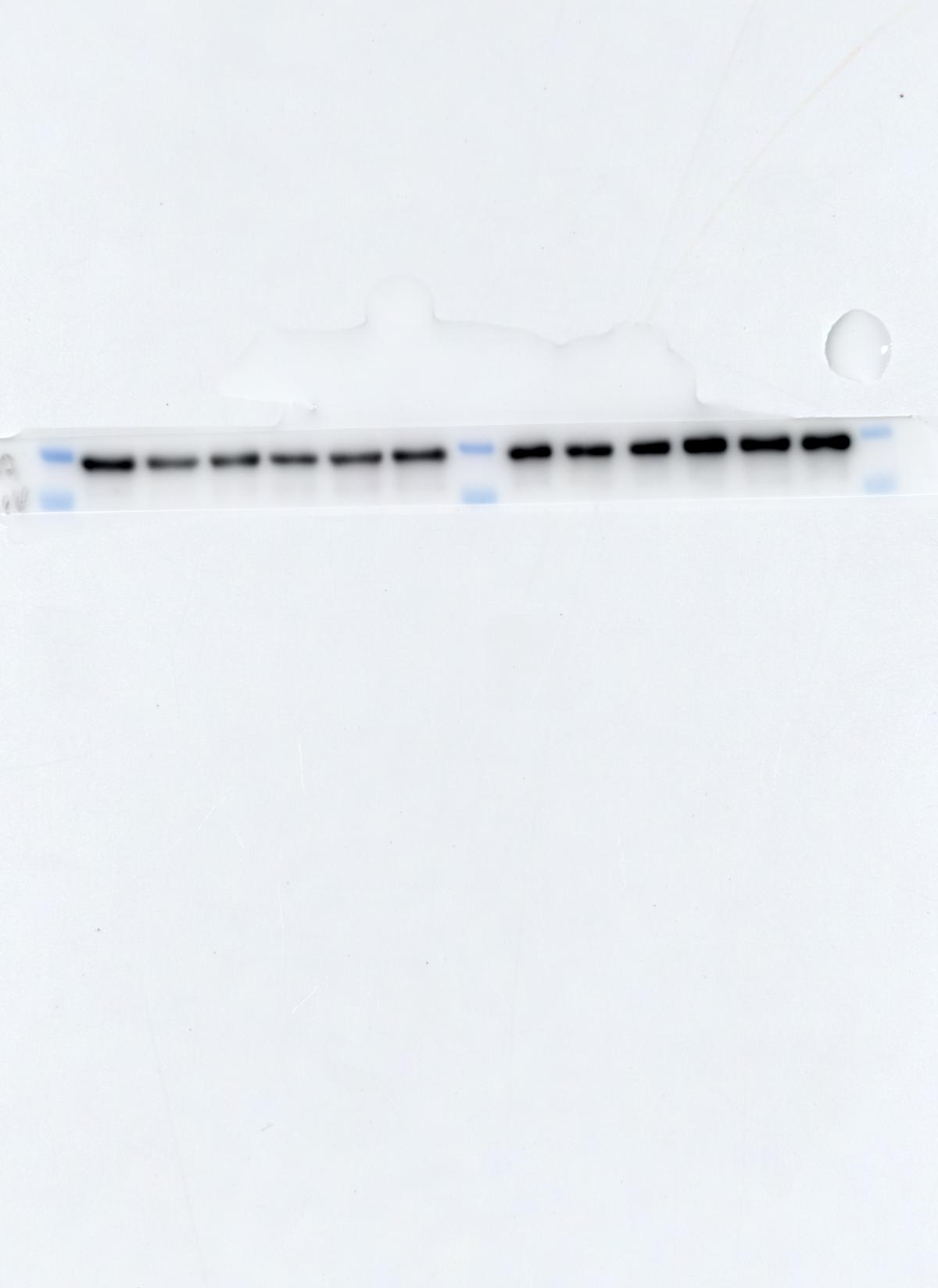


**Figure 3E**

**1-3:OA(synovial fibroblasts)**

**4-6:RA(synovial fibroblasts)**

**7: si NC-1**

**8: si Plexin-A1-1**

**9: si Plexin-A1-2**

**10: si NC-2**

**11: si Plexin-B3-1**

**12: si Plexin-B3-2**

40kDa

35kDa

1 2 3 4 5 6 7 8 9 10 11 12

Plexin-B3


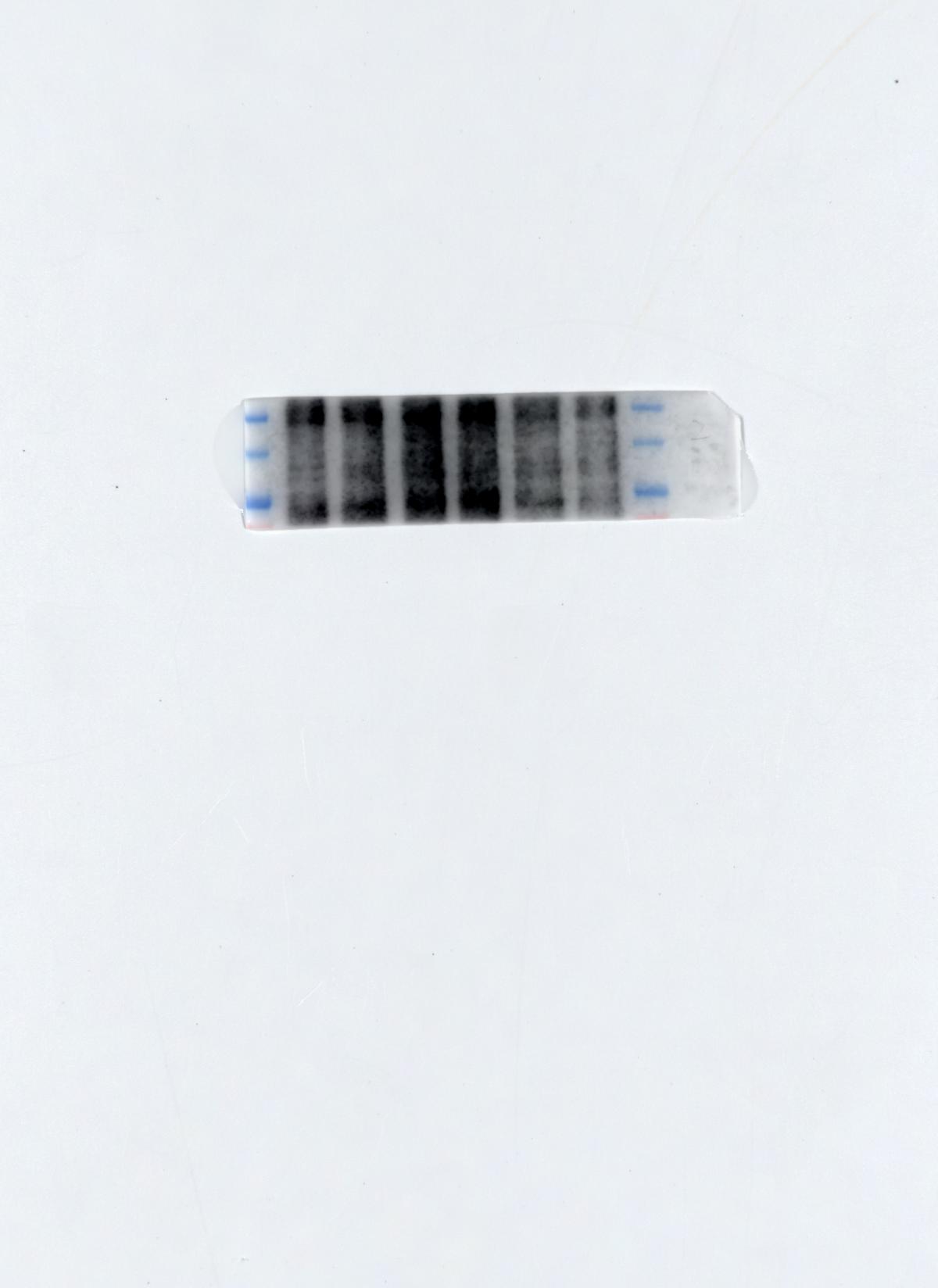


7 8 9 10 11 12

180kDa

130kDa

100kDa

GAPDH


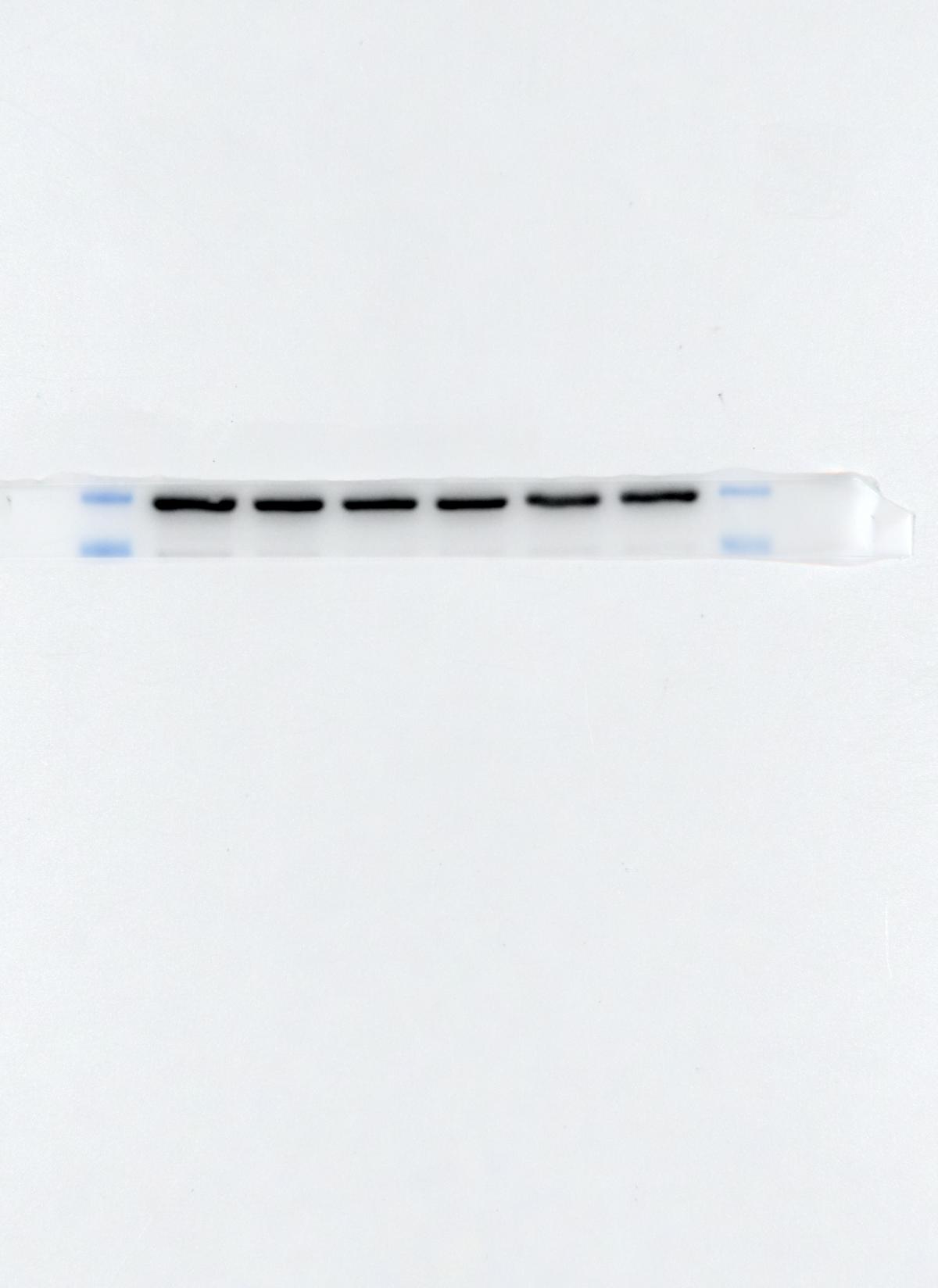


**Figure4C**

**1: Control**

**2: 2h**

**3: 8h**

**4: 24h**

**5: 48h**

**6: 72h**

1 2 3 4 5 6

PI3K


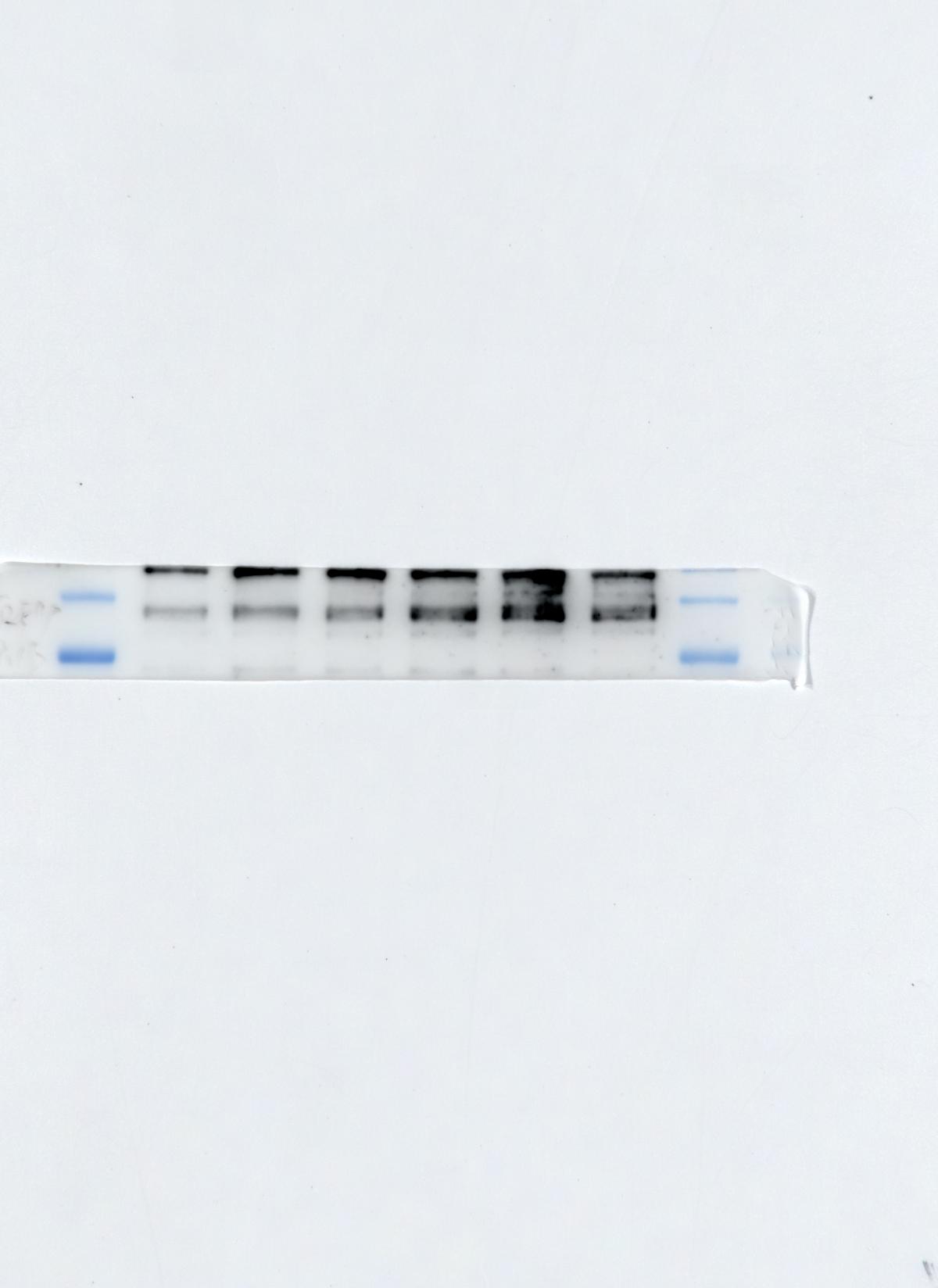


130kDa

100kDa

mTOR


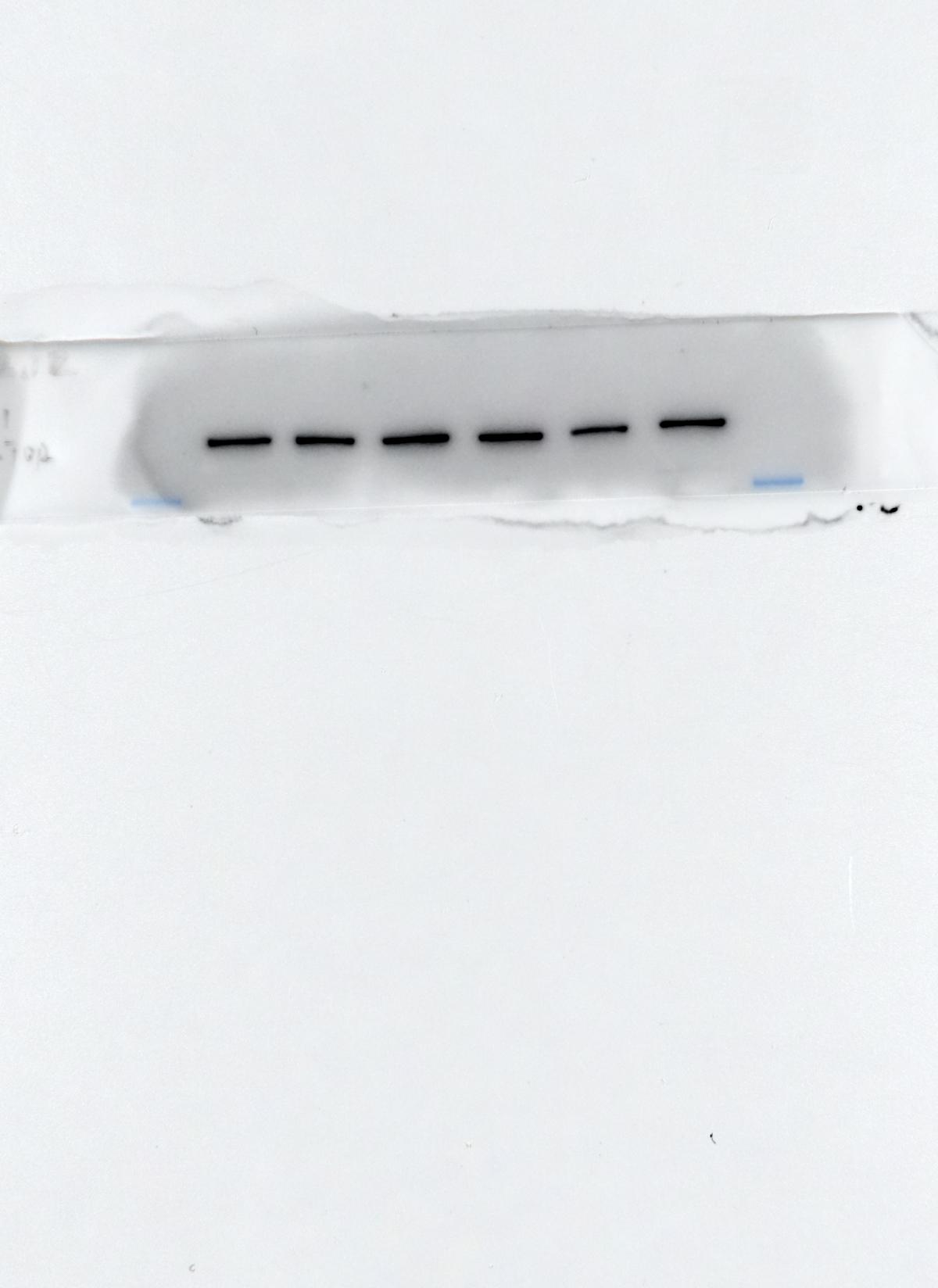


p-mTOR


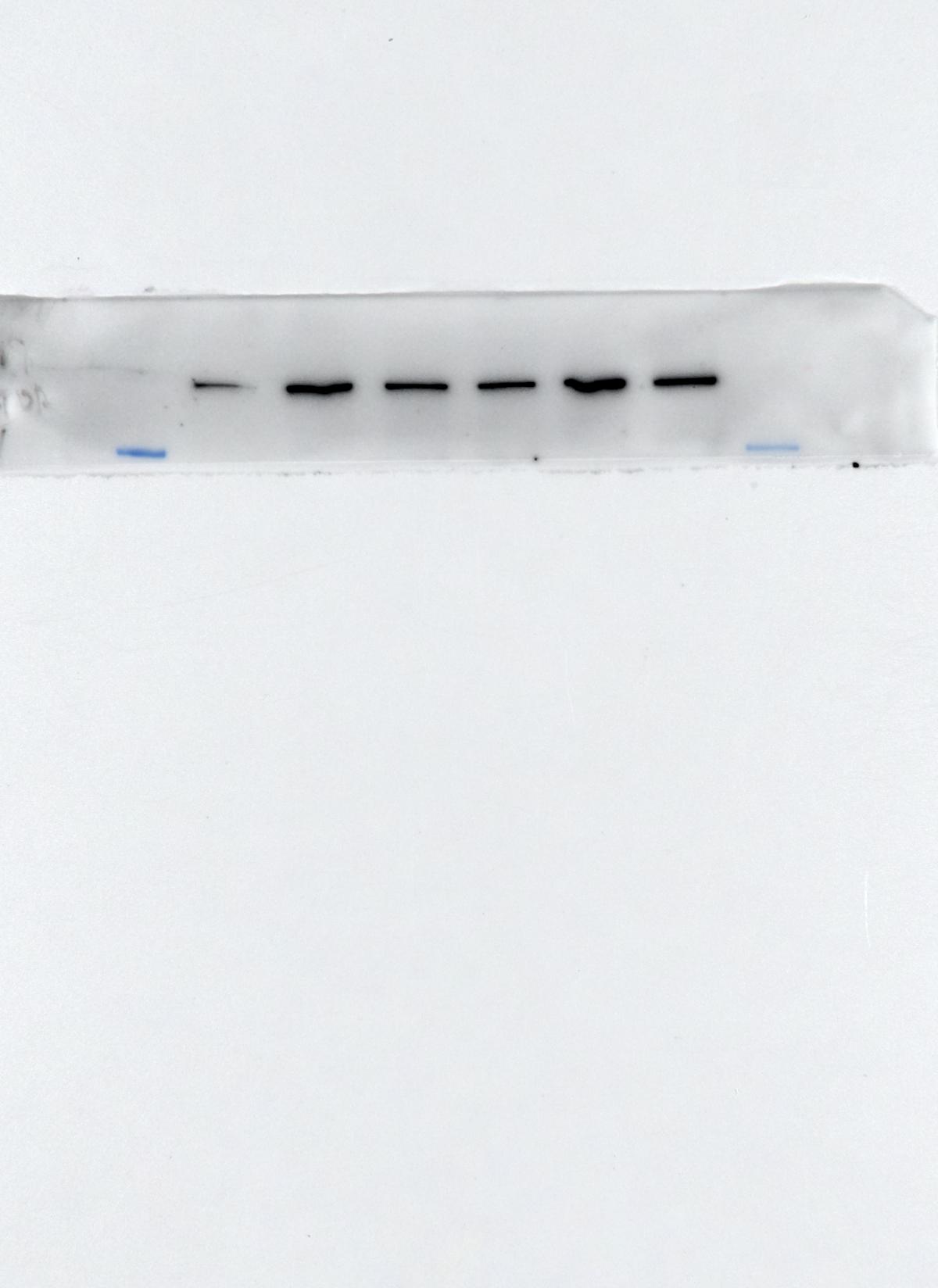


AKT


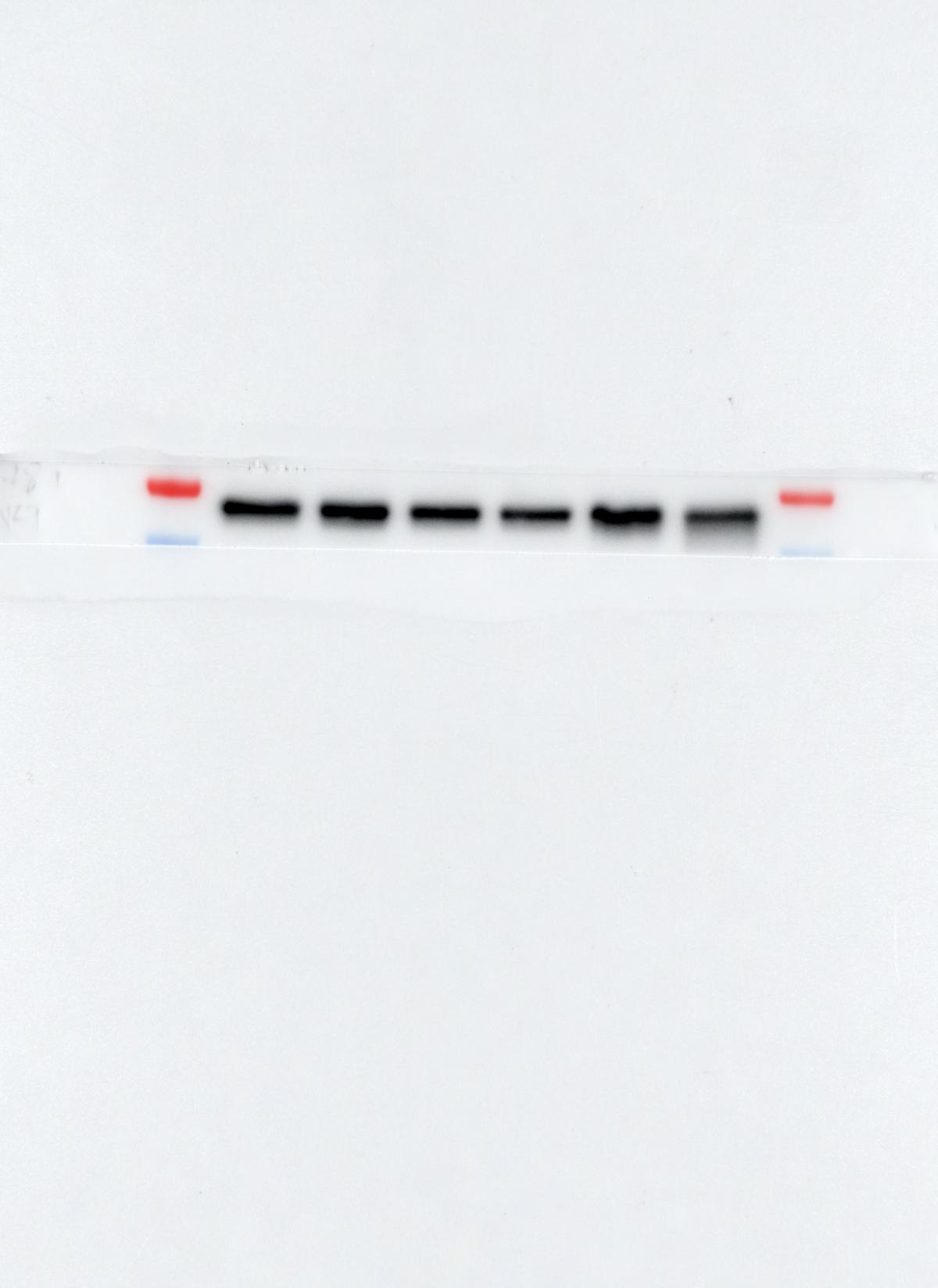


p-AKT


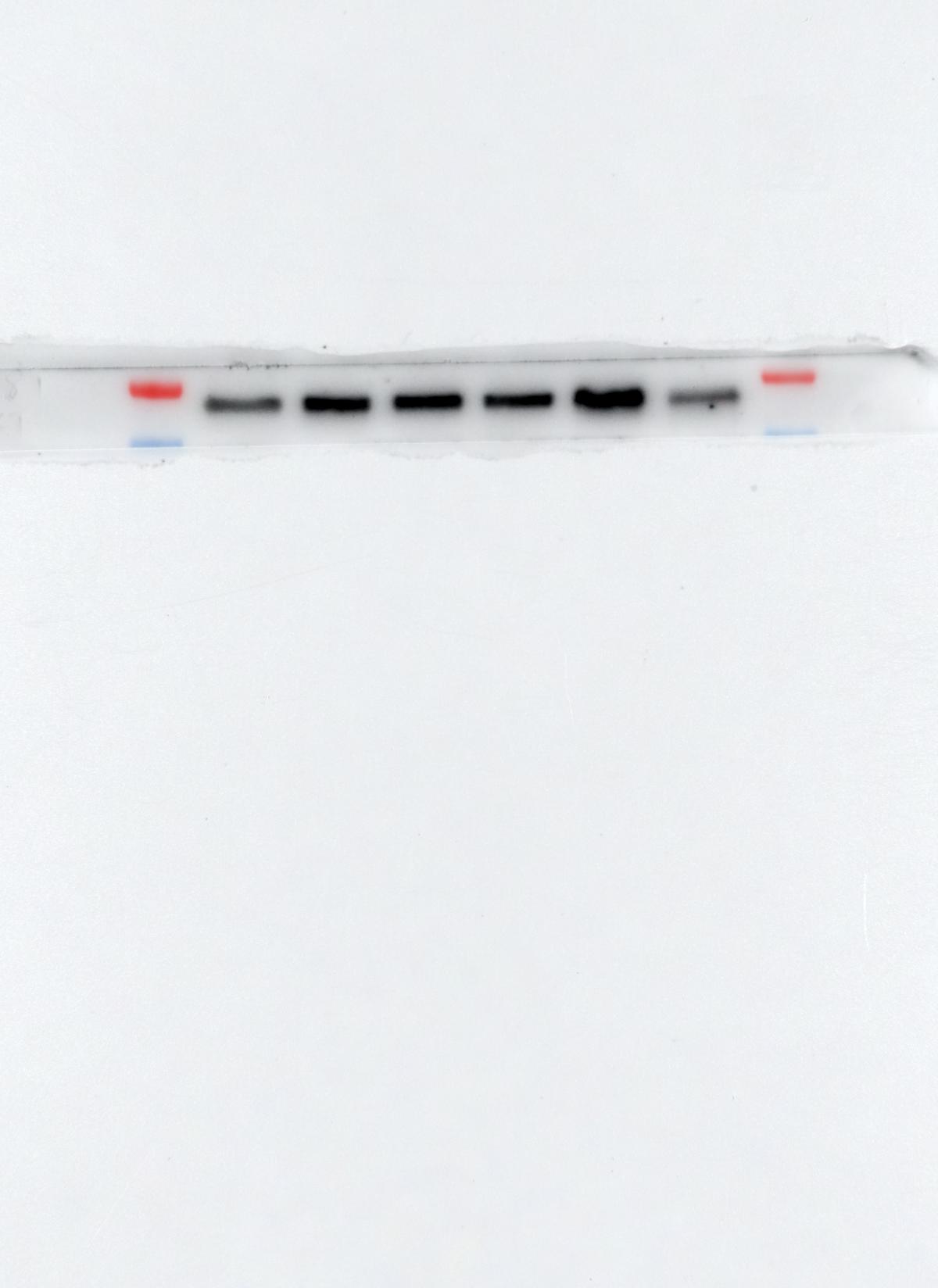


4EBP1


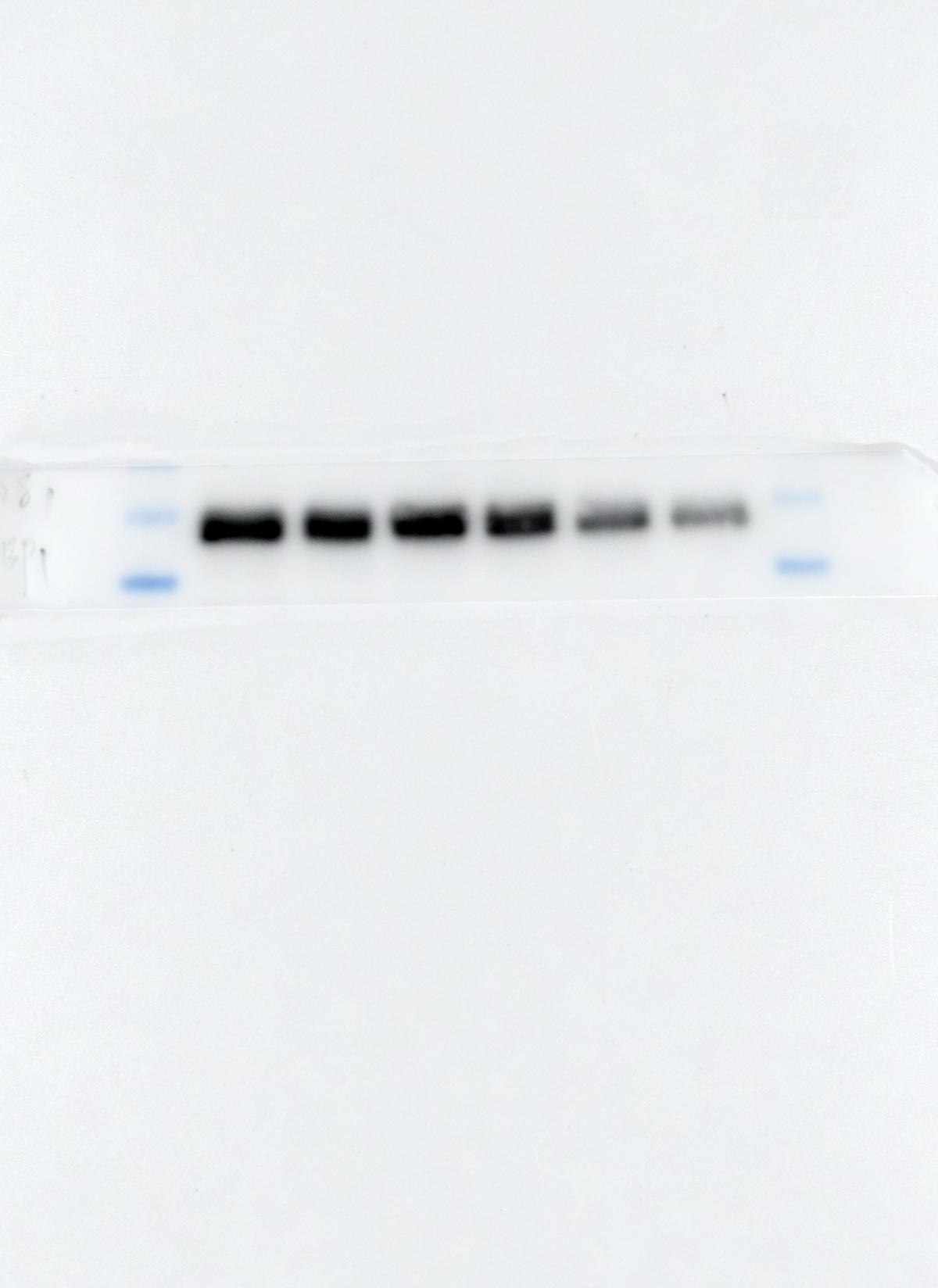


p-4EBP1


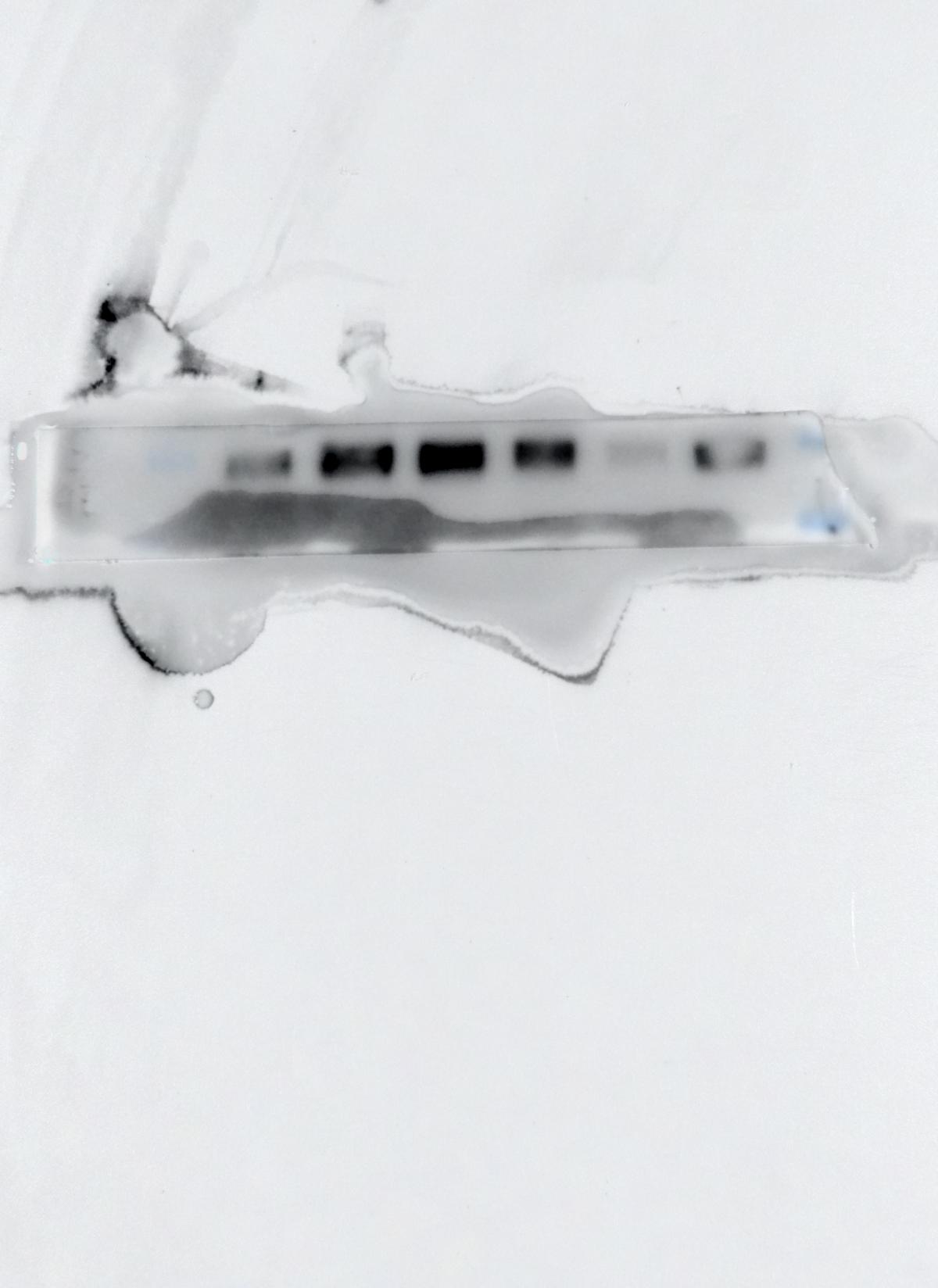


BAD


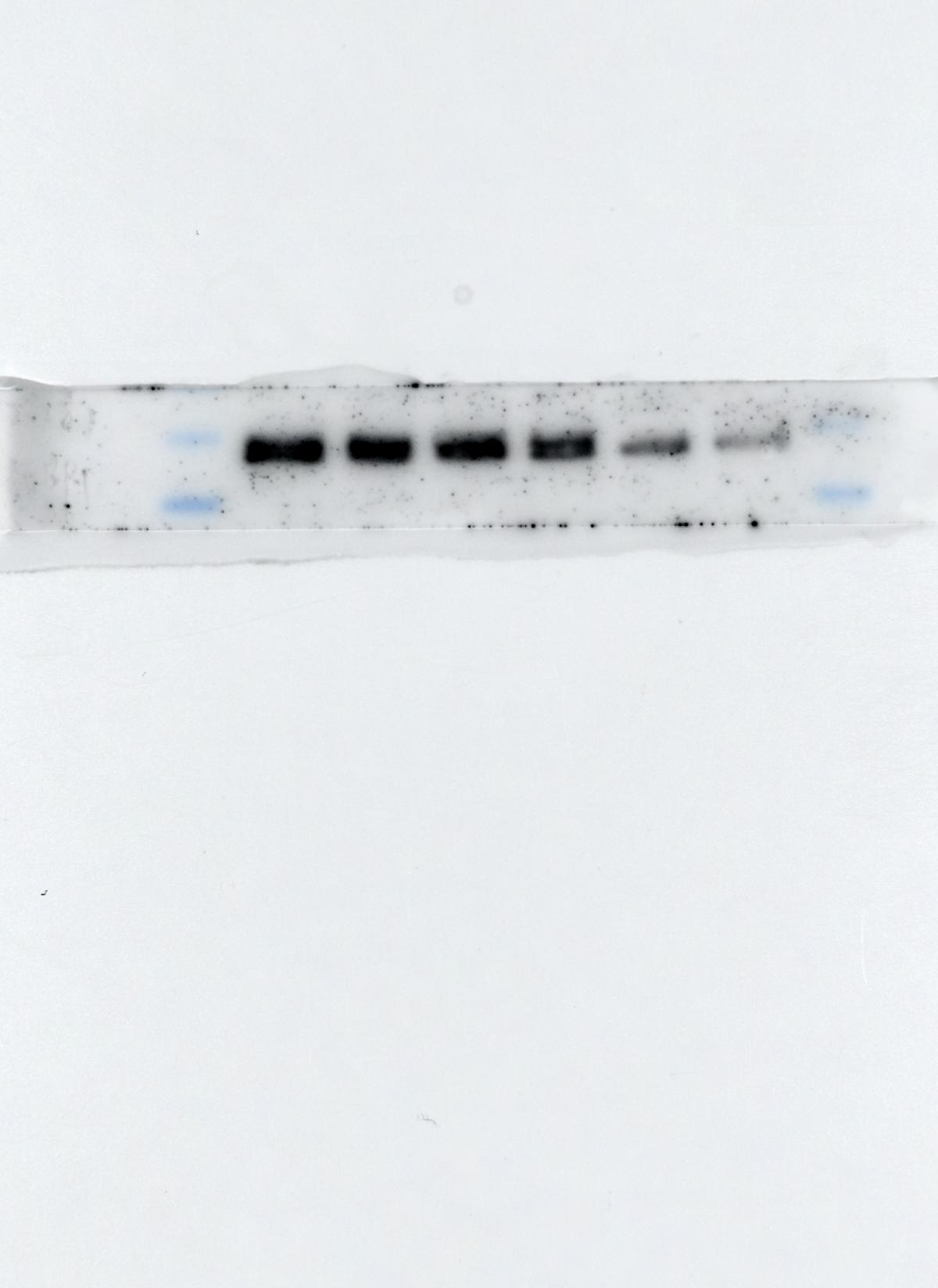


p-BAD


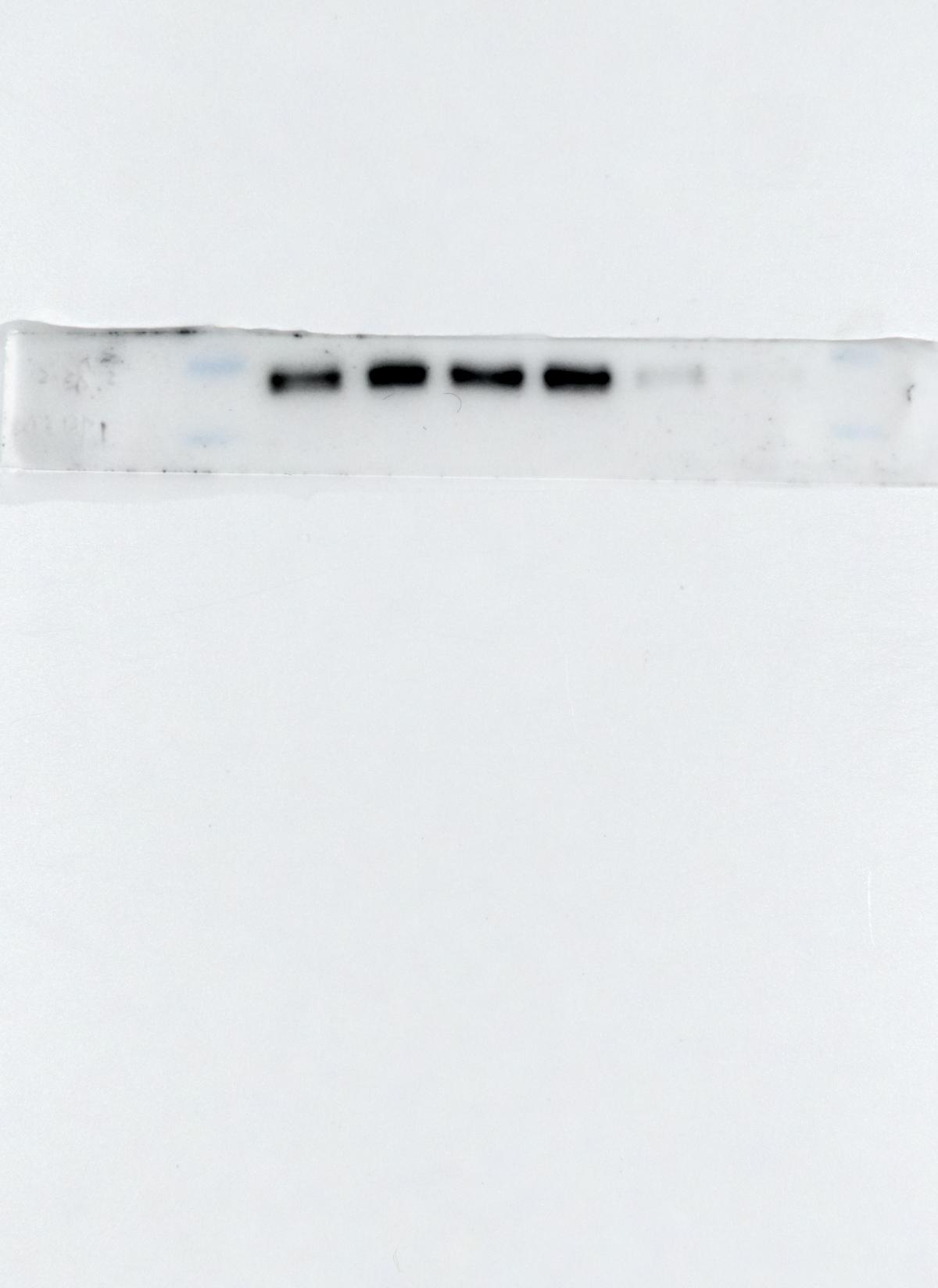


GAPDH


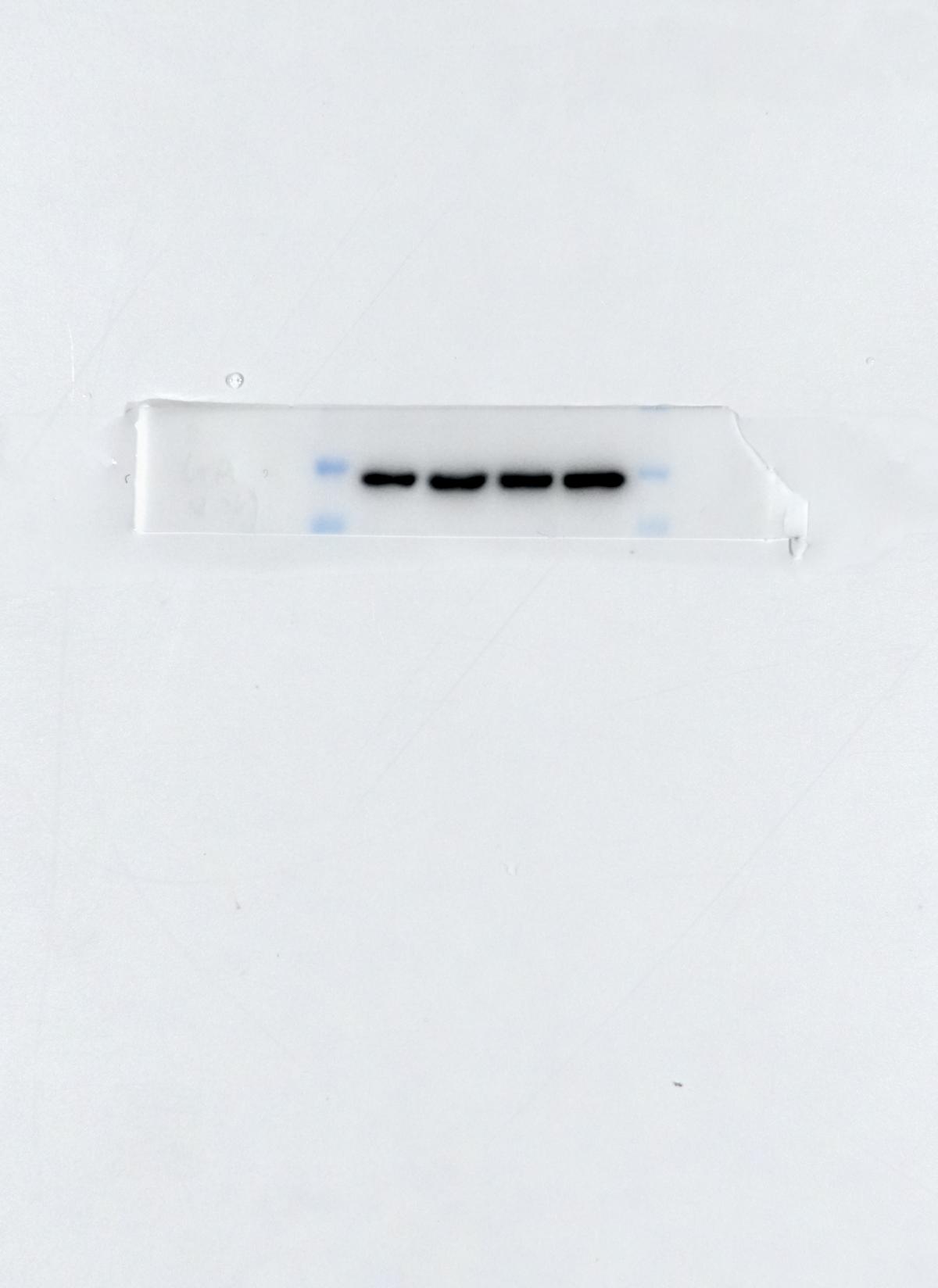


**Figure 4D**

**PI3K inhibitor-Pictilisib**

PI3K


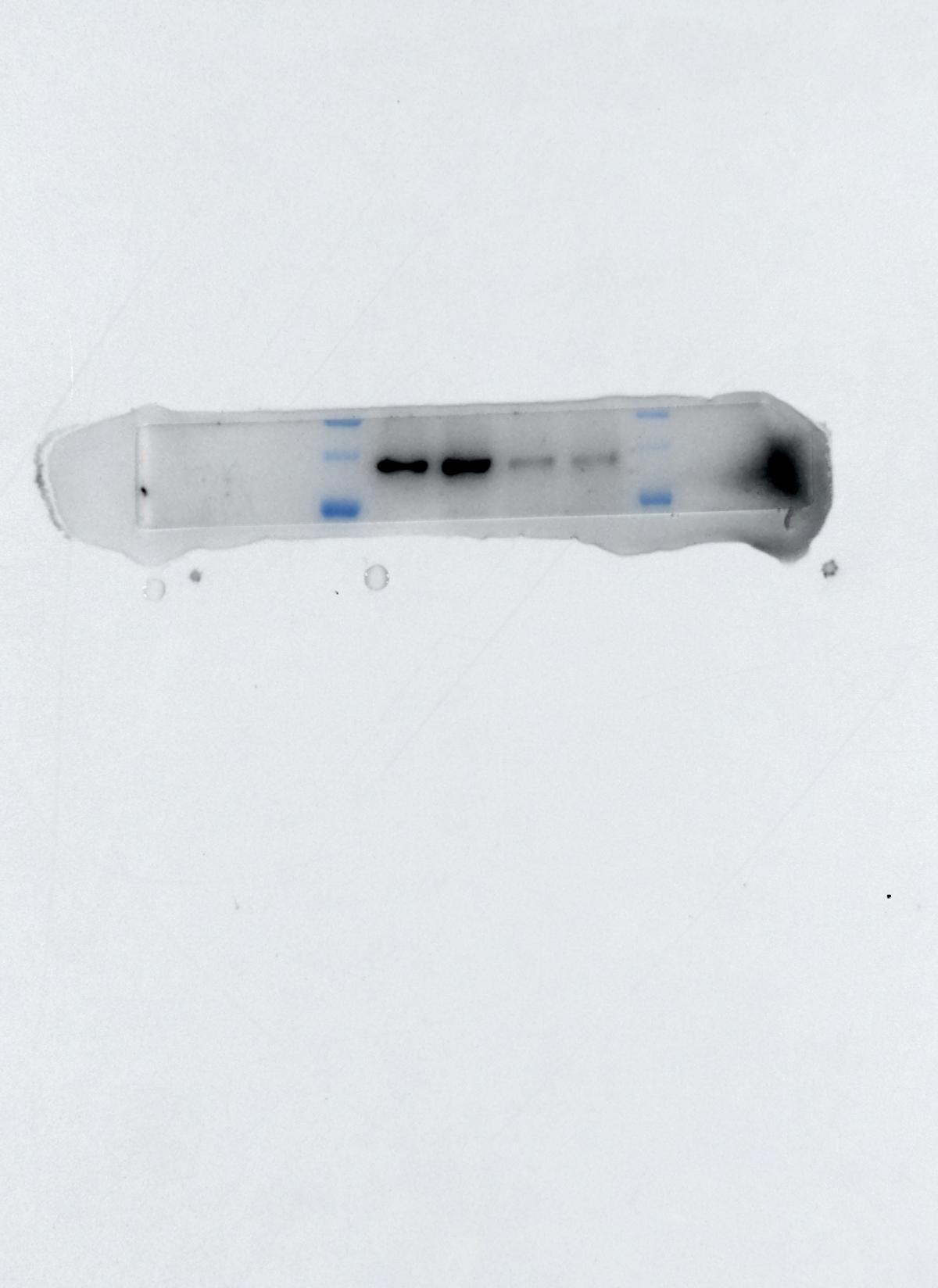


mTOR


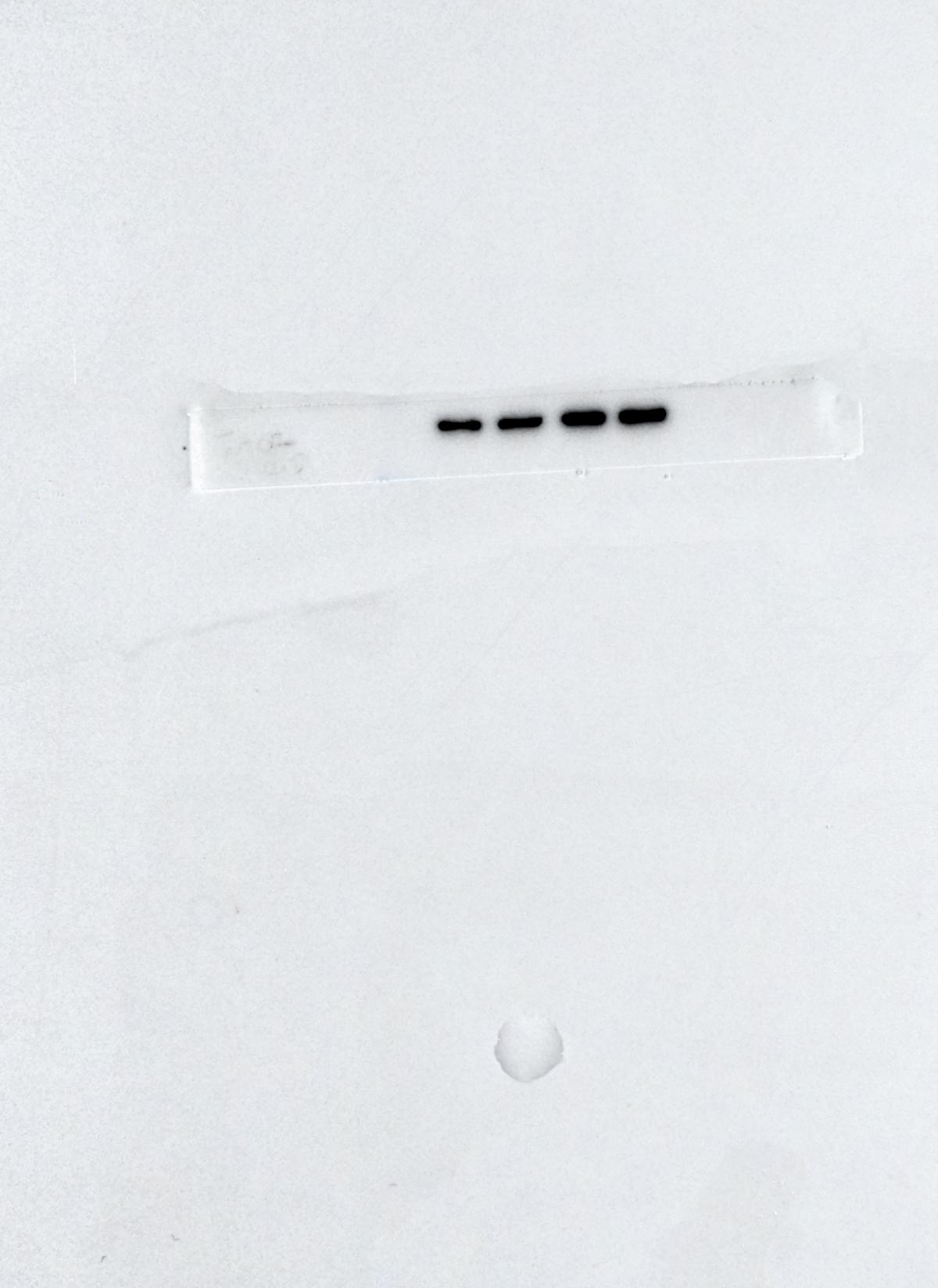


p-mTOR


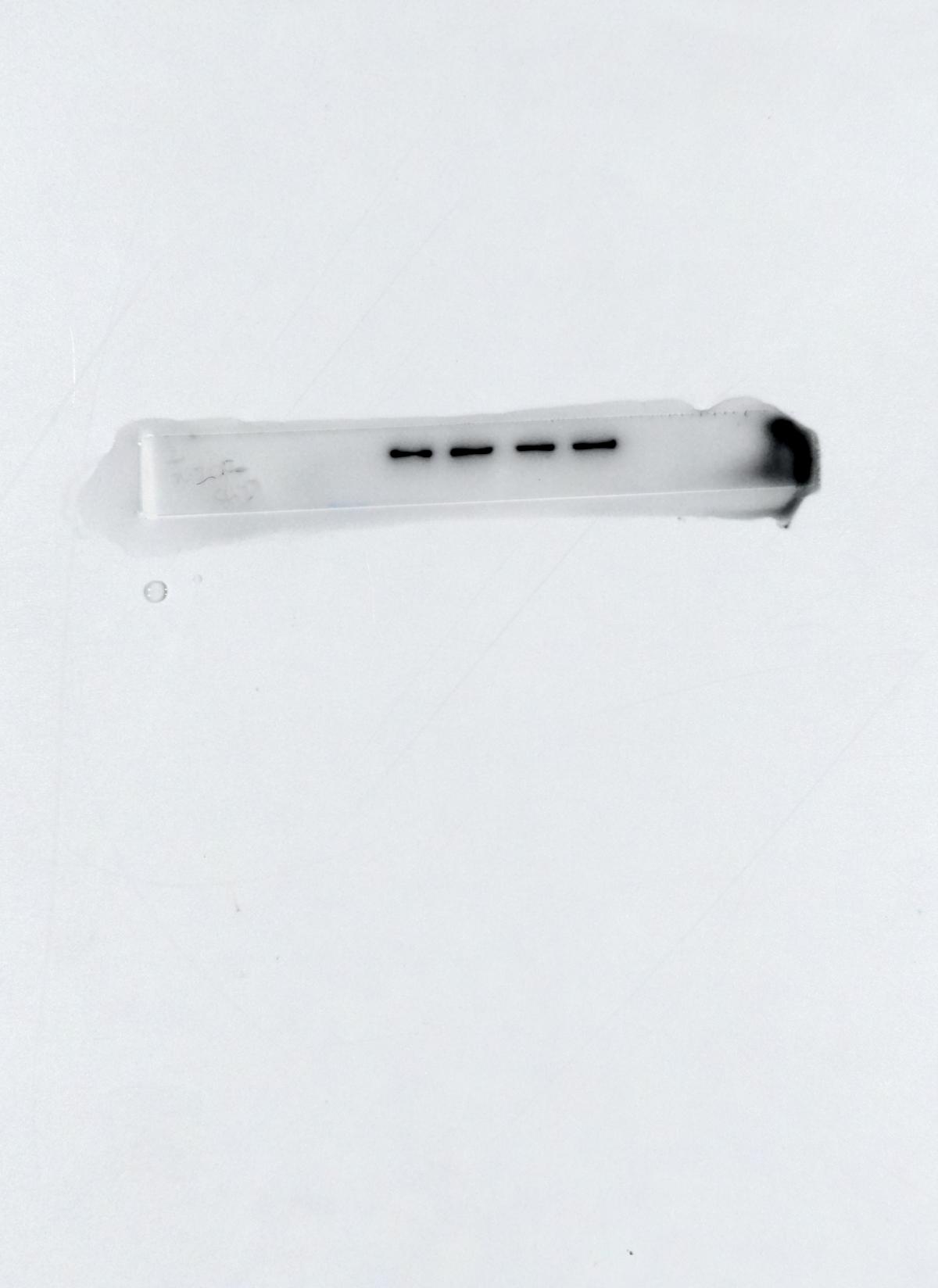


AKT


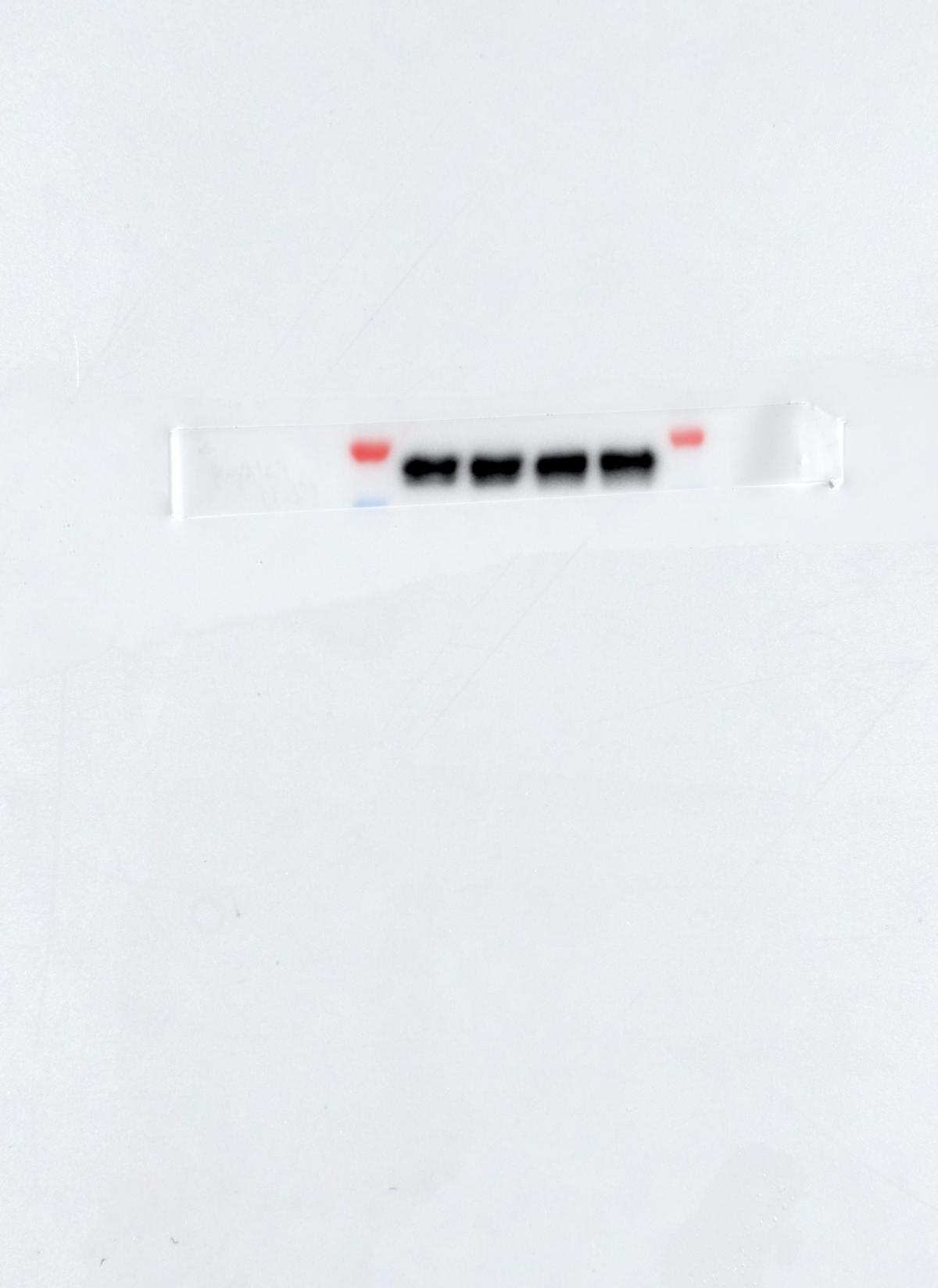


p-AKT


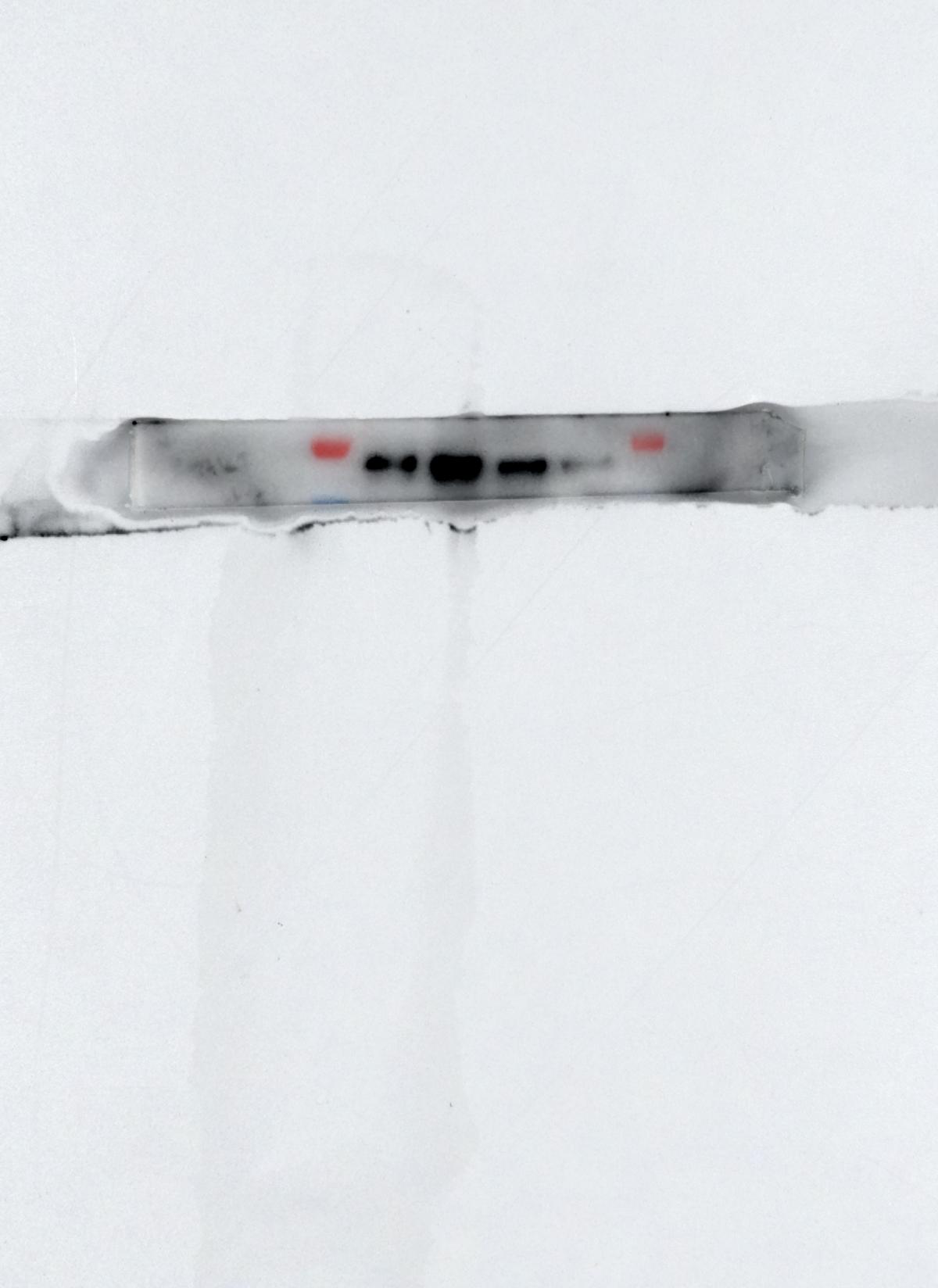


4EBP1


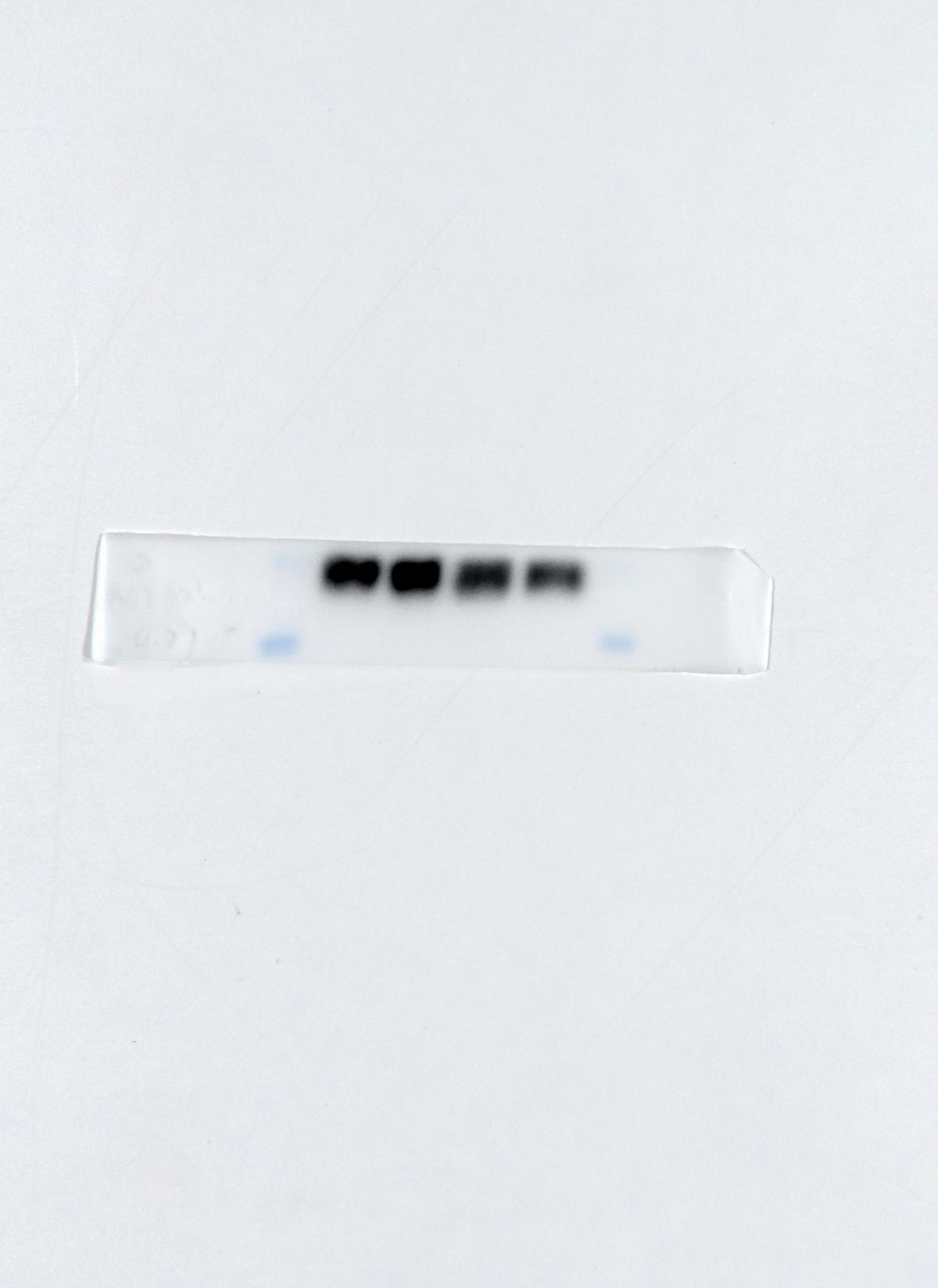


p-4EBP1


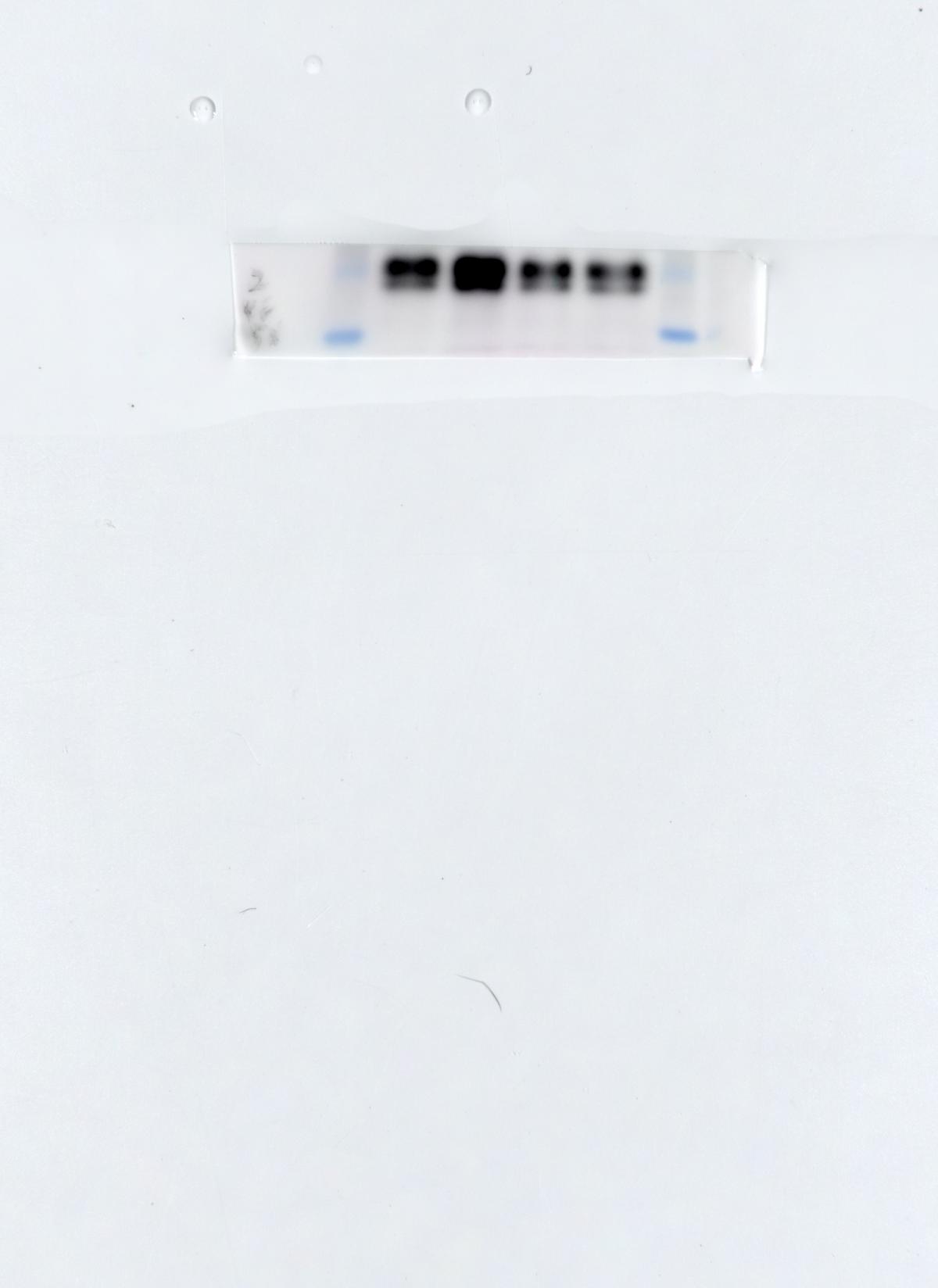


GAPDH


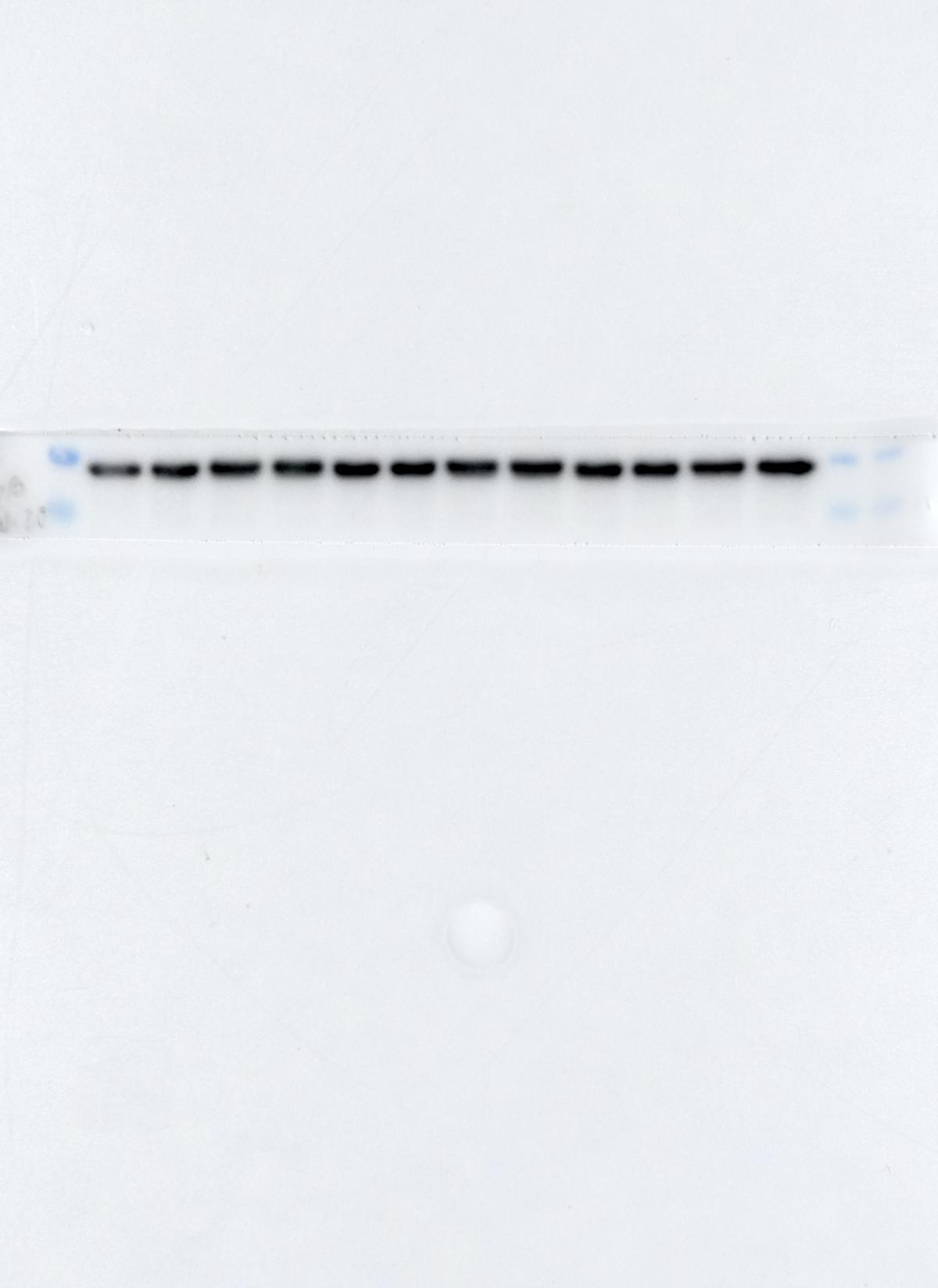


**Figure 4D**

**mTOR inhibitor-Temsirolimus**

mTOR


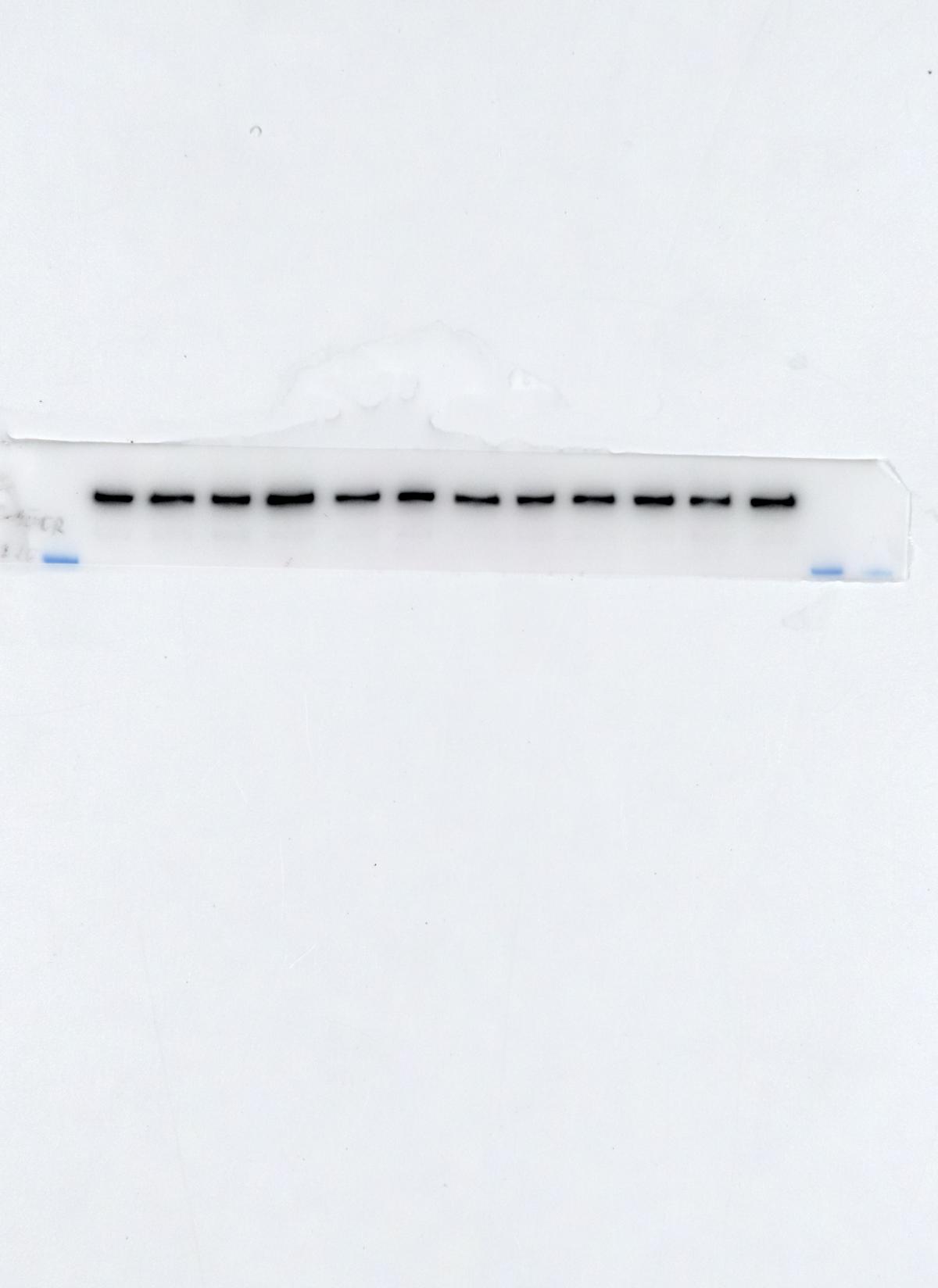


p-mTOR


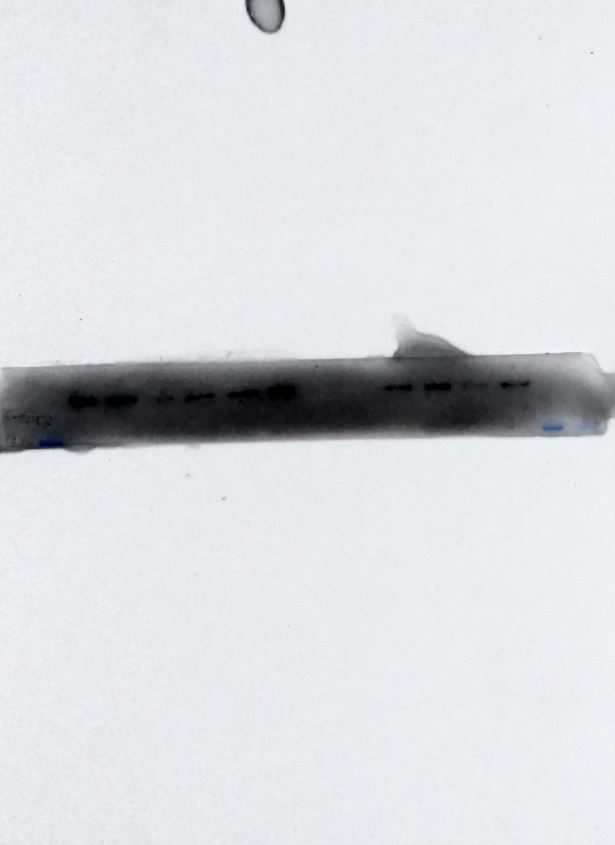

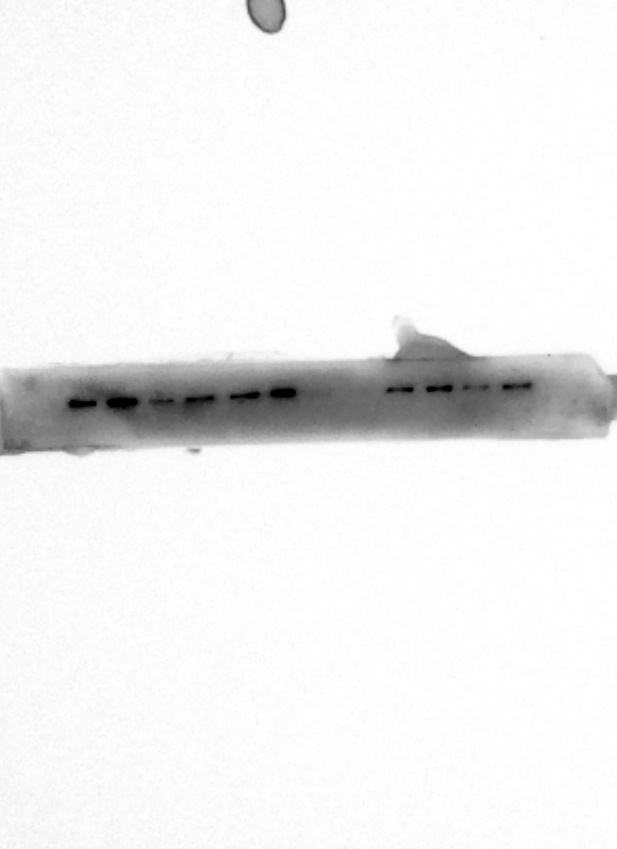


without marker

with marker

4EBP1


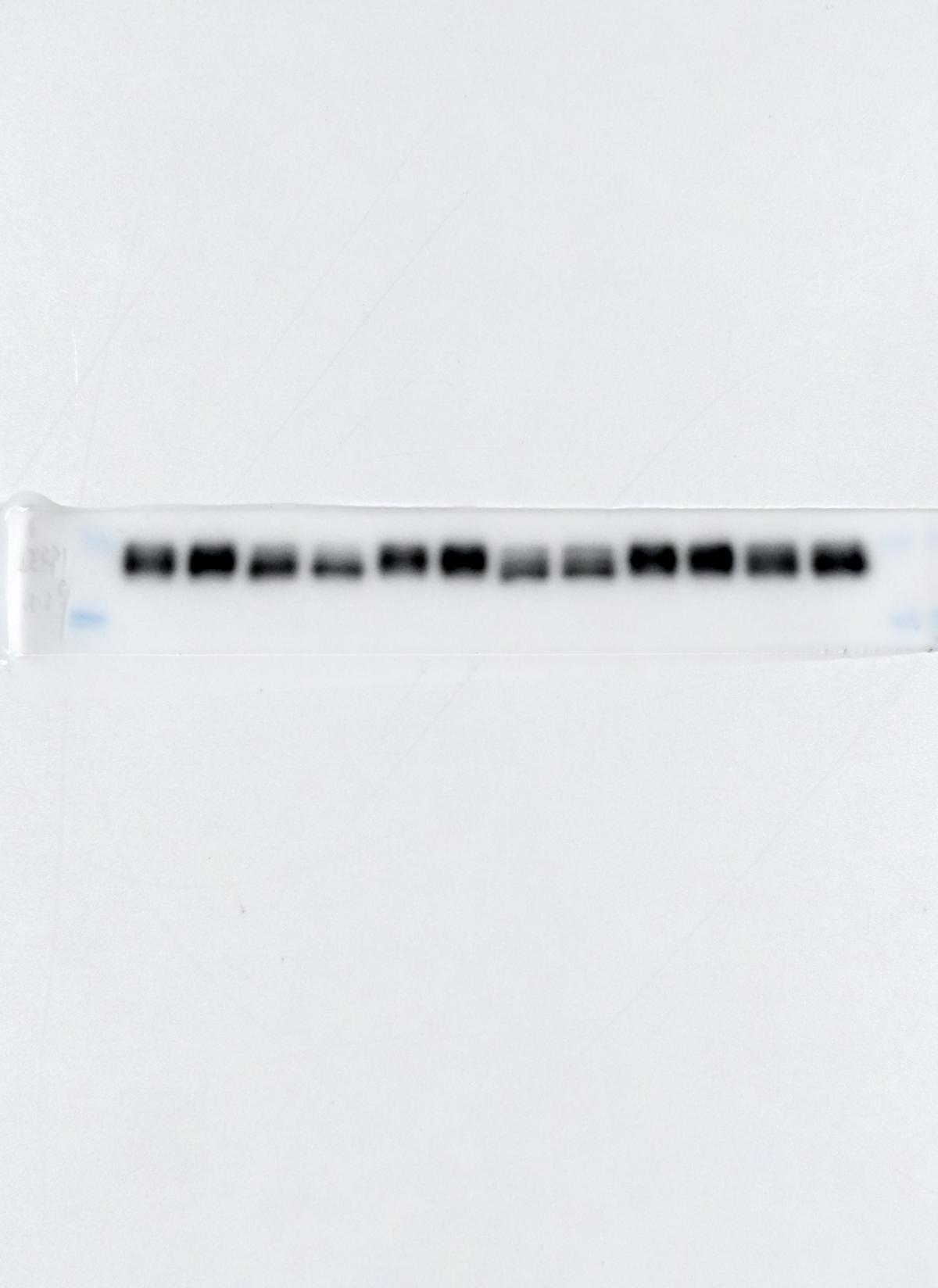


p-4EBP1


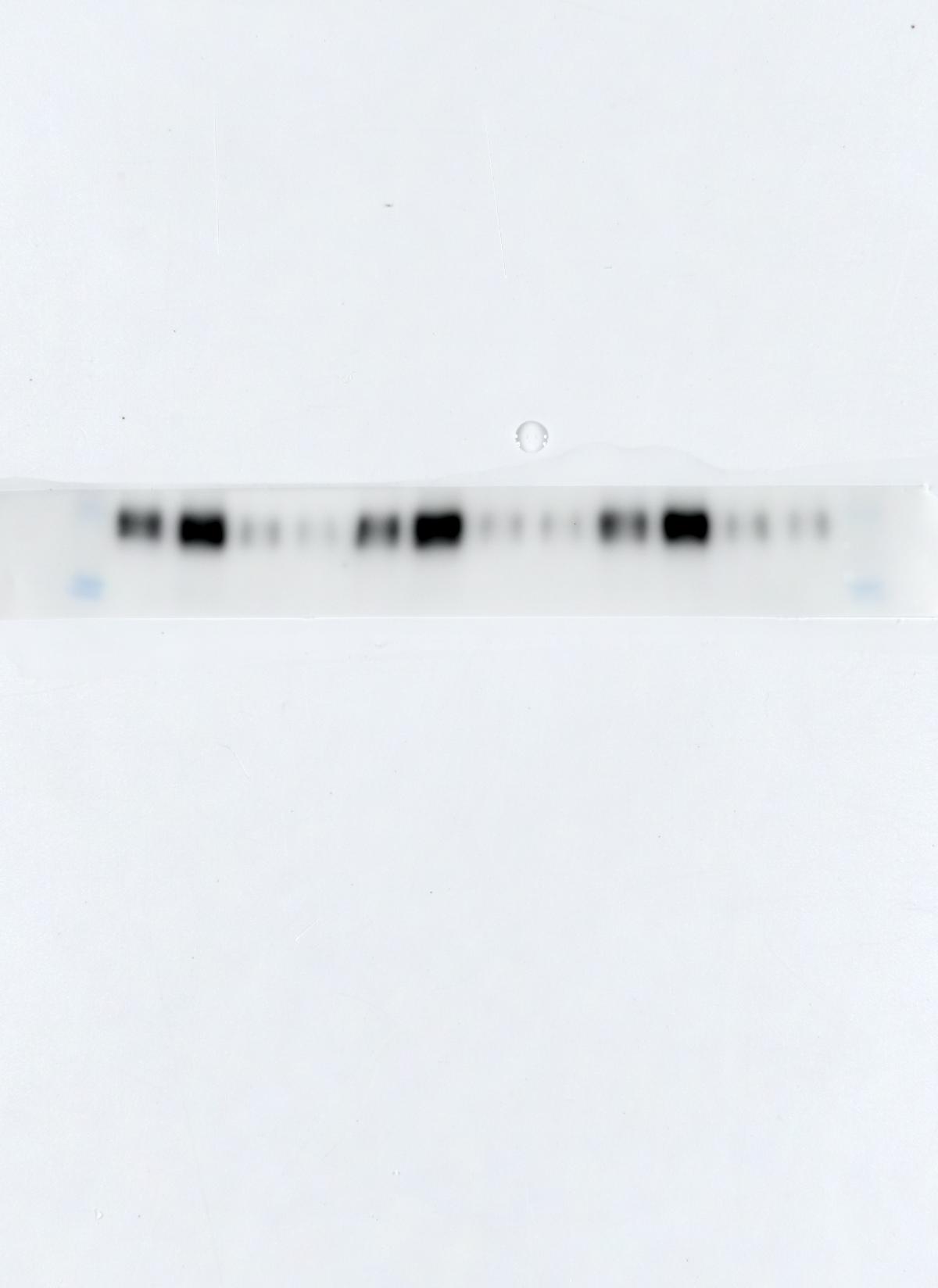


GAPDH


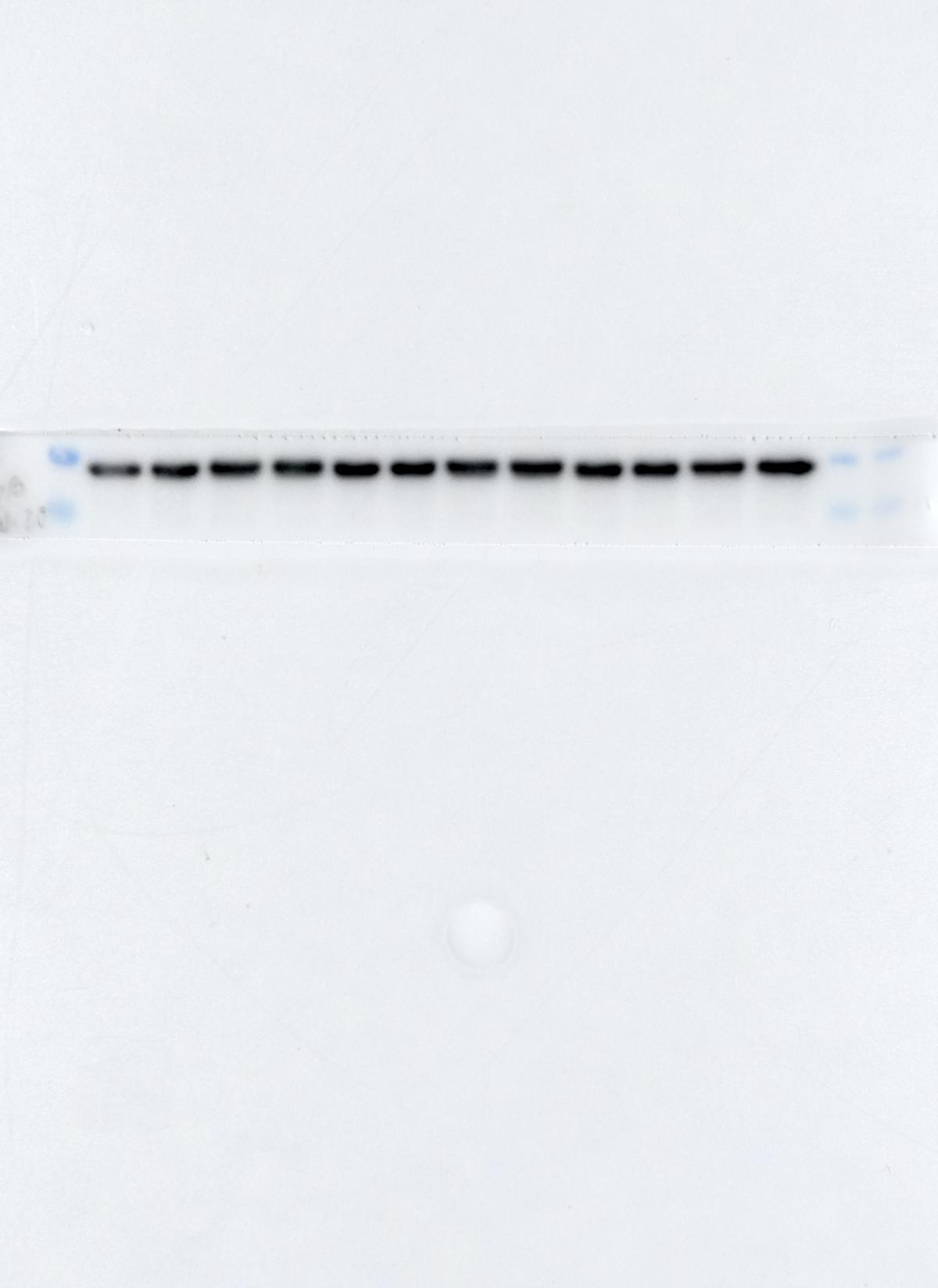


**Figure 4F**

**AKT inhibitor-MK2206**

mTOR


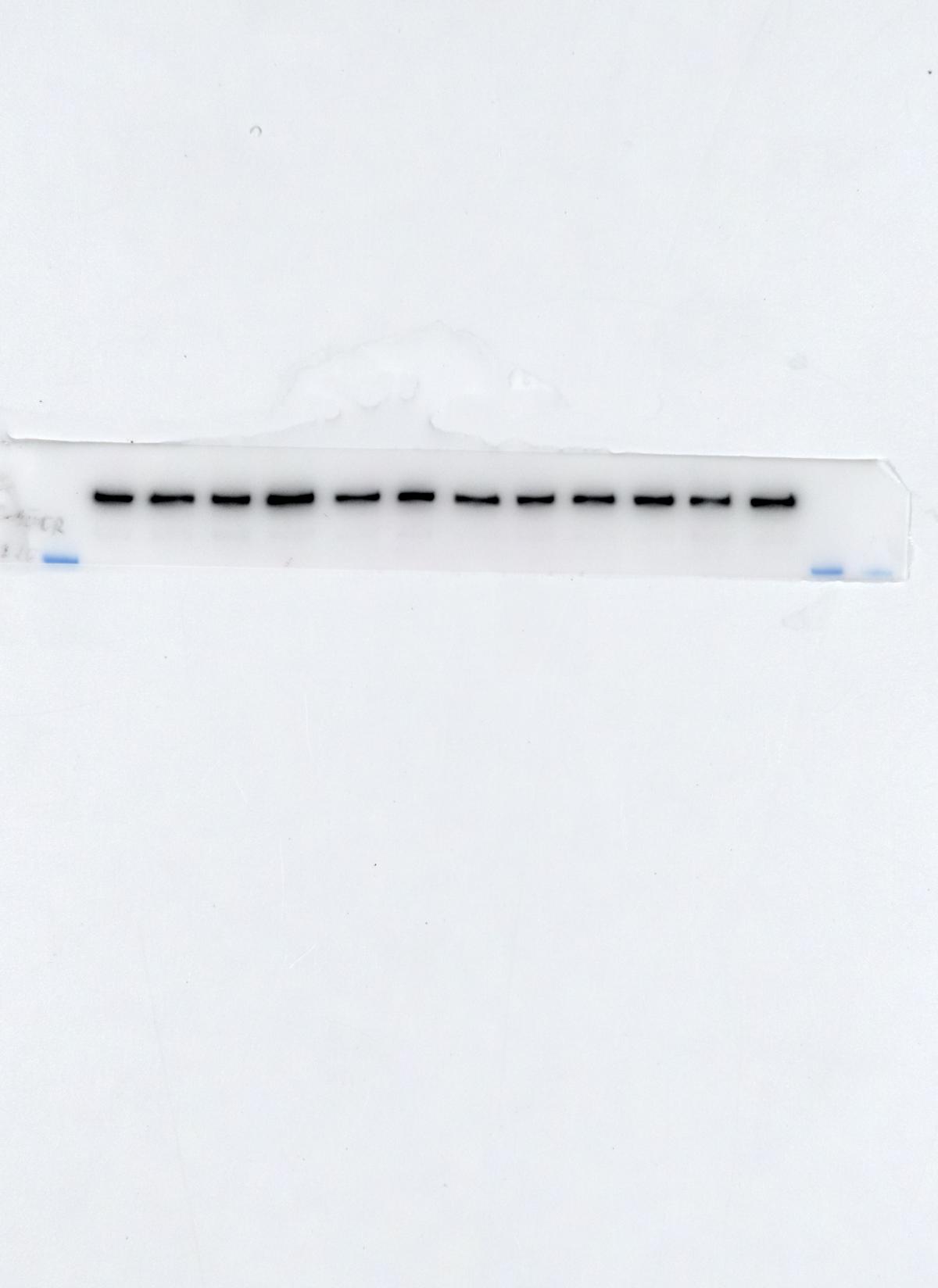


p-mTOR


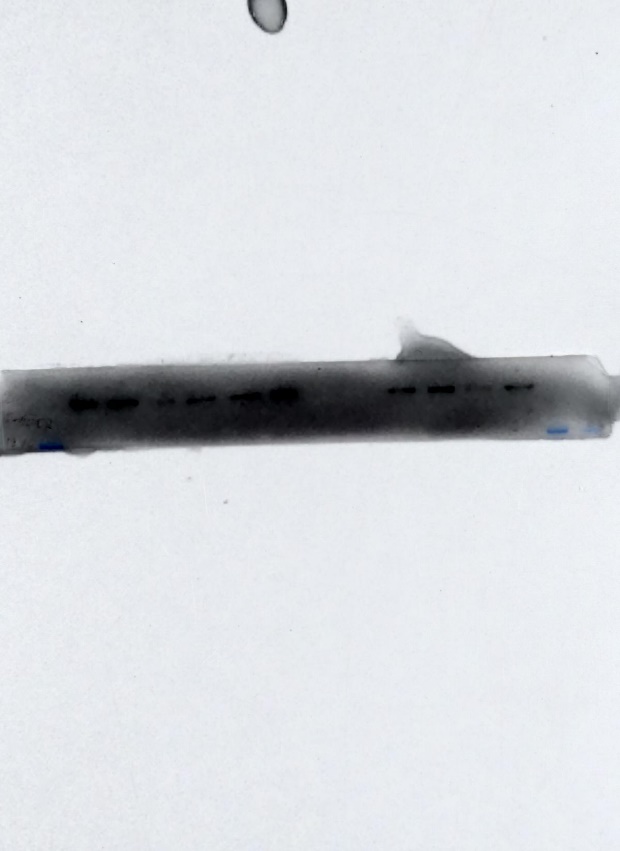

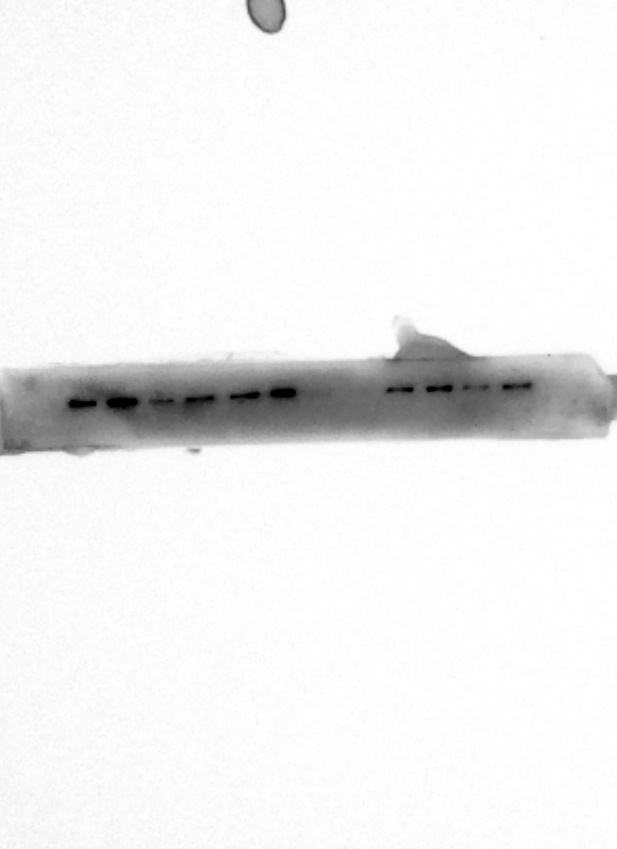


without marker

with marker

AKT


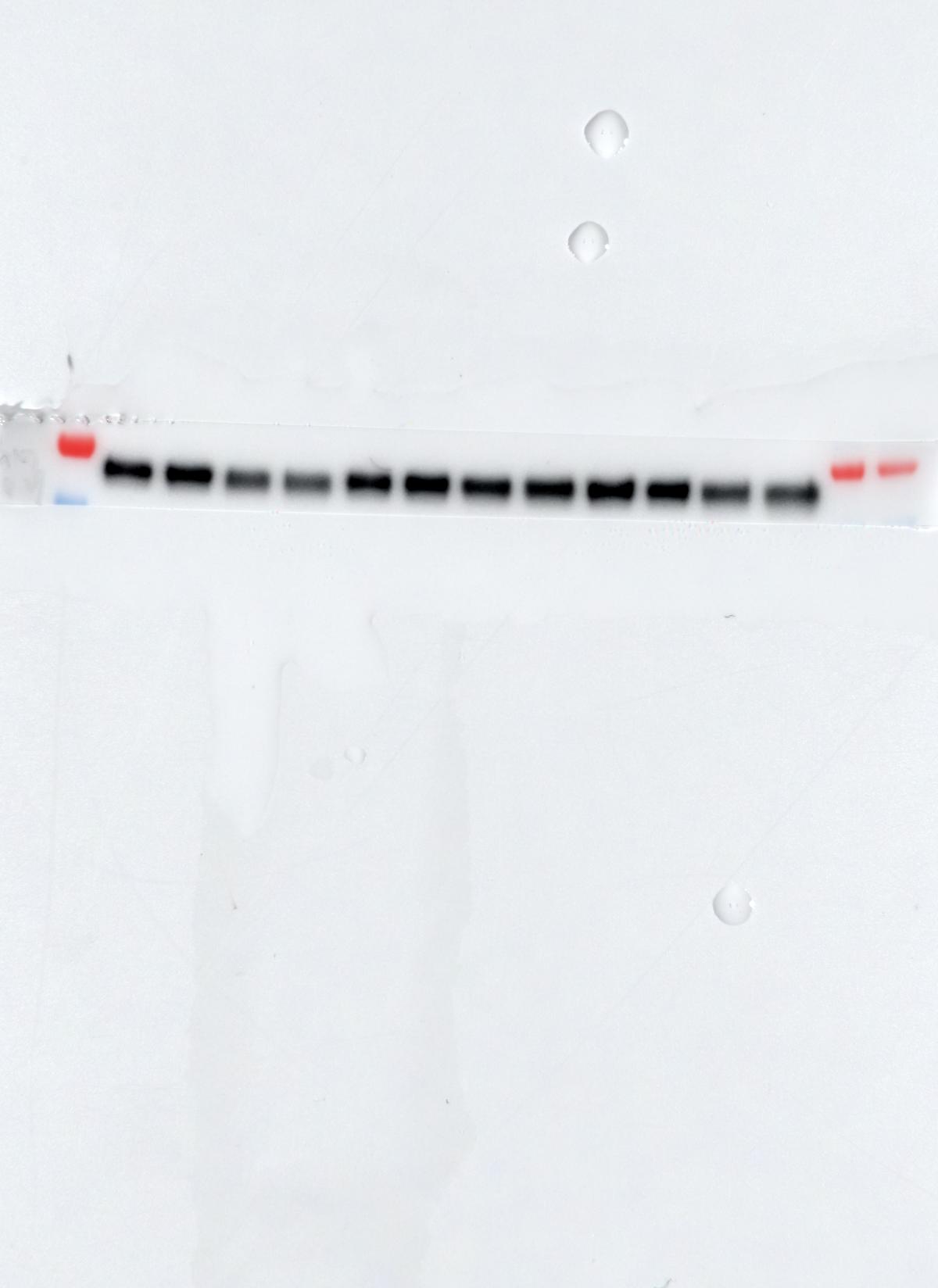


p-AKT


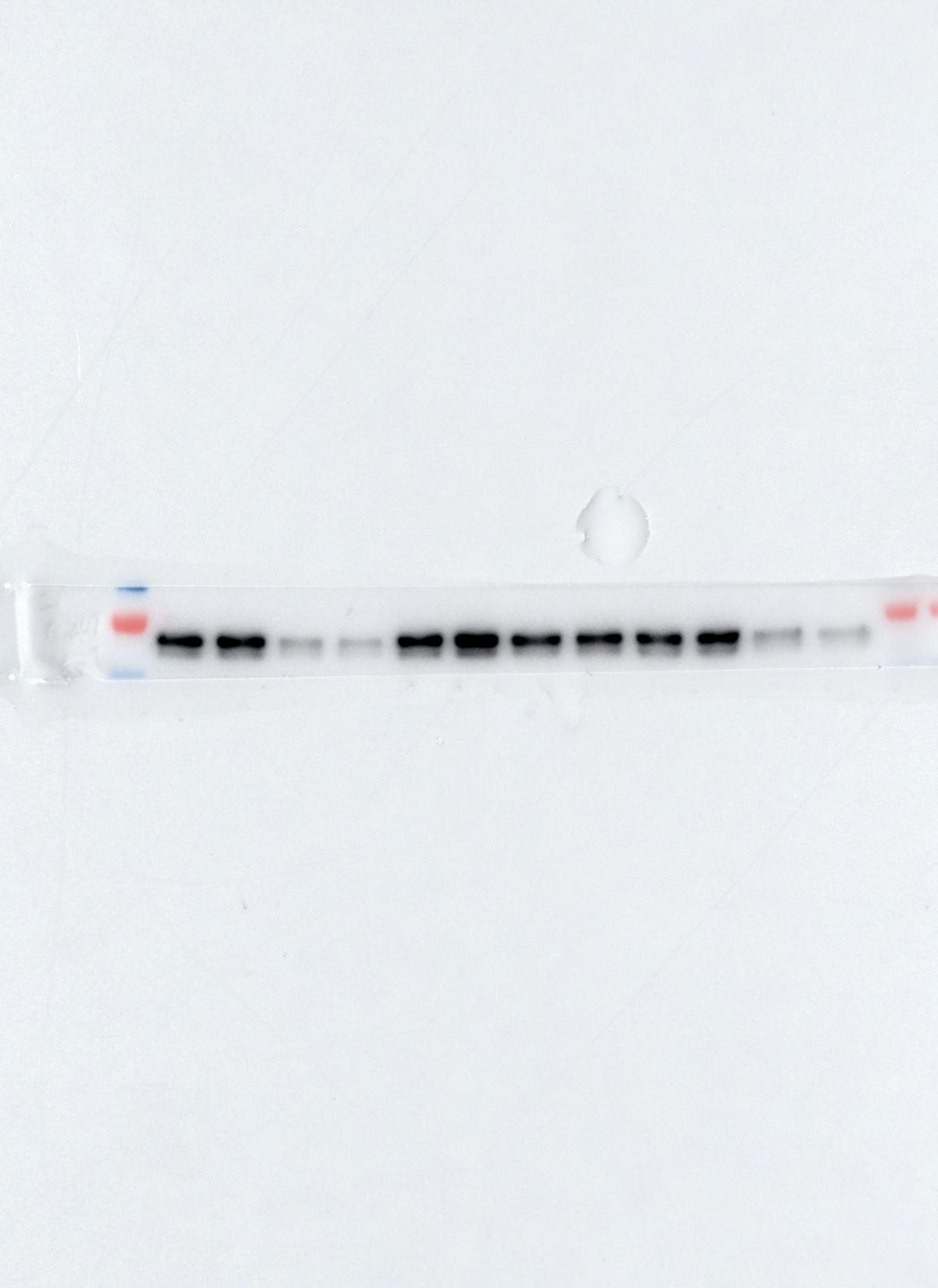


4EBP1


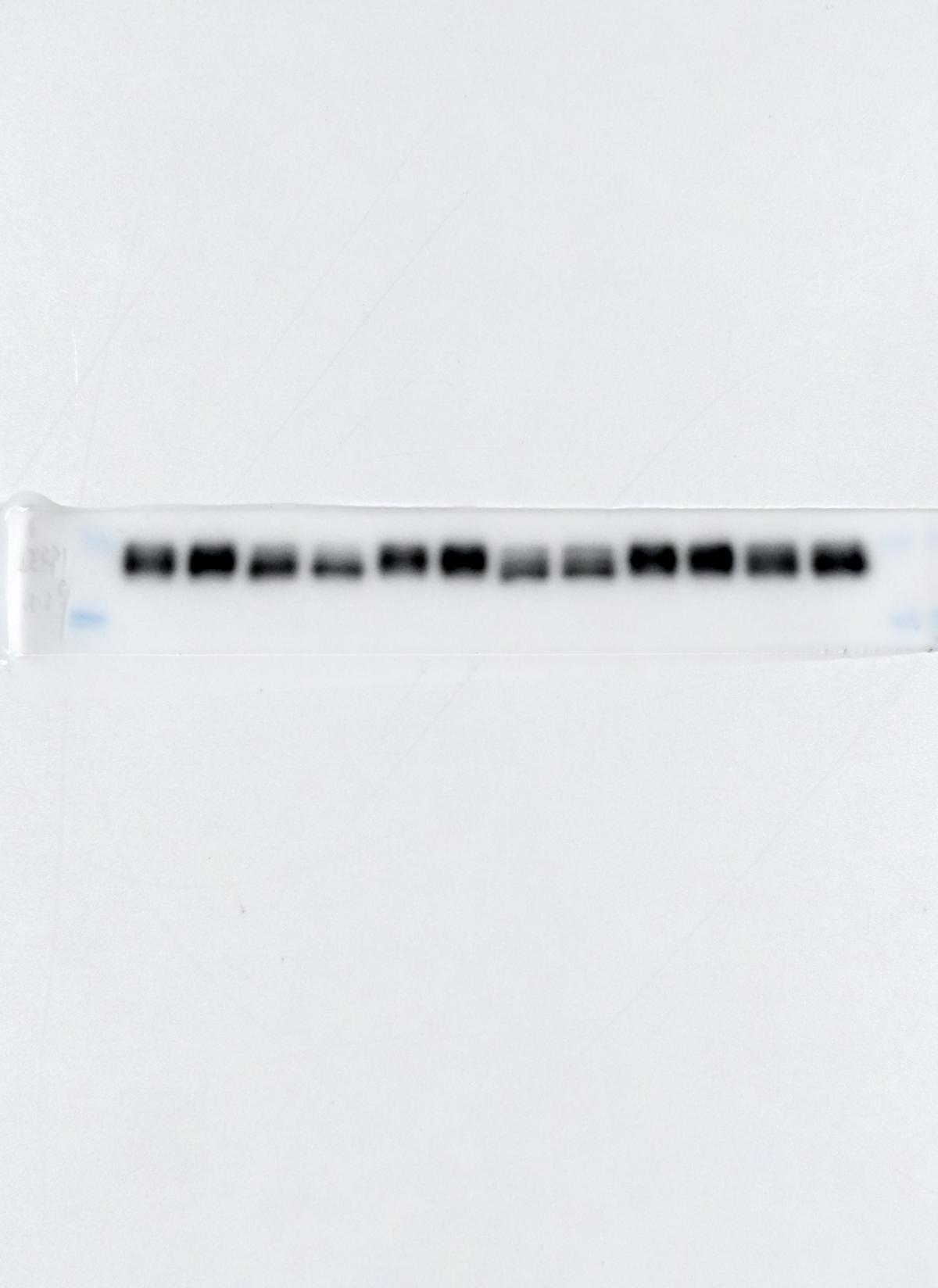


p-4EBP1


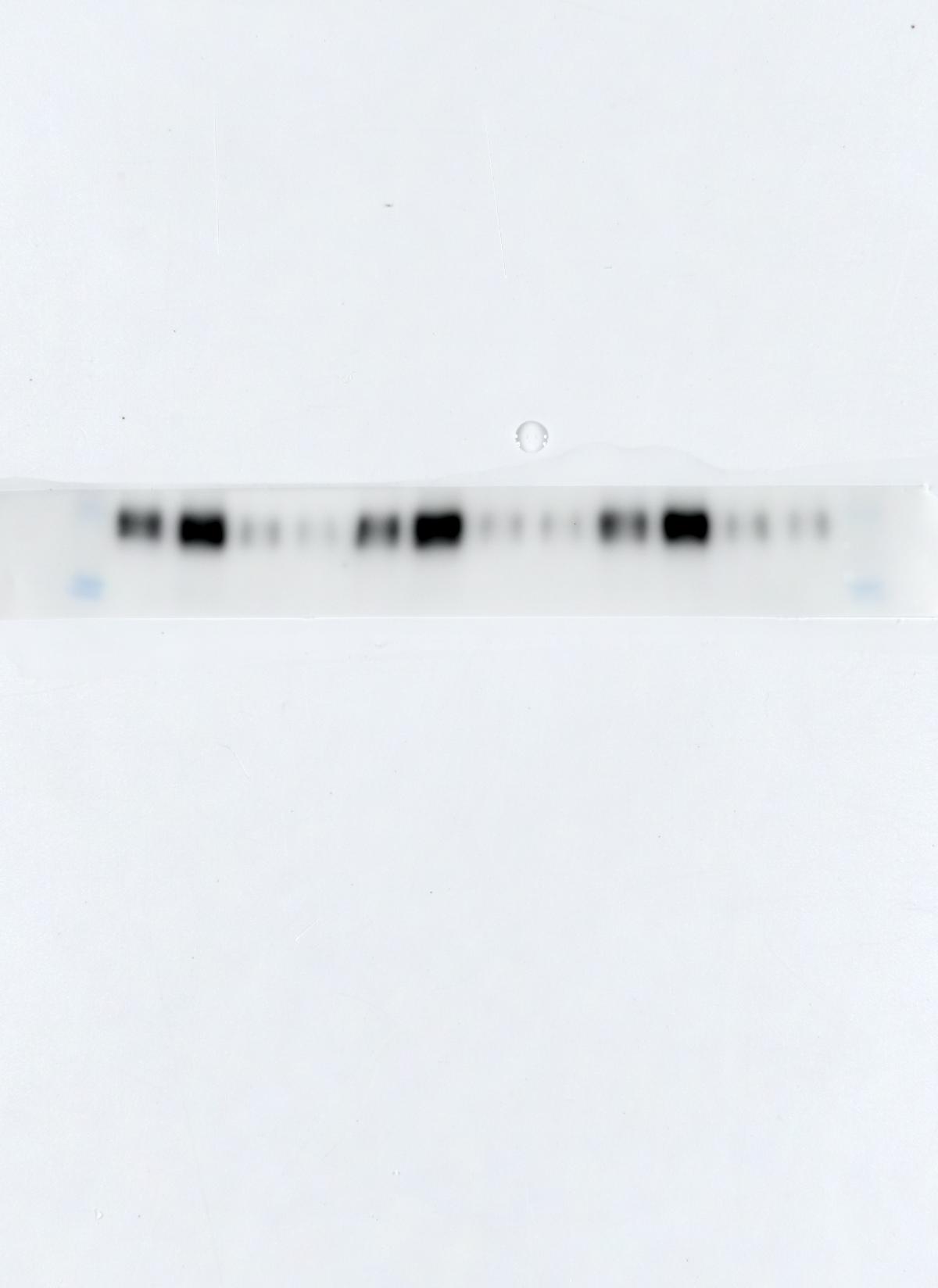


GAPDH

**Figure 5B**

**1: Control-1**

**2: Semaphorin 5A-1**

**3: Control-2**

**4: Semaphorin 5A-2**

**5: Control-3**

**6: Semaphorin 5A-3**


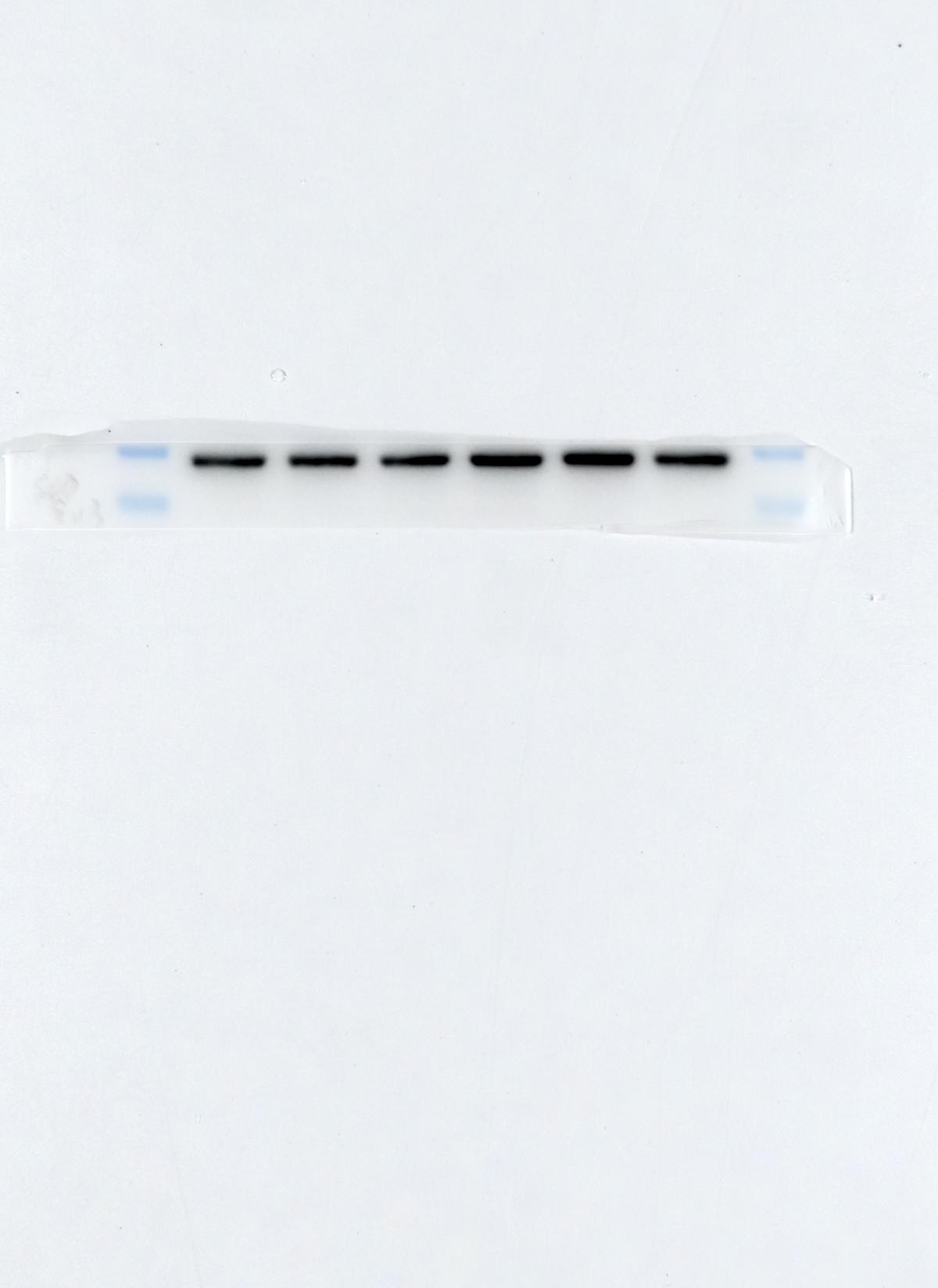


1 2 3 4 5 6

GPX4


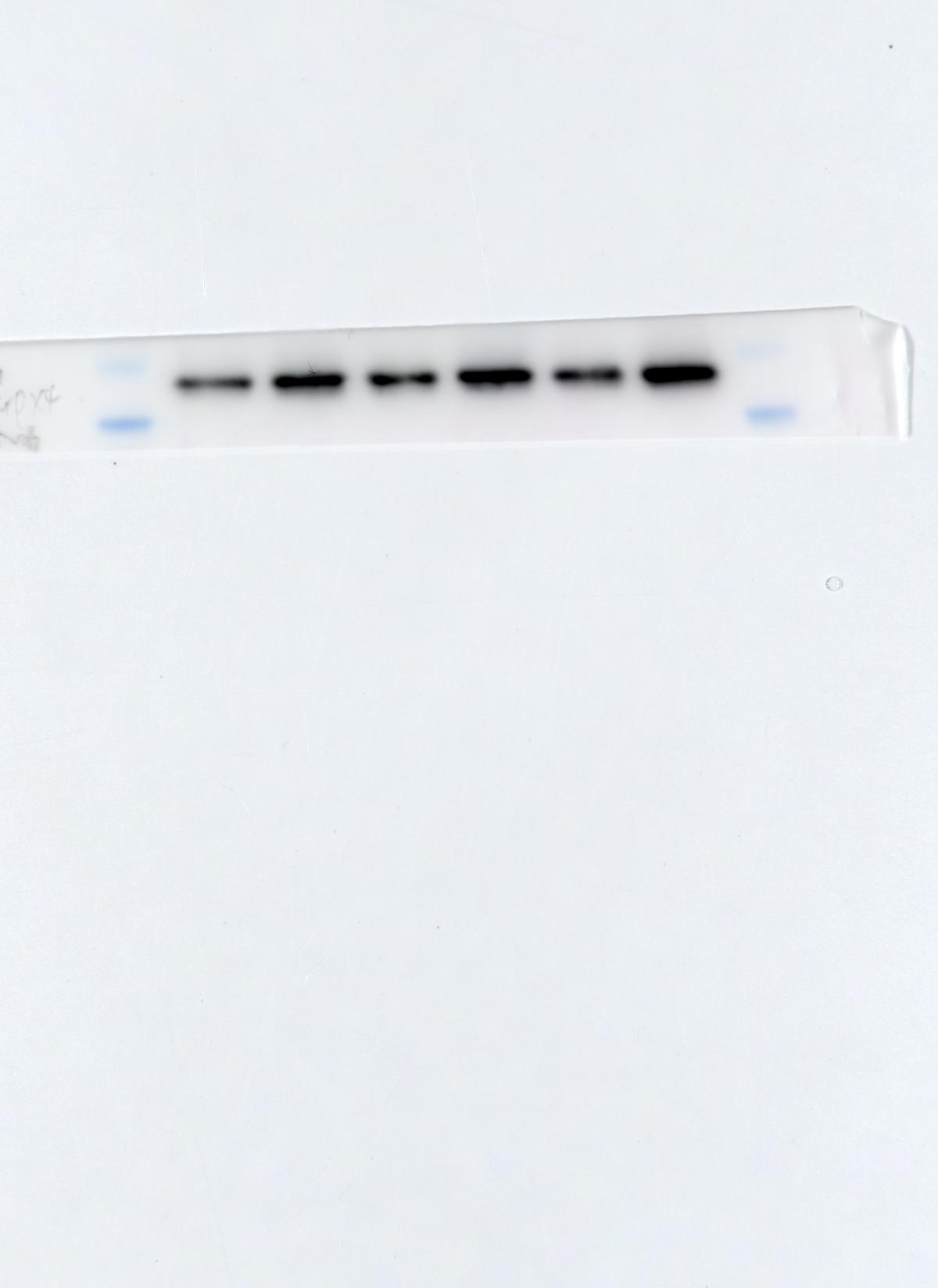


GAPDH


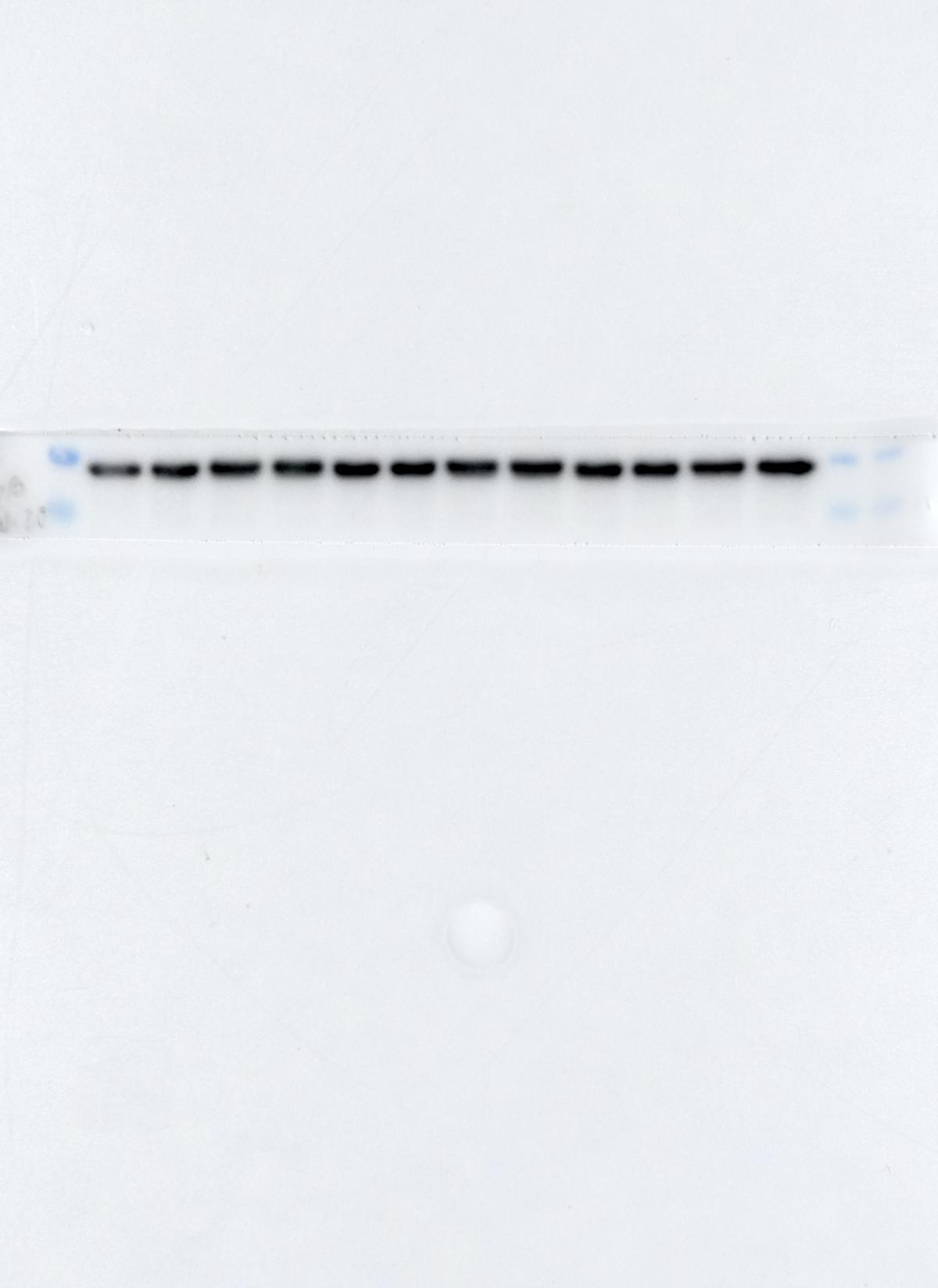


**Figure 5I**

GPX4


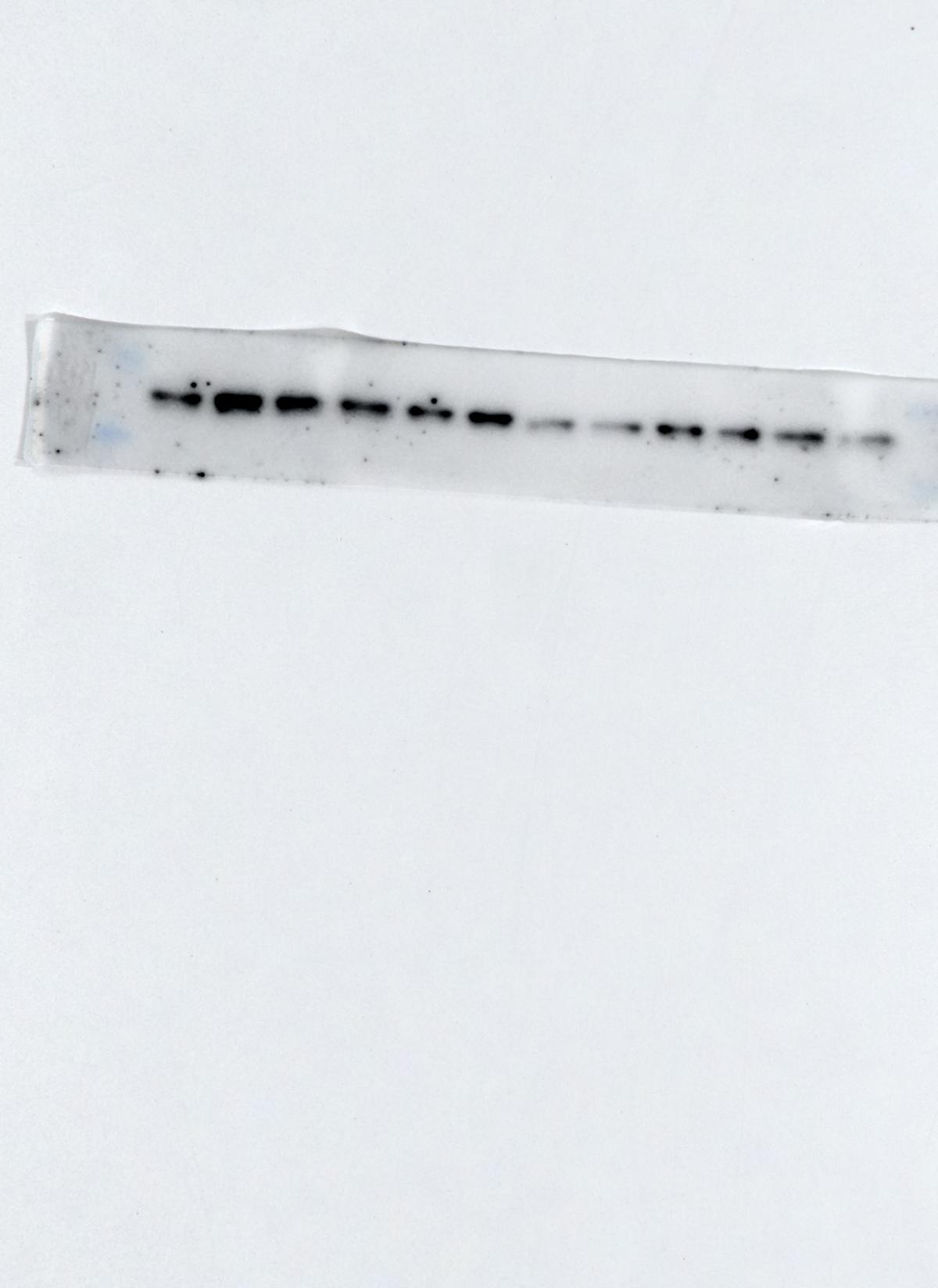


GAPDH


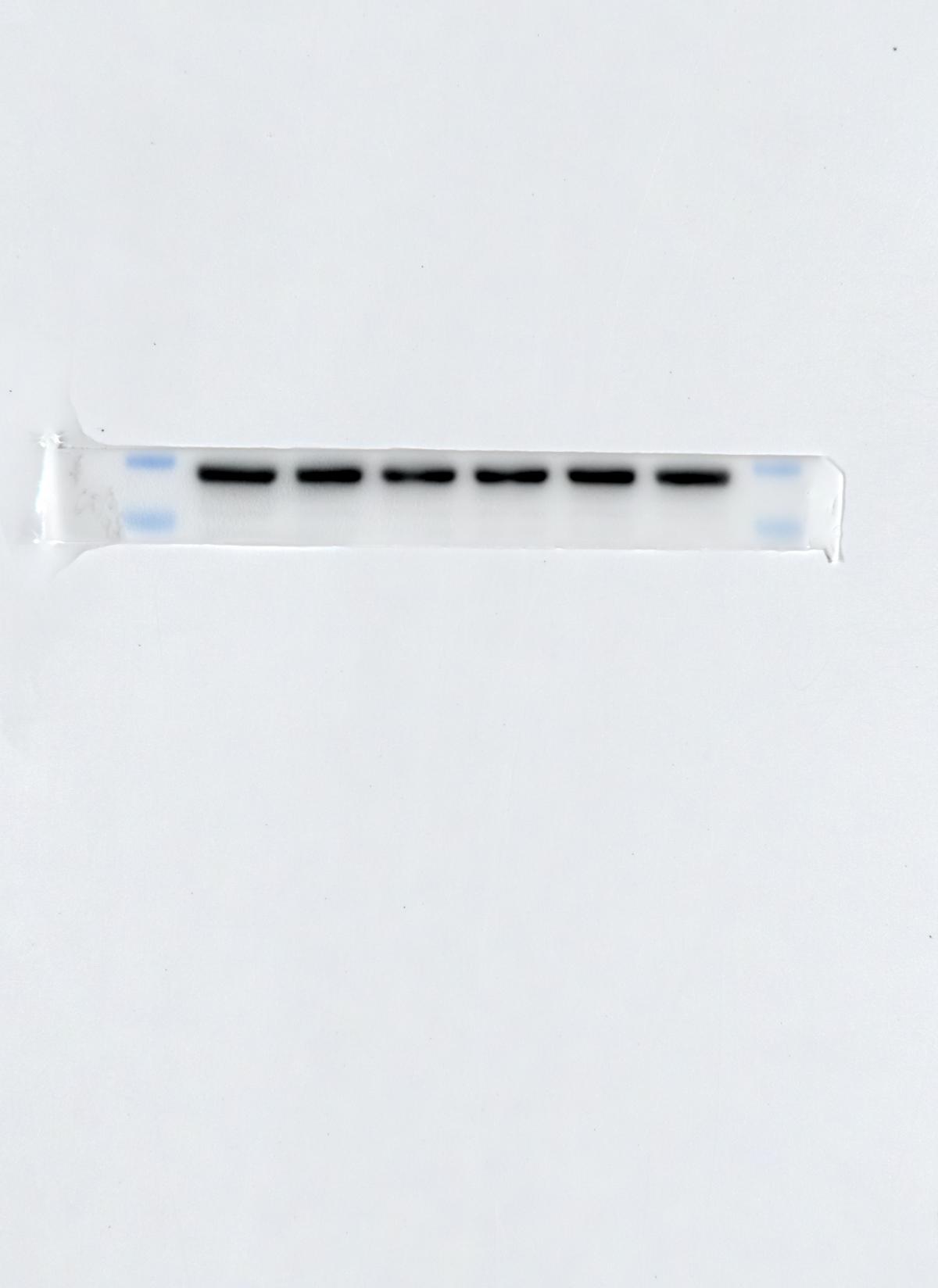


1 2 3 4 5 6

**Figure 6C**

**1: Control-1**

**2: Semaphorin 5A-1**

**3: Control-2**

**4: Semaphorin 5A-2**

**5: Control-3**

**6: Semaphorin 5A-3**

SCD-1


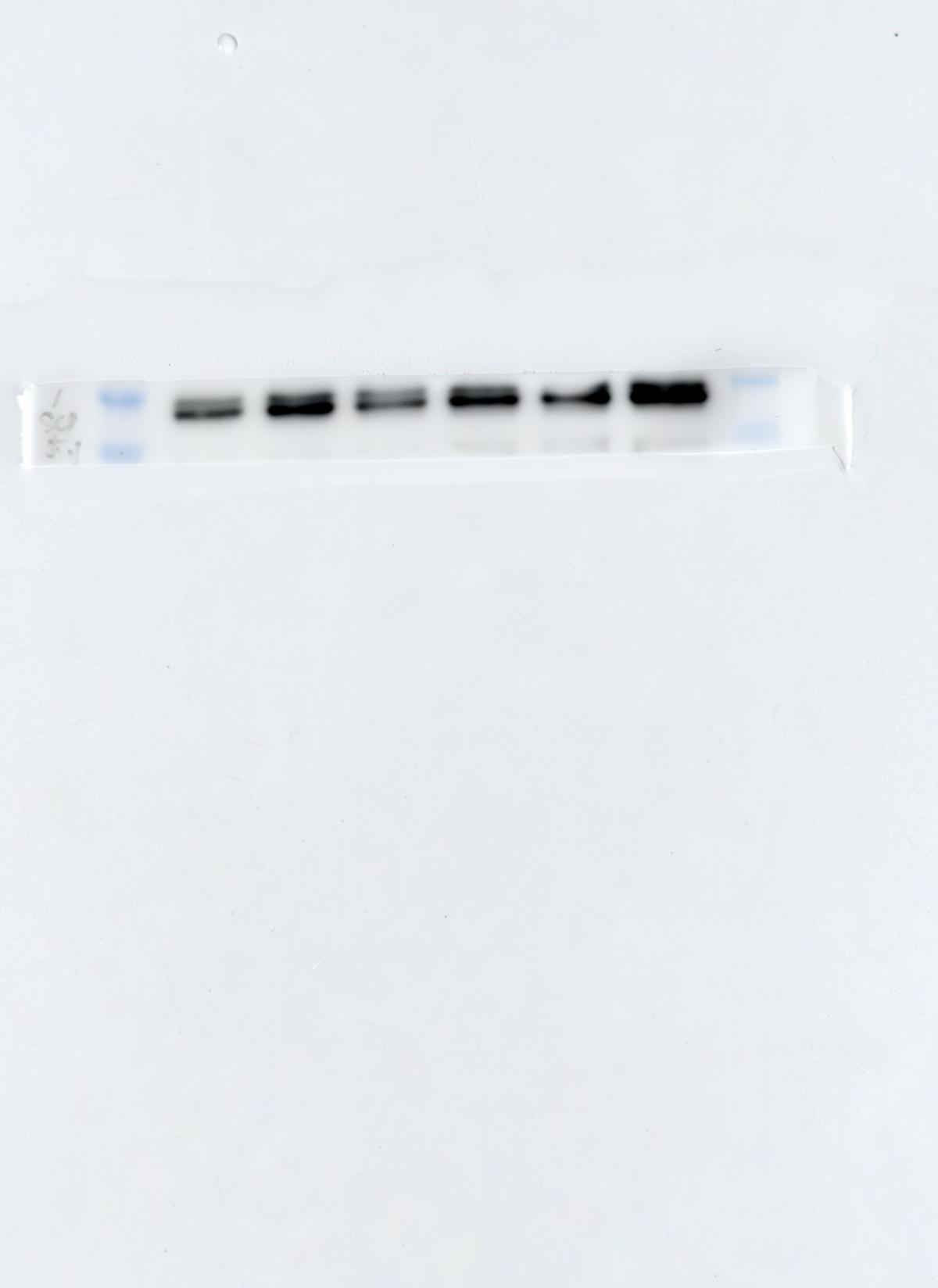


SREBP1


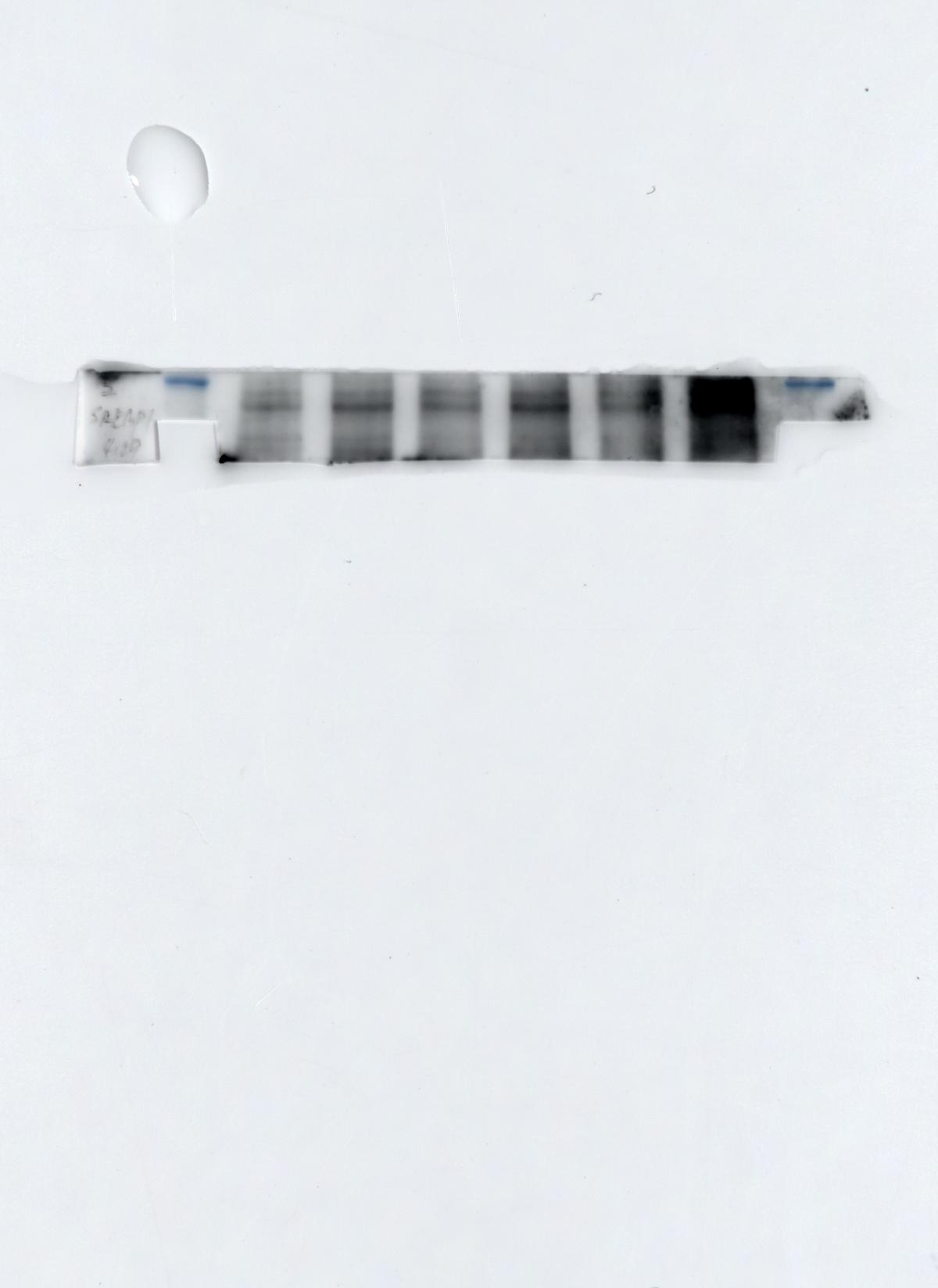


GAPDH


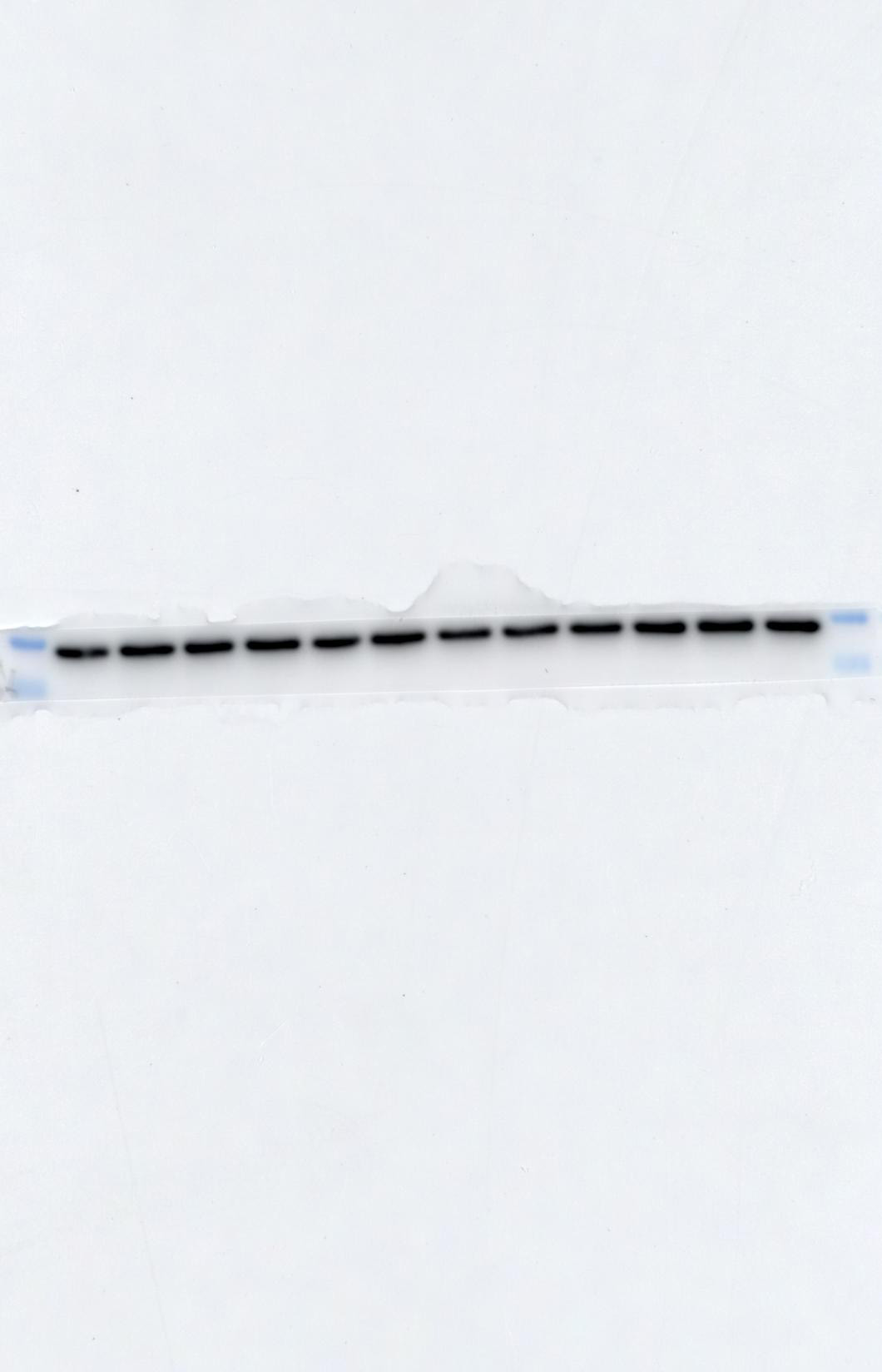


**Figure 6F**

SCD-1


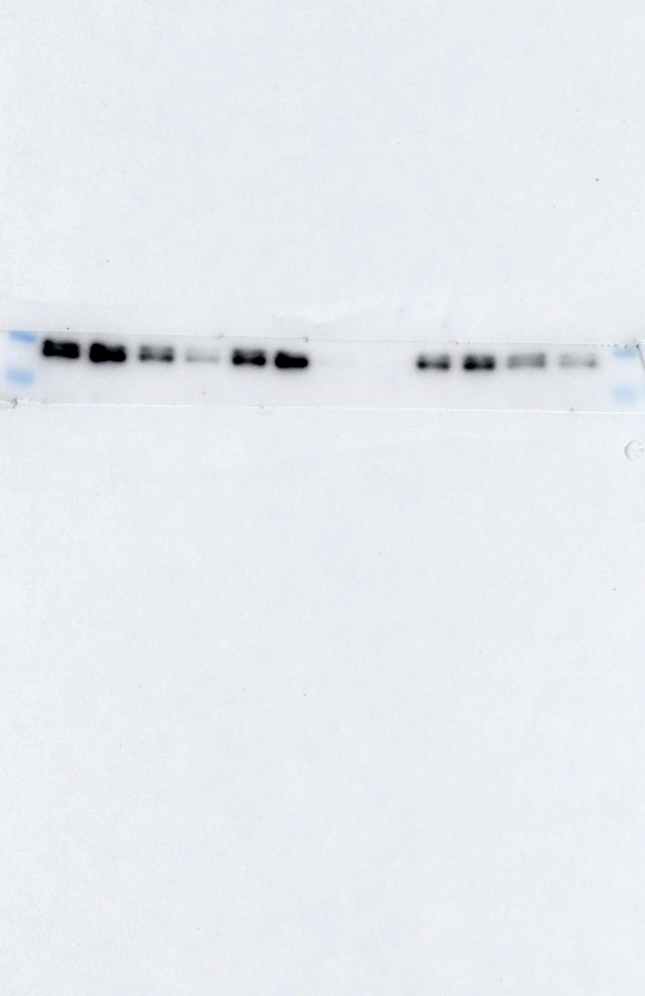


SREBP1


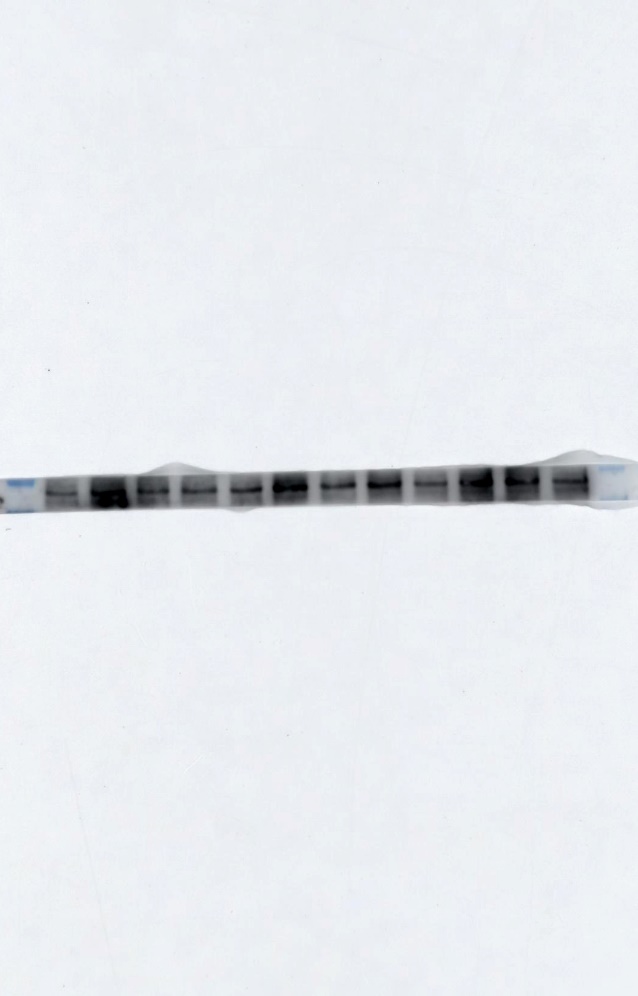


130kDa

100kDa

GAPDH

**Figure 6G**

**1: si NC**

**2: si SREBP1-1**

**3: si SREBP1-2**


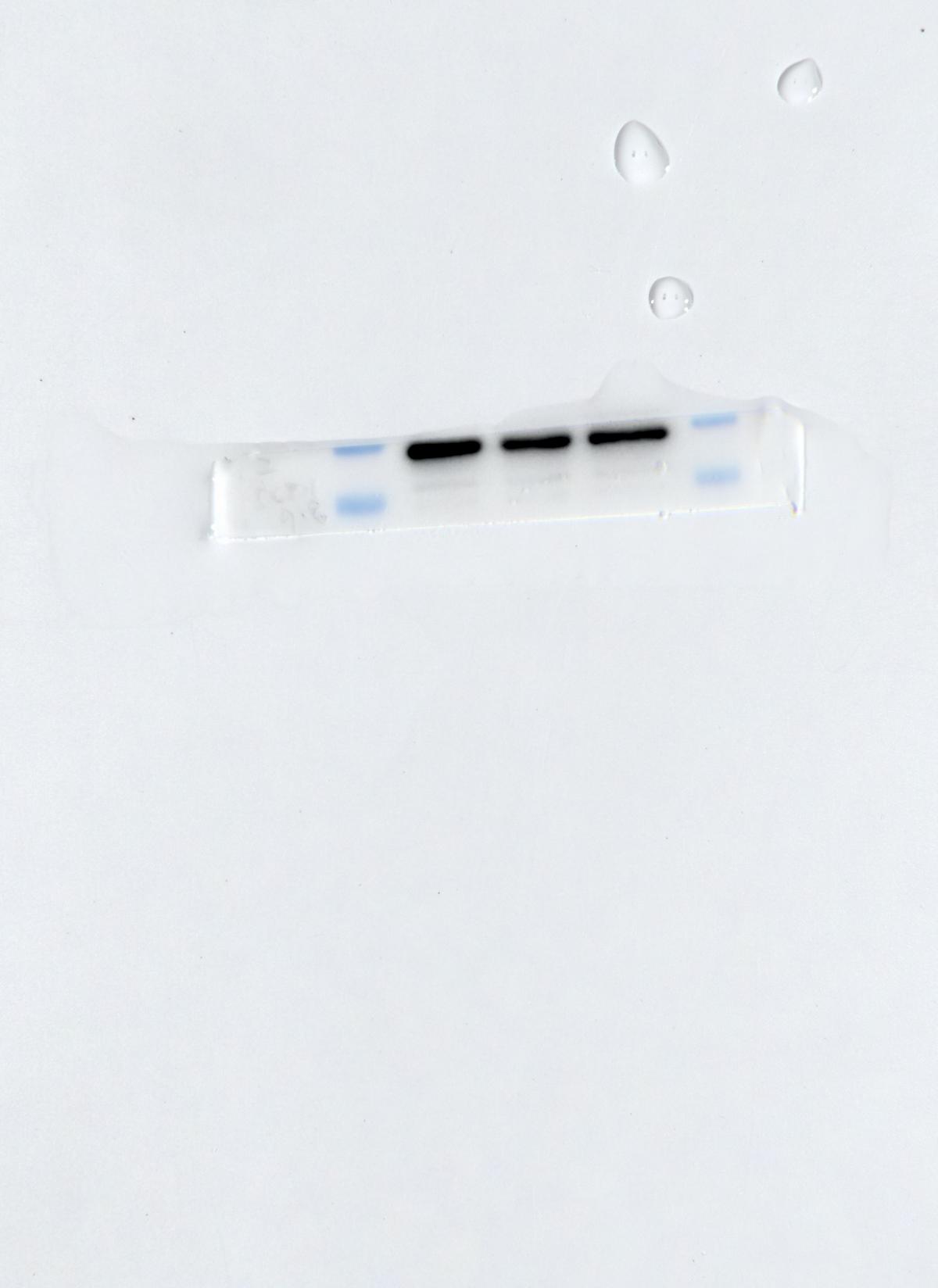


1 2 3

SREBP1


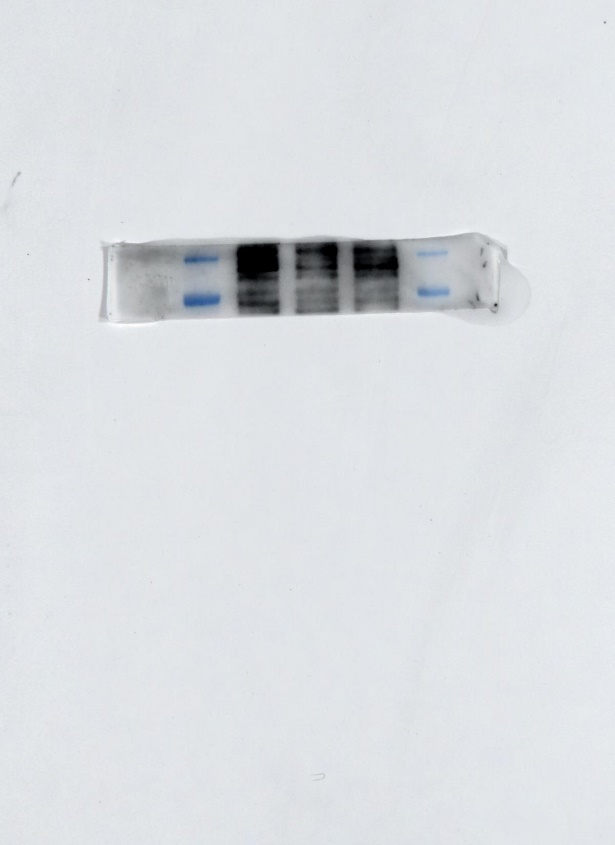

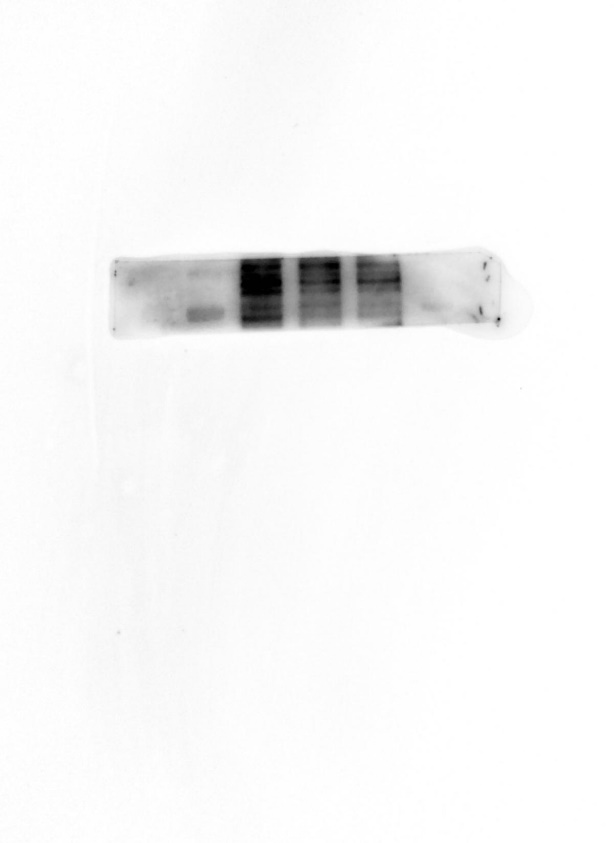


without marker

with marker

130kDa

100kDa

GAPDH


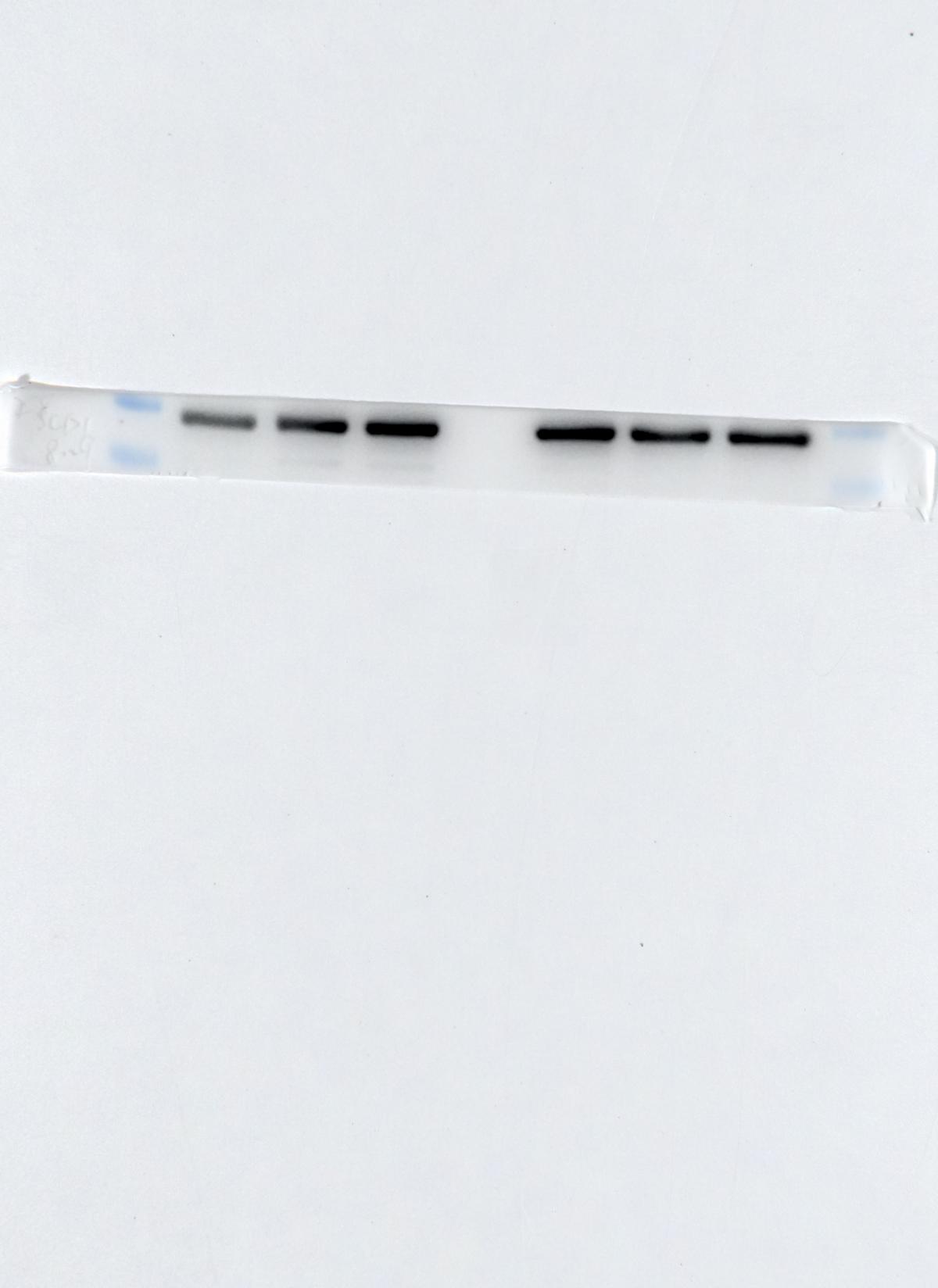


**Figure 6H**

**1: si NC-1**

**2: si SCD-1-1**

**3: si SCD-1-2**

**4: si NC-2**

**5: si SCD-1-1**

**6: si SCD-1-2**

1 2 3 4 5 6

SCD-1


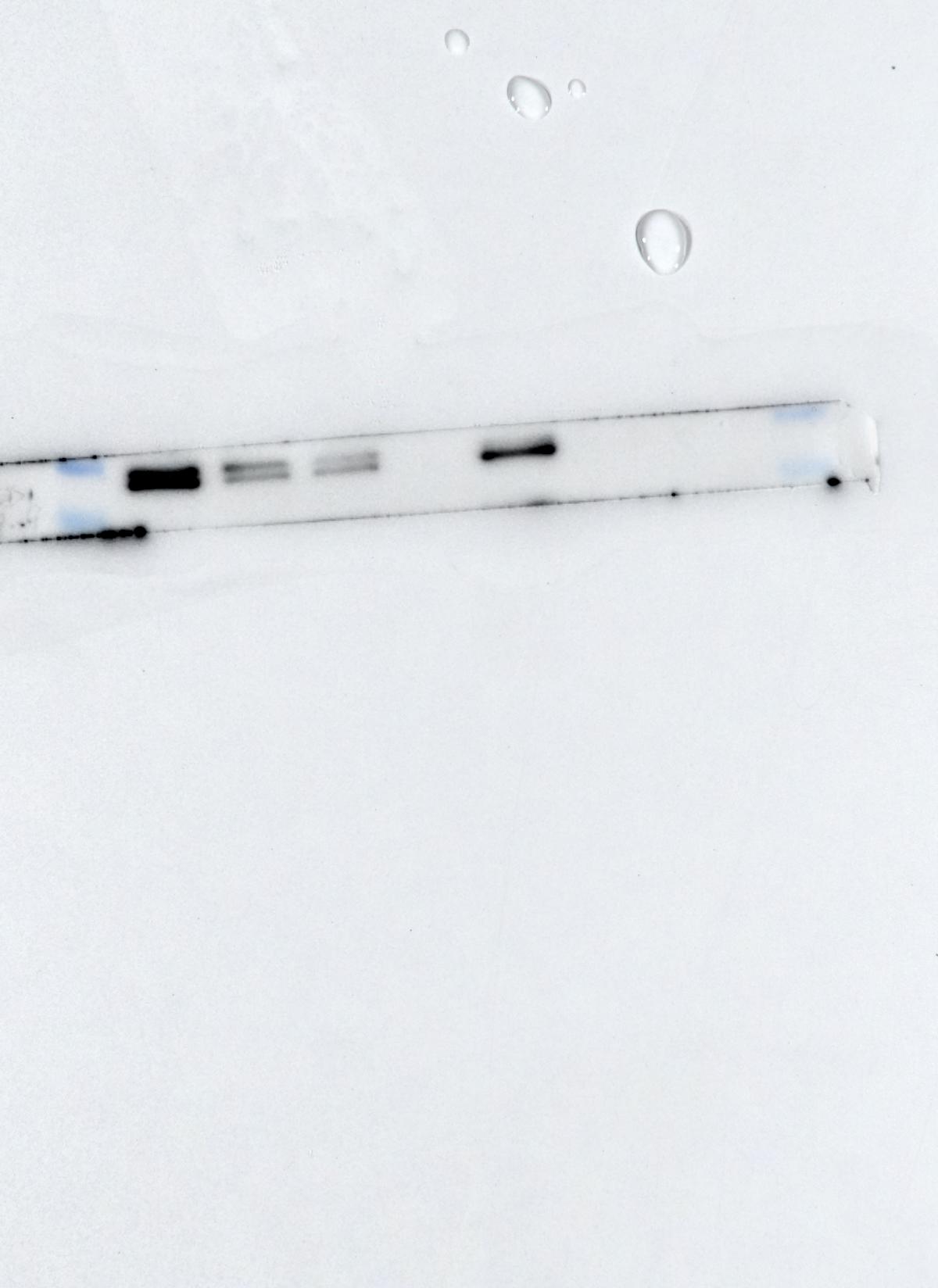


GAPDH

**Supplementary Figure 1G**

**1: OASF1**

**2: OASF2**

**3: OASF3**

**4: RASF1**

**5: RASF2**

**6: RASF3**


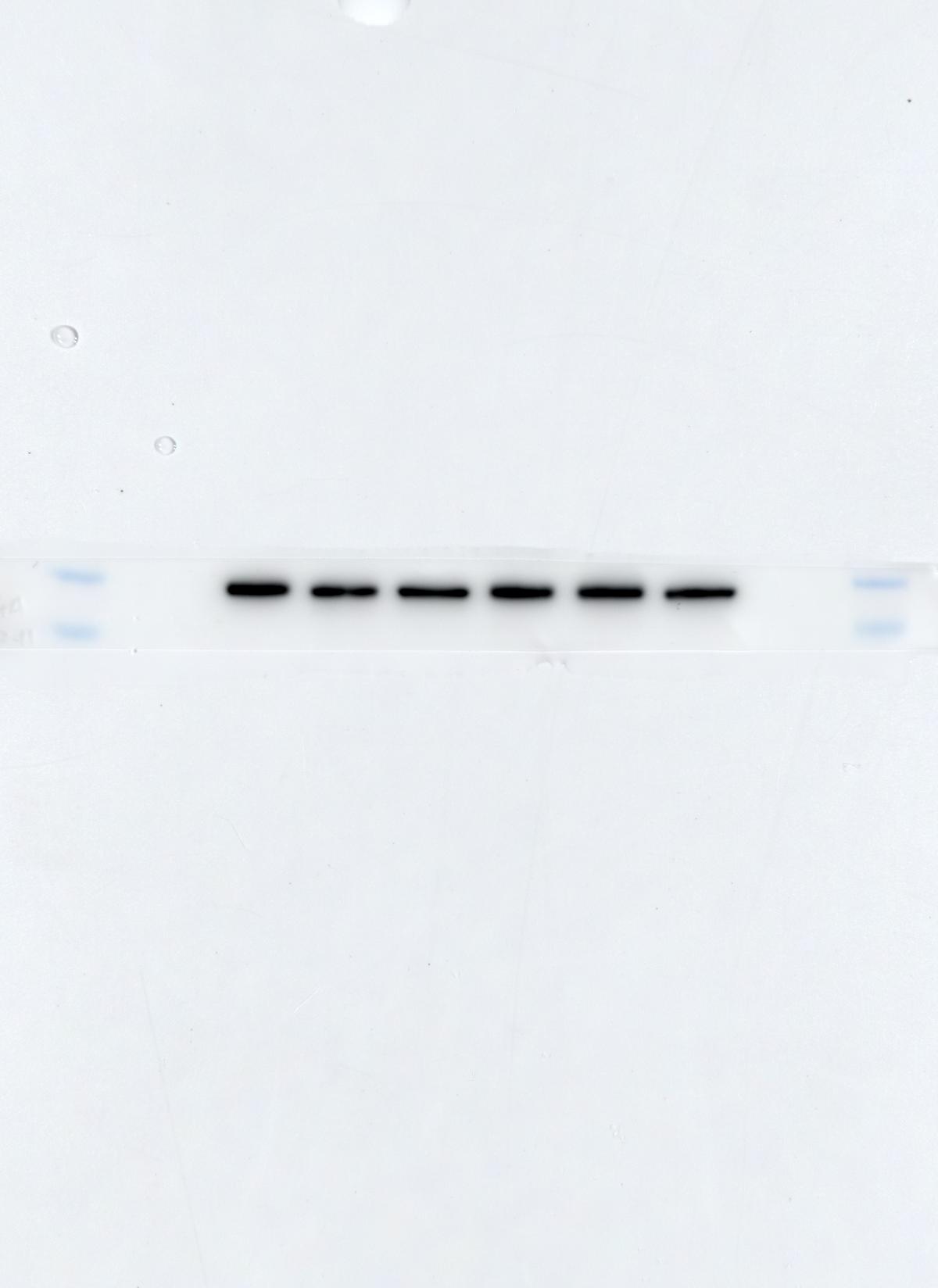


1 2 3 4 5 6

Plexin-A1


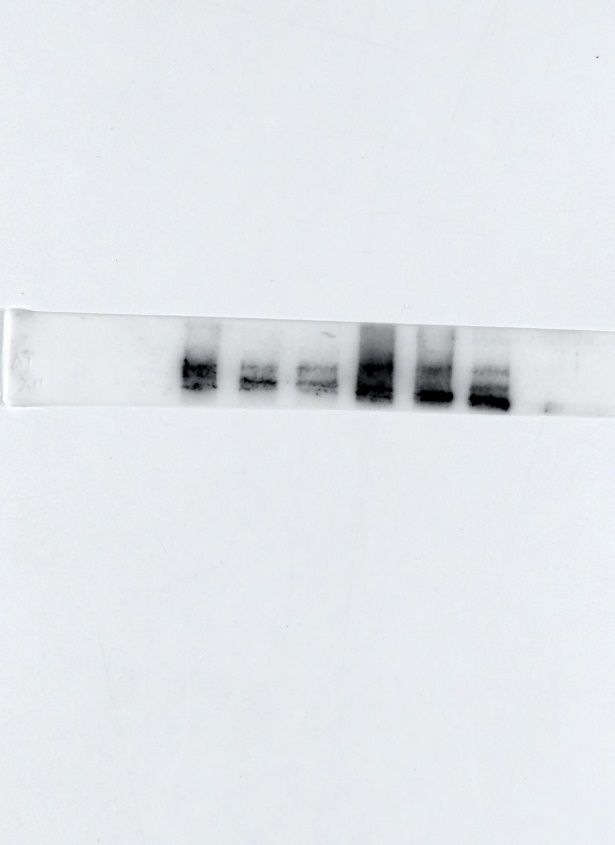

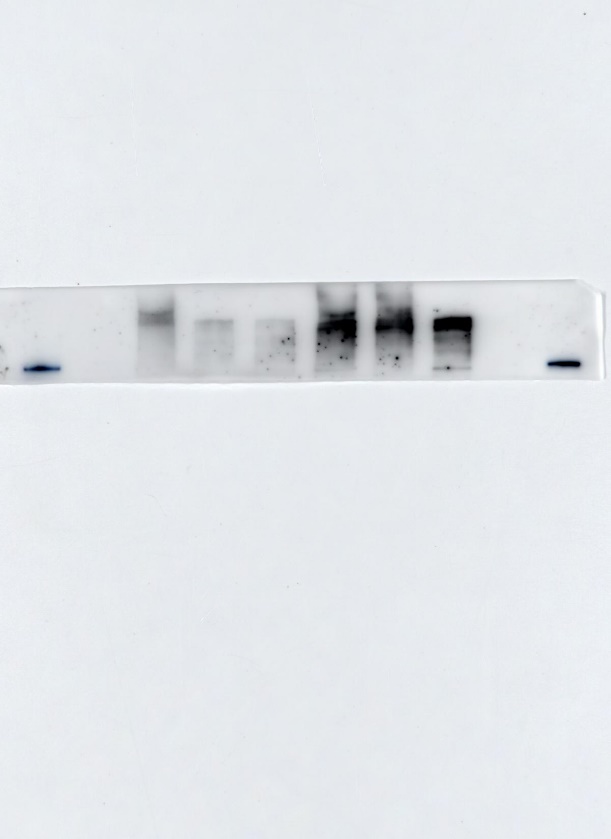


**Another repeat under the same condition with marker (180kDa)**

>180kDa

180kDa

Plexin-B3


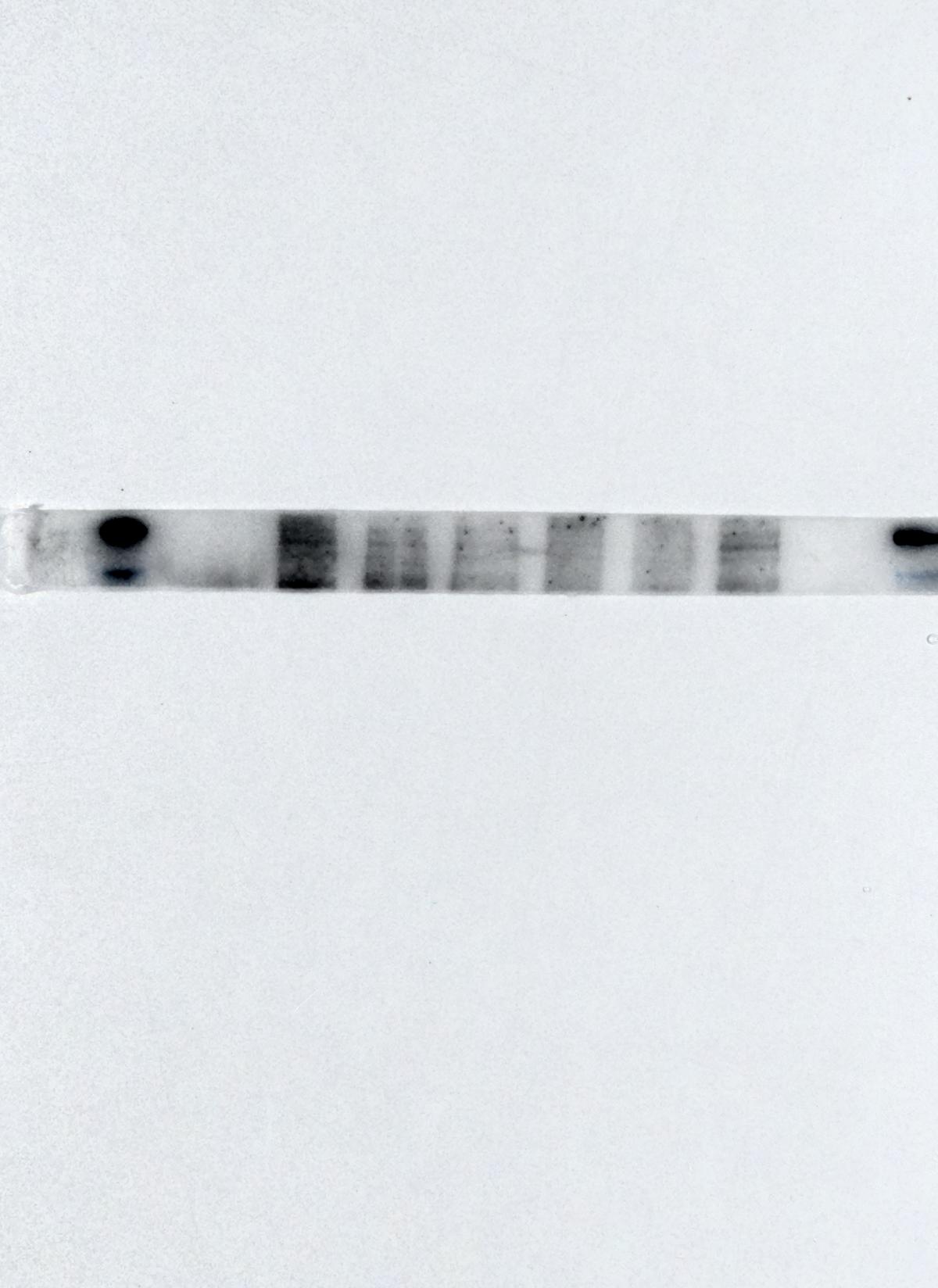


180kDa

130kDa

GAPDH


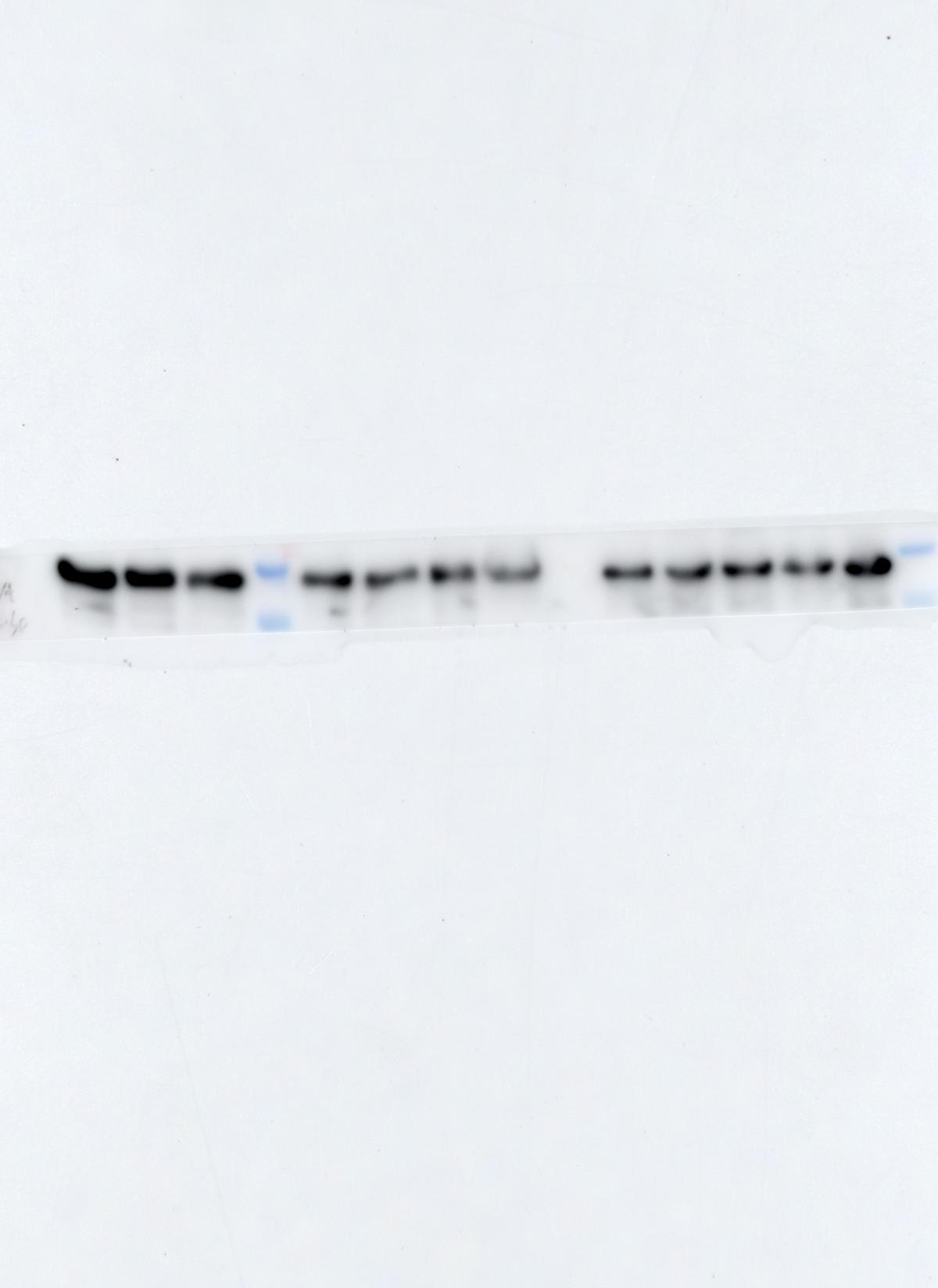


**Supplementary**

**Figure 5A**

**1-3: HC**

**4-7: OA**

**8-12:RA**

1 2 3 4 5 6 7 8 9 10 11 12

Semaphorin 5A


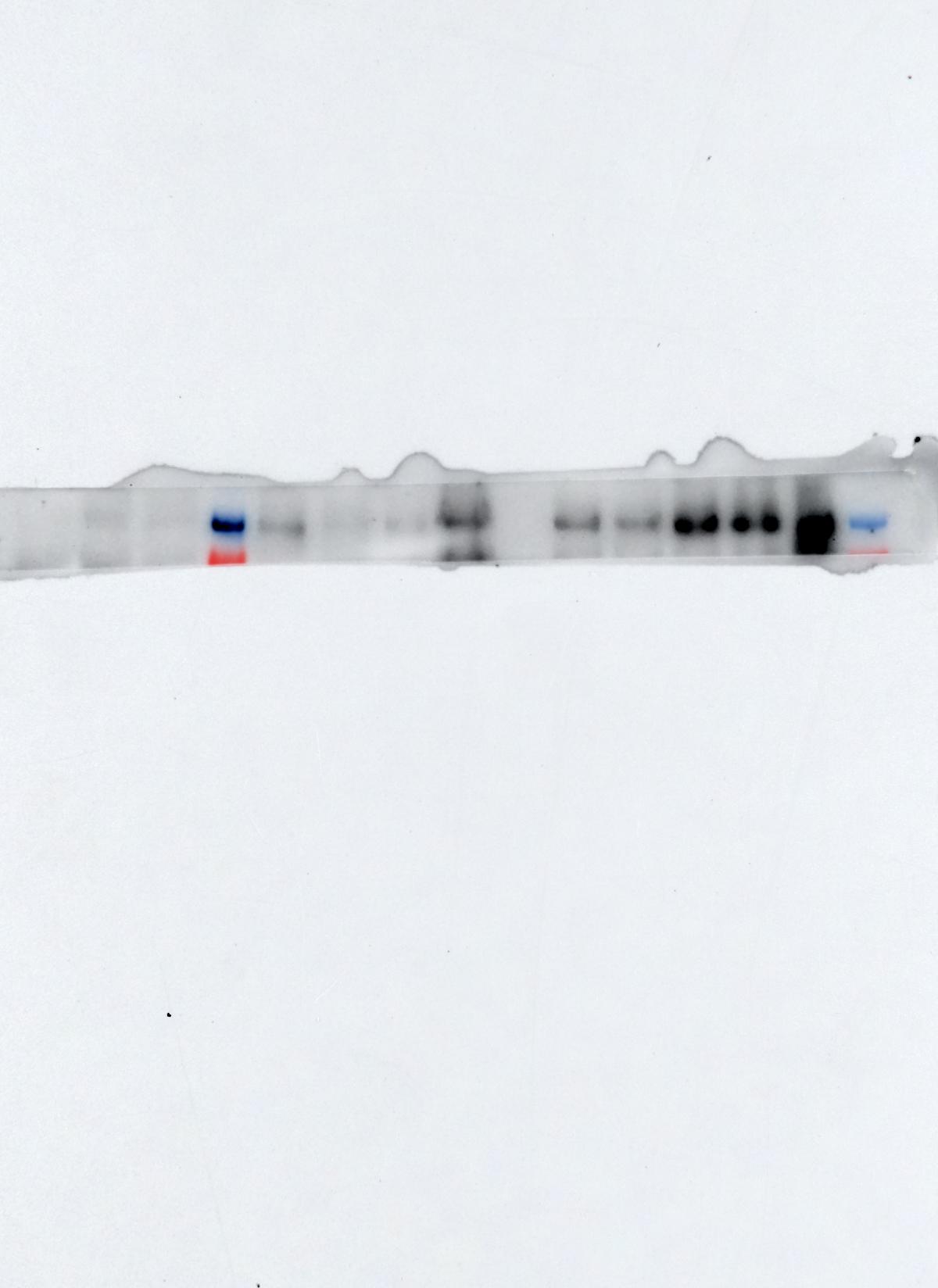


GPX4


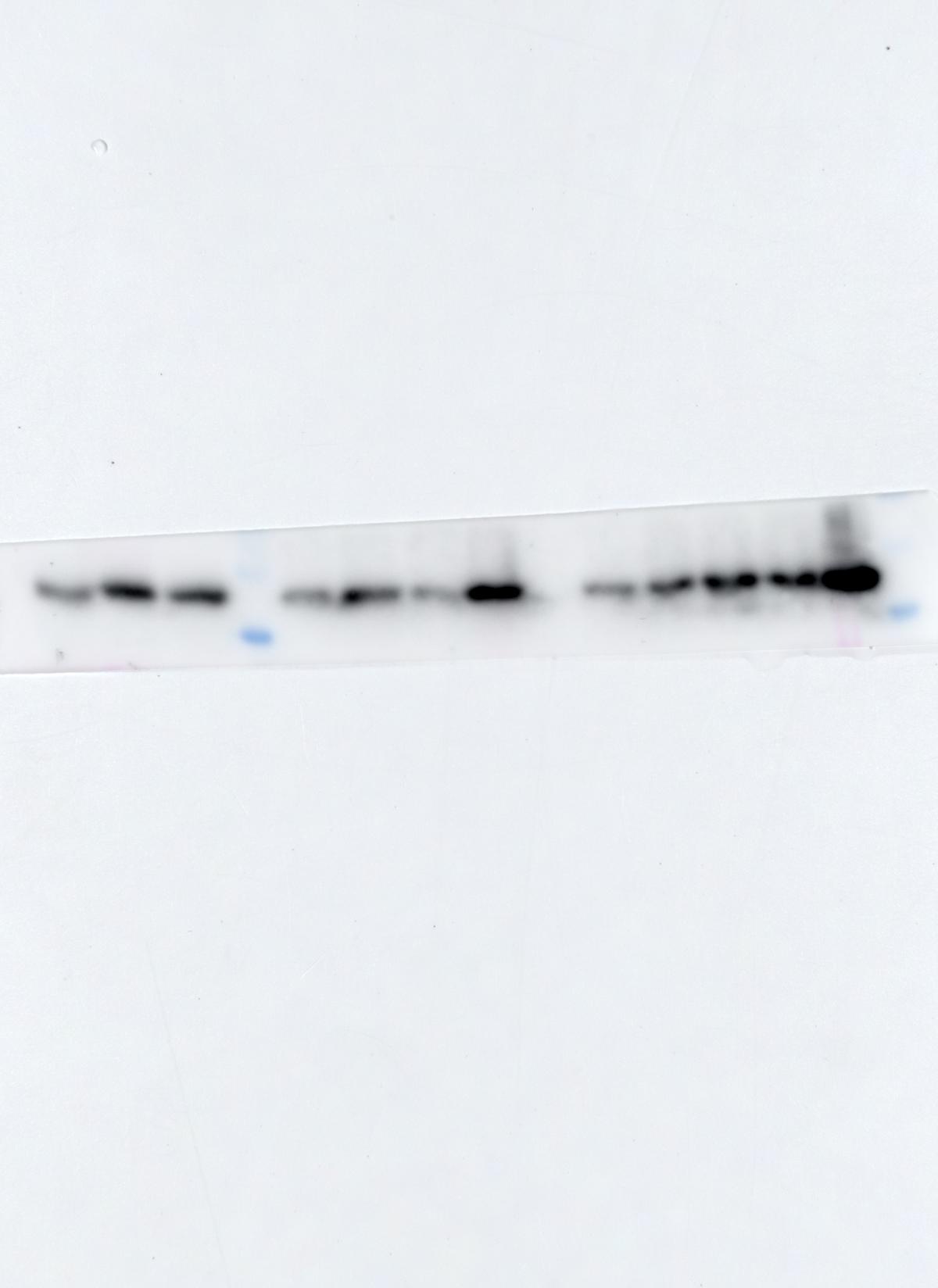


GAPDH


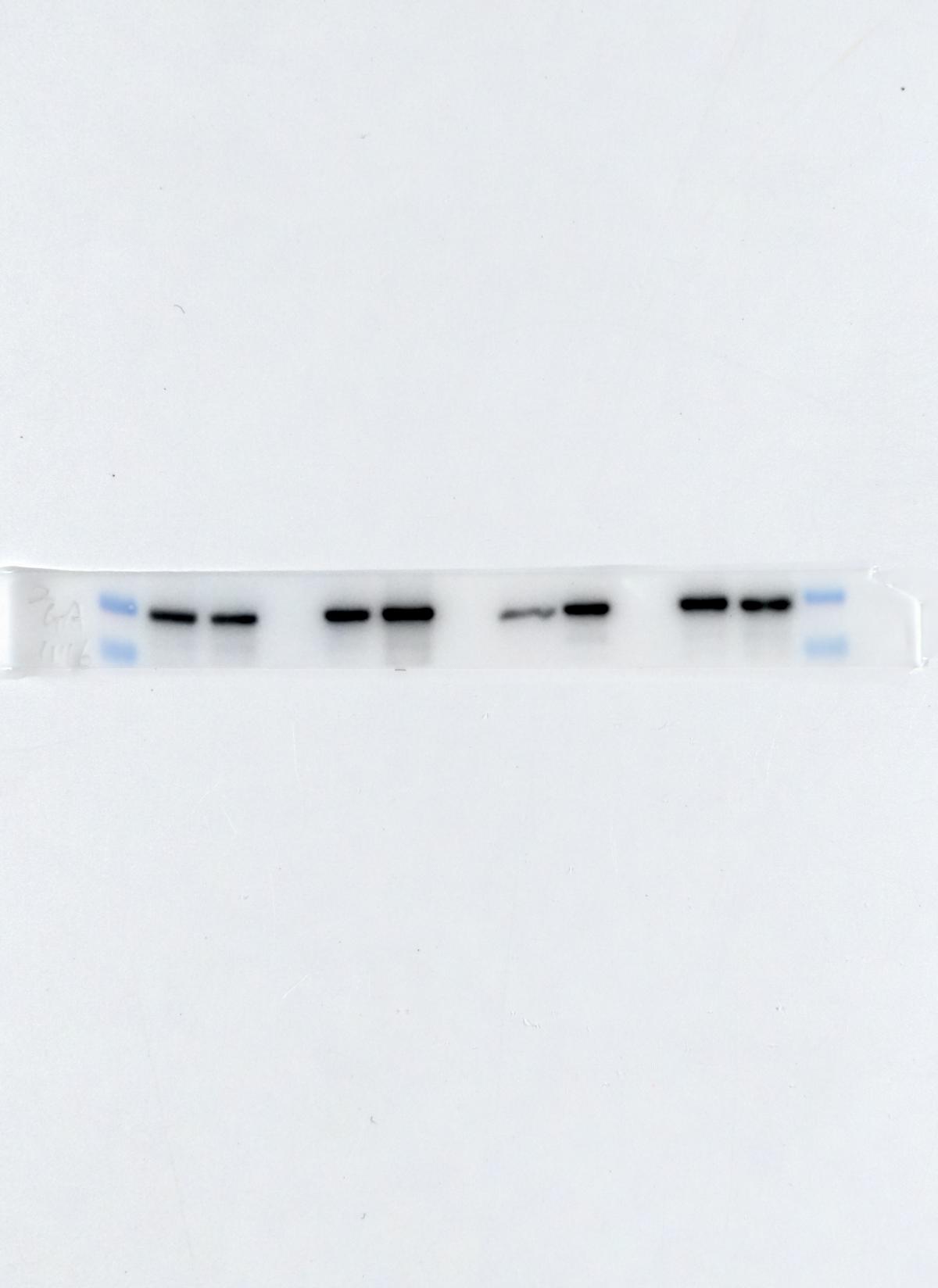


**Supplementary**

**Figure 5A**

**1: OASF5**

**2: RASF5**

**3: OASF6**

**4: RASF6**

**5: OASF7**

**6: RASF7**

**7: OASF8**

**8: RASF8**

1 2 3 4 5 6 7 8

Semaphorin 5A


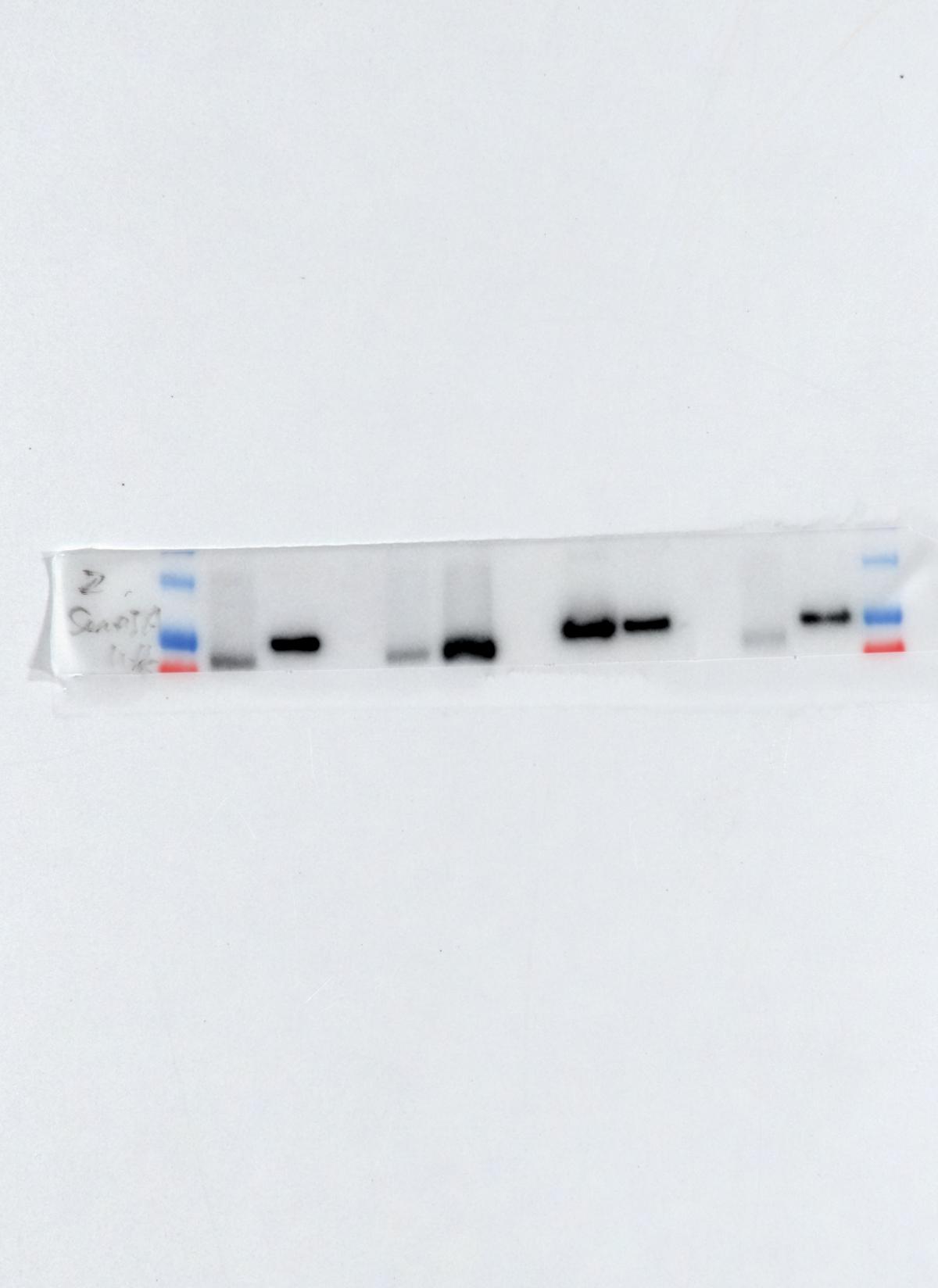


GPX4


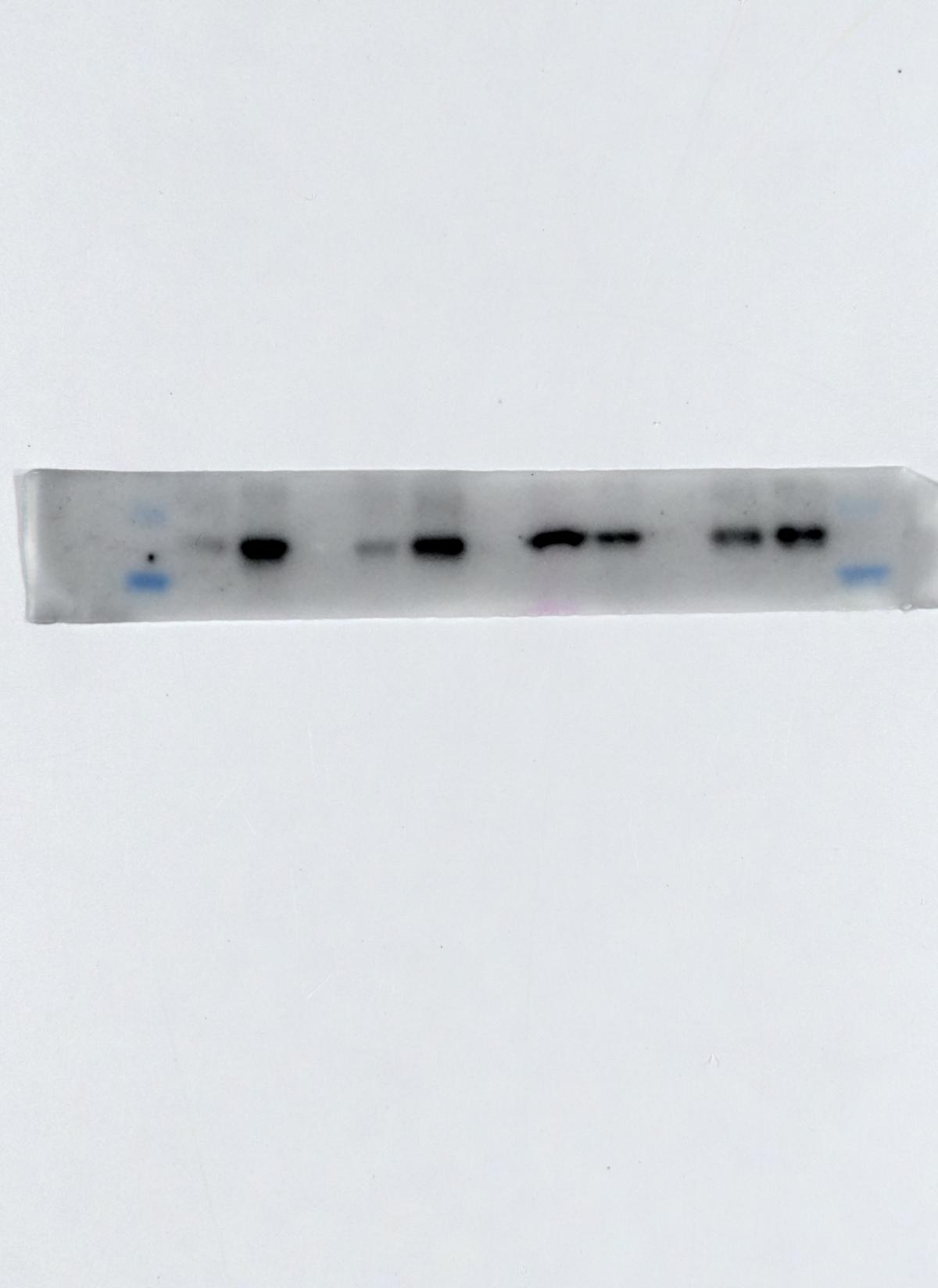


GAPDH


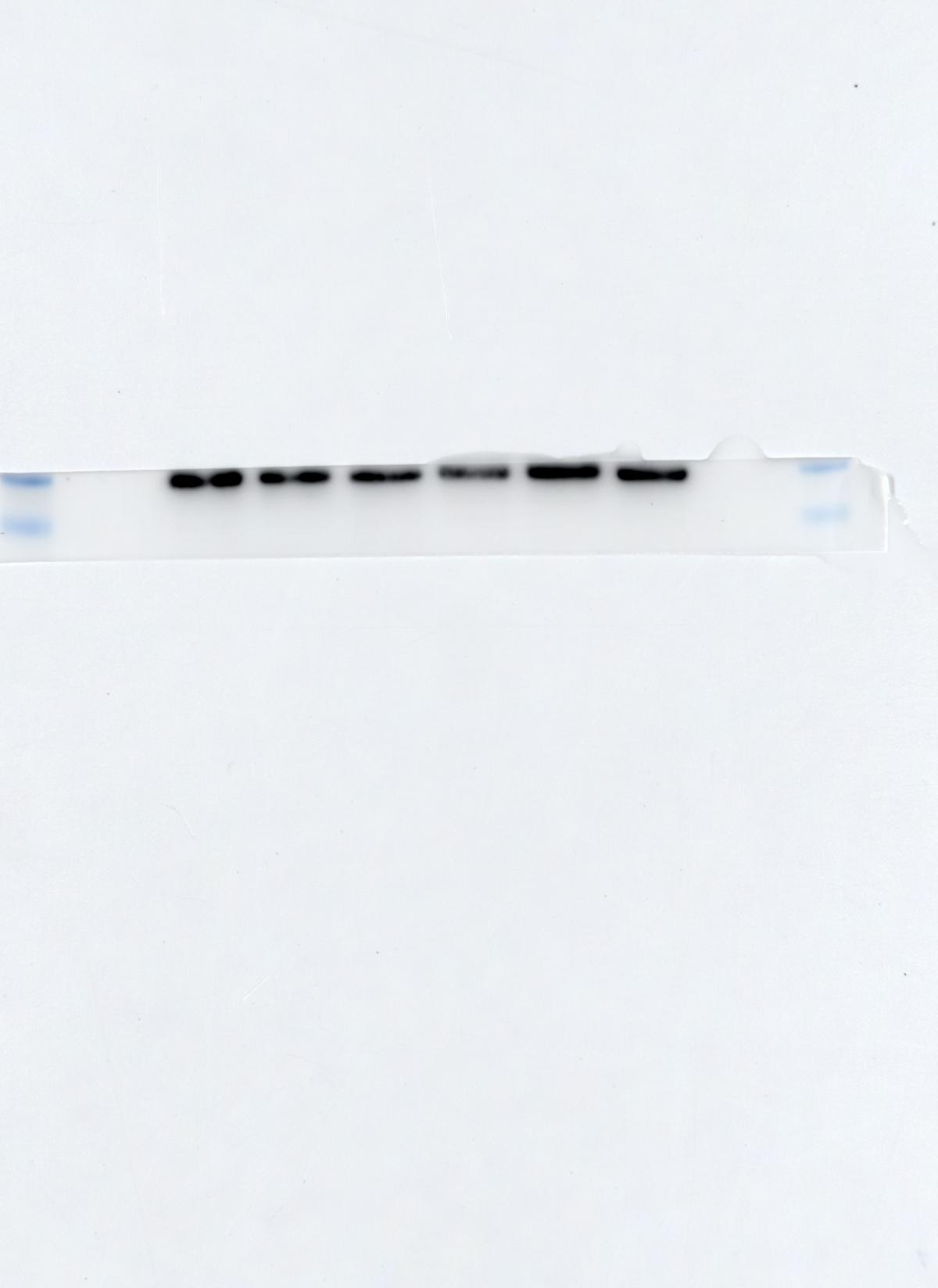


**Supplementary**

**Figure 6C**

**1: OASF1**

**2: OASF2**

**3: OASF3**

**4: RASF1**

**5: RASF2**

**6: RASF3**

SREBP1


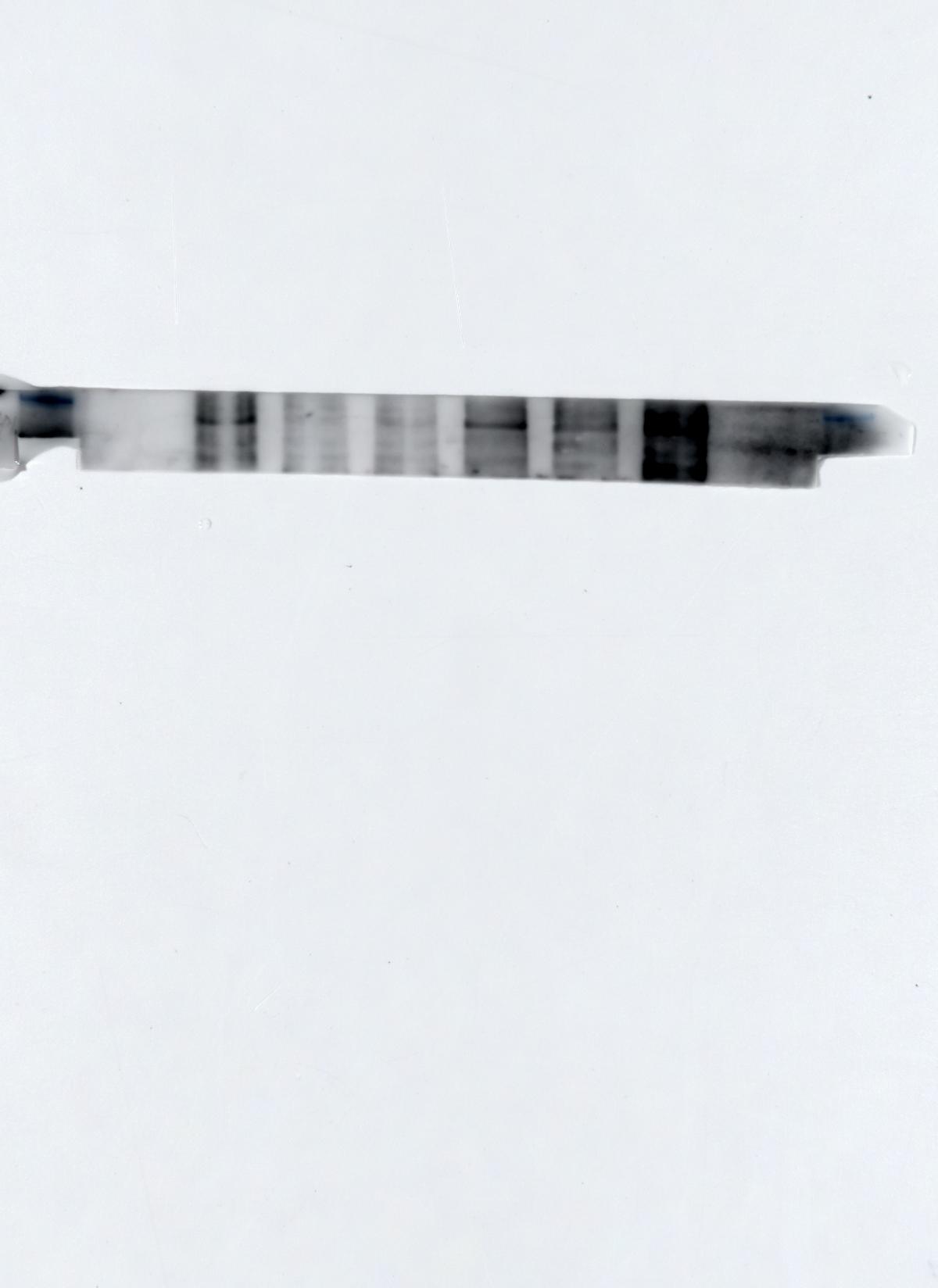


SCD-1


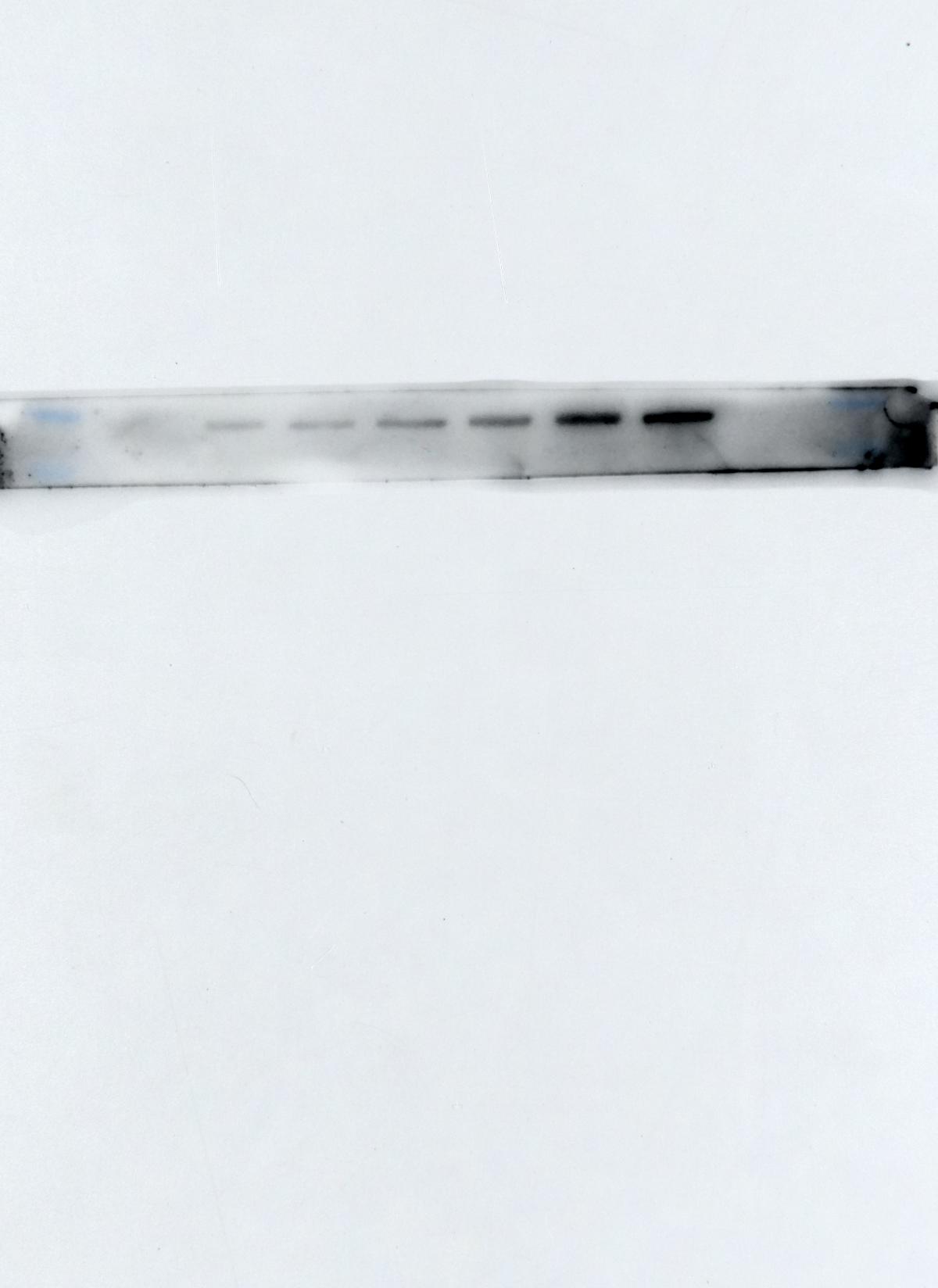

Supplement: Supplementary file 3 — Original Data File [file 41419_2022_5065_MOESM3_ESM.docx]
